# Development of cyclopeptide inhibitors specifically disrupting FXR–coactivator interaction in the intestine as a novel therapeutic strategy for MASH

**DOI:** 10.1093/lifemeta/loaf004

**Published:** 2025-02-08

**Authors:** Yazhou Li, Tingying Jiao, Xi Cheng, Lu Liu, Mengjiao Zhang, Jian Li, Jue Wang, Shulei Hu, Cuina Li, Tao Yu, Yameng Liu, Yangtai Li, Yu Zhang, Chuying Sun, Jina Sun, Jiang Wang, Cen Xie, Hong Liu

**Affiliations:** School of Pharmaceutical Science and Technology, Hangzhou Institute for Advanced Study, University of Chinese Academy of Sciences, Hangzhou, Zhejiang 310024, China; State Key Laboratory of Drug Research, Shanghai Institute of Materia Medica, Chinese Academy of Sciences, Shanghai 201203, China; State Key Laboratory of Drug Research, Shanghai Institute of Materia Medica, Chinese Academy of Sciences, Shanghai 201203, China; State Key Laboratory of Genetic Engineering, School of Life Sciences, Human Phenome Institute, Zhangjiang Fudan International Innovation Center, Metabonomics and Systems Biology Laboratory at Shanghai International Centre for Molecular Phenomics, Zhongshan Hospital, Fudan University, Shanghai 200032, China; School of Pharmaceutical Science and Technology, Hangzhou Institute for Advanced Study, University of Chinese Academy of Sciences, Hangzhou, Zhejiang 310024, China; State Key Laboratory of Drug Research, Shanghai Institute of Materia Medica, Chinese Academy of Sciences, Shanghai 201203, China; State Key Laboratory of Drug Research, Shanghai Institute of Materia Medica, Chinese Academy of Sciences, Shanghai 201203, China; School of Chinese Materia Medica, Nanjing University of Chinese Medicine, Nanjing, Jiangsu 210023, China; State Key Laboratory of Drug Research, Shanghai Institute of Materia Medica, Chinese Academy of Sciences, Shanghai 201203, China; Department of Nephrology, Longhua Hospital, Shanghai University of Traditional Chinese Medicine, Shanghai 201203, China; State Key Laboratory of Drug Research, Shanghai Institute of Materia Medica, Chinese Academy of Sciences, Shanghai 201203, China; State Key Laboratory of Drug Research, Shanghai Institute of Materia Medica, Chinese Academy of Sciences, Shanghai 201203, China; State Key Laboratory of Drug Research, Shanghai Institute of Materia Medica, Chinese Academy of Sciences, Shanghai 201203, China; State Key Laboratory of Drug Research, Shanghai Institute of Materia Medica, Chinese Academy of Sciences, Shanghai 201203, China; School of Pharmaceutical Sciences, Sun Yat-Sen University, Guangzhou, Guangdong 510006, China; State Key Laboratory of Drug Research, Shanghai Institute of Materia Medica, Chinese Academy of Sciences, Shanghai 201203, China; Lingang Laboratory, Shanghai 200031, China; School of Chinese Materia Medica, Nanjing University of Chinese Medicine, Nanjing, Jiangsu 210023, China; State Key Laboratory of Drug Research, Shanghai Institute of Materia Medica, Chinese Academy of Sciences, Shanghai 201203, China; Lingang Laboratory, Shanghai 200031, China; State Key Laboratory of Drug Research, Shanghai Institute of Materia Medica, Chinese Academy of Sciences, Shanghai 201203, China; Lingang Laboratory, Shanghai 200031, China; State Key Laboratory of Drug Research, Shanghai Institute of Materia Medica, Chinese Academy of Sciences, Shanghai 201203, China; School of Chinese Materia Medica, Nanjing University of Chinese Medicine, Nanjing, Jiangsu 210023, China; School of Pharmaceutical Science and Technology, Hangzhou Institute for Advanced Study, University of Chinese Academy of Sciences, Hangzhou, Zhejiang 310024, China; State Key Laboratory of Drug Research, Shanghai Institute of Materia Medica, Chinese Academy of Sciences, Shanghai 201203, China; School of Chinese Materia Medica, Nanjing University of Chinese Medicine, Nanjing, Jiangsu 210023, China

**Keywords:** intestine-targeted drugs, FXR antagonists, cyclopeptide, metabolic dysfunction-associated steatohepatitis, protein–protein interactions

## Abstract

Intestinal farnesoid X receptor (FXR) antagonists have been proven to be efficacious in ameliorating metabolic diseases, particularly for the treatment of metabolic dysfunction-associated steatohepatitis (MASH). All the reported FXR antagonists target to the ligand-binding pocket (LBP) of the receptor, whereas antagonist acting on the non-LBP site of nuclear receptor (NR) is conceived as a promising strategy to discover novel FXR antagonist. Here, we have postulated the hypothesis of antagonizing FXR by disrupting the interaction between FXR and coactivators, and have successfully developed a series of macrocyclic peptides as FXR antagonists based on this premise. The cyclopeptide DC646 not only exhibits potent inhibitory activity of FXR, but also demonstrates a high degree of selectivity towards other NRs. Moreover, cyclopeptide DC646 has high potential therapeutic benefit for the treatment of MASH in an intestinal FXR-dependent manner, along with a commendable safety profile. Mechanistically, distinct from other known FXR antagonists, cyclopeptide DC646 specifically binds to the coactivator binding site of FXR, which can block the coactivator recruitment, reducing the circulation of intestine-derived ceramides to the liver, and promoting the release of glucagon-like peptide-1 (GLP-1). Overall, we identify a novel cyclopeptide that targets FXR-coactivator interaction, paving the way for a new approach to treating MASH with FXR antagonists.

## Introduction

Farnesoid X receptor (FXR) is a well-established bile acid-activated nuclear receptor (NR), which is critical for regulating bile acid or sterol metabolism. Intestinal FXR antagonism has attracted significant attention for treating metabolic dysfunction, with the discovery of FXR antagonists or alternative strategies [1–11]. NRs are a wide family of ligand-regulated transcription factors, sharing a common modular structure, consisting of a non-ligand activation domain at the amino-terminal (N-terminal), a DNA-binding domain (DBD), a flexible hinge region, and a ligand-binding domain (LBD) comprising the ligand-binding pocket (LBP), the activation function 2 (AF2) site, and the binding function 3 (BF3) site. Among these components, the AF2 site, a hydrophobic groove encompassed by residues in α3, α4, and α12, holds significance in the recruitment of coregulators, including the steroid receptor coactivators (SRCs), which facilitate intramolecular interactions between the N-terminal and carboxy-terminal (C-terminal) of the NR. Therefore, we aspire to uncover novel mechanisms of FXR antagonists by targeting FXR–coactivator interaction. Despite the high sequence similarity of AF2 sites among NRs, the distinct surface shapes and electrostatic characteristics of these sites provide opportunities for the design of NR-specific antagonists. For instance, the androgen receptor (AR) AF2 forms a deep hydrophobic groove, leading to the discovery of several AR antagonists that target this site [12–16]. In contrast, the AF2 site in FXR constitutes a shallow and expansive hydrophobic pocket, rendering the identification of small molecules acting at this site challenging. Most reported FXR agonists and antagonists bind to the FXR-LBD. To date, no FXR antagonists have been reported to function by blocking protein–protein interactions (PPIs) between the LBD and coactivators.

Several secondary bile acids, including tauro-β-muricholic acid (T-β-MCA) [1], glycoursodeoxycholic acid (GUDCA) [2, 10], and hyocholic acid (HCA) [4], have been identified as endogenous FXR antagonists that improve metabolic endpoints in mice with established obesity by inhibiting intestinal FXR signaling. These conventional FXR antagonists, which target the LBP site in the LBD, disrupt the interactions between FXR and coactivators by preventing the binding of primary bile acids to the FXR-LBP. However, as their targeting site in the FXR-LBP is distinct from the sites directly involved in essential PPIs, these conventional FXR antagonists can be considered indirect modulators of FXR activity. Consequently, these endogenous FXR antagonists possess intrinsic limitations with regard to their weak antagonistic activity. Although several non-steroidal small molecules exhibit potent FXR antagonistic activity, achieving absolute non-absorbable or non-systemic exposure *in vivo* remains challenging [17, 18]. Notably, antagonism should be restricted to the gut to avoid systemic exposure, which could impede hepatic FXR and potentially lead to cholestasis or hepatocellular carcinoma (HCC). Furthermore, FXR antagonism in the liver is associated with liver injury and cholestasis. Therefore, the continued development of intestine-targeted FXR antagonists is crucial to avoid antagonizing FXR in the liver. Consequently, there is an urgent need to develop novel therapeutics capable of directly disrupting the interactions between FXR and coactivators.

Cyclopeptides have emerged as a particularly valuable tool for regulating PPIs due to their distinctive characteristics, which combine features of both small molecules and proteins. This unique profile has made them a popular choice for the treatment of various diseases owing to their superior safety and tolerability [19–21]. Recent researches suggest that cyclopeptides may also hold promise in the discovery of novel intestine-targeted FXR antagonists, as they have demonstrated efficacy in modulating metabolism and influencing the structure of the intestinal flora [22]. Compared to linear peptides, cyclopeptides offer numerous advantages. The cyclic structure of them imparts strong target binding affinity and selectivity. Additionally, cyclopeptides possess high lipid solubility, making them less likely to penetrate the intestinal epithelia and more prone to intestinal targeting. The inherent structural stability of cyclopeptides ensures resistance to protease hydrolysis, thereby enhancing their stability and duration of action within the gut. Consequently, the development of cyclopeptide as FXR inhibitor is expected to yield more specifically intestine-targeted FXR inhibitors.

In this study, utilizing an AlphaScreen assay to screen our in-house cyclopeptide library, we have identified a series of C4 morpholine-modified tryptophan-containing cyclopeptides with inhibitory activity against FXR. Among them, the most potent FXR antagonist, cyclopeptide DC646, effectively disrupts coactivators from the hydrophobic groove of FXR-LBD, significantly reduces the transcriptional activity of FXR, and exhibits remarkable selectivity against other NRs. Mechanistically, DC646, distinct from other known FXR antagonists, directly interferes with essential PPIs to block coactivator recruitment. Furthermore, DC646 demonstrates the ability to alleviate symptoms related to metabolic dysfunction-associated steatohepatitis (MASH) in mice, relying on its selective inhibition of intestinal FXR. The discovery of DC646 enriches the repertoire of FXR ligands and may serve as a pharmacological tool for investigating the potential therapeutic effects of intestine-targeted drugs. Our notable findings include: (i) the identification of novel cyclopeptide DC646 as the first inhibitor of PPIs between FXR and coactivators; (ii) cyclopeptide DC646 specifically binds to the coactivator binding site of FXR, blocking the recruitment of both coactivator and corepressor of FXR; (iii) the potential therapeutic benefits of DC646 in the treatment of MASH through the reduction of intestine-derived ceramides circulating to the liver and the enhancement of glucagon-like peptide-1 (GLP-1) release; and (iv) a good safety profile. These findings hold implications for the development of novel therapies targeting FXR dysregulation, particularly in the context of metabolic and liver diseases. Given that PPI targets are often considered challenging to drugs due to their elusive nature, the successful application of cyclopeptide structure specificity highlights its potential as a valuable resource for tackling more demanding targets.

## Results

### Design and discovery of cyclopeptide DC646 as a novel FXR antagonist

**Figure 1:**
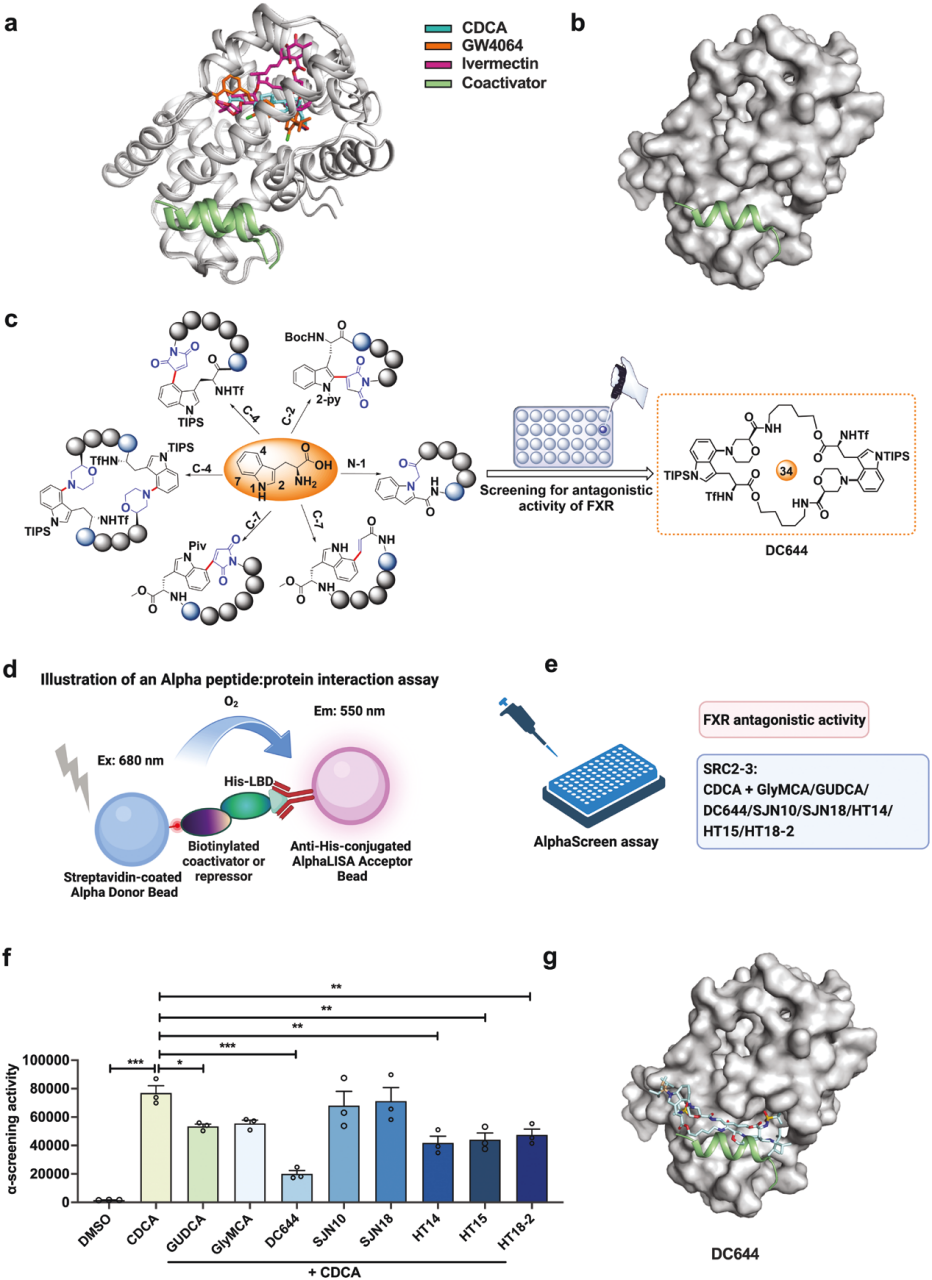
Discovery of cyclopeptide as FXR antagonist. (a) Structures of FXR-LBD complex with agonists (CDCA and GW4064), an antagonist (Ivermectin), and coactivator. The crystal structures (PDB codes: 3DCT, 4WVD, and 6HL1) were structurally aligned with respect to LBD. Agonists and antagonist are shown as sticks. FXR and coactivator are displayed in cartoon. (b) Interfaces of FXR for coactivator and AF2. FXR-LBD is shown as surface. (c) The cyclopeptide library has been screened for antagonistic activity of FXR. (d) Illustration of an alpha protein-protein interaction assay (AlphaScreen assay). (e) Strategy of AlphaScreen assay in (f). (f) GUDCA at 50 μmol/L, GlyMCA at 50 μmol/L, and DC644, SJN10, SJN18, HT14, HT15, and HT18-2 at 10 μmol/L are incubated with His-hFXRα-LBD to test its ability to inhibit recruitment of the SRC2-3 peptide in the presence of CDCA (*n* = 3 in each group). (g) Molecular docking model of DC644 to FXR-LBD (PDB code: 4WVD). Data are presented as mean ± SEM and analyzed by one-way ANOVA with Dunnett’s *post hoc* test for (f). ^*^P ≤ 0.05, ^**^P ≤ 0.01, and ^***^P ≤ 0.001.

Most reported FXR agonists and antagonists bind to the FXR-LBD (Fig. 1a). We tried to discover an inhibitor by blocking PPIs between the LBD and coactivators (Fig. 1b). We assumed that an inhibitor mimicking the coactivators would affect the interaction of the coactivator with the FXR-LBD. Such inhibitors should have comparable sizes to the coactivators. In the structures of FXR, the sizes of the FXR-bound coactivators are larger than 20.0 Å (Supplementary Table S1). Because cyclopeptides have been proven to be a particularly useful tool for regulating PPIs due to their high potency and selectivity, we assumed that they might serve as potential scaffolds for regulating the FXR-coactivator interface. Recently, we have developed a systematic methodology to modify tryptophan and tryptophan-containing peptides and constructed a cyclopeptide library via transition-metal-catalyzed C-H activation of tryptophan or indole at the C-2, C-4, or C-7 positions [23–27] (Fig. 1c). We predicted the sizes of representative peptides in our in-house cyclopeptide library. A novel fused tryptophan C-4 amino-modified cyclopeptide DC644 was predicted to have a size larger than 20.0 Å (Supplementary Table S2). We assumed that cyclopeptides may be better mimics of the coactivators. Therefore, we tested the cyclopeptides screened by using the AlphaScreen assay (Fig. 1d and e). Compared with the endogenous FXR antagonist GUDCA and synthesized FXR antagonist glycine-β-muricholic acid (GlyMCA), cyclopeptide DC644 was more potent towards FXR (Fig. 1f). Molecular docking of DC644 to FXR indicated that DC644 bound to the surface of FXR-LBD, occupying the position of coactivator (Fig. 1g).

**Figure 2:**
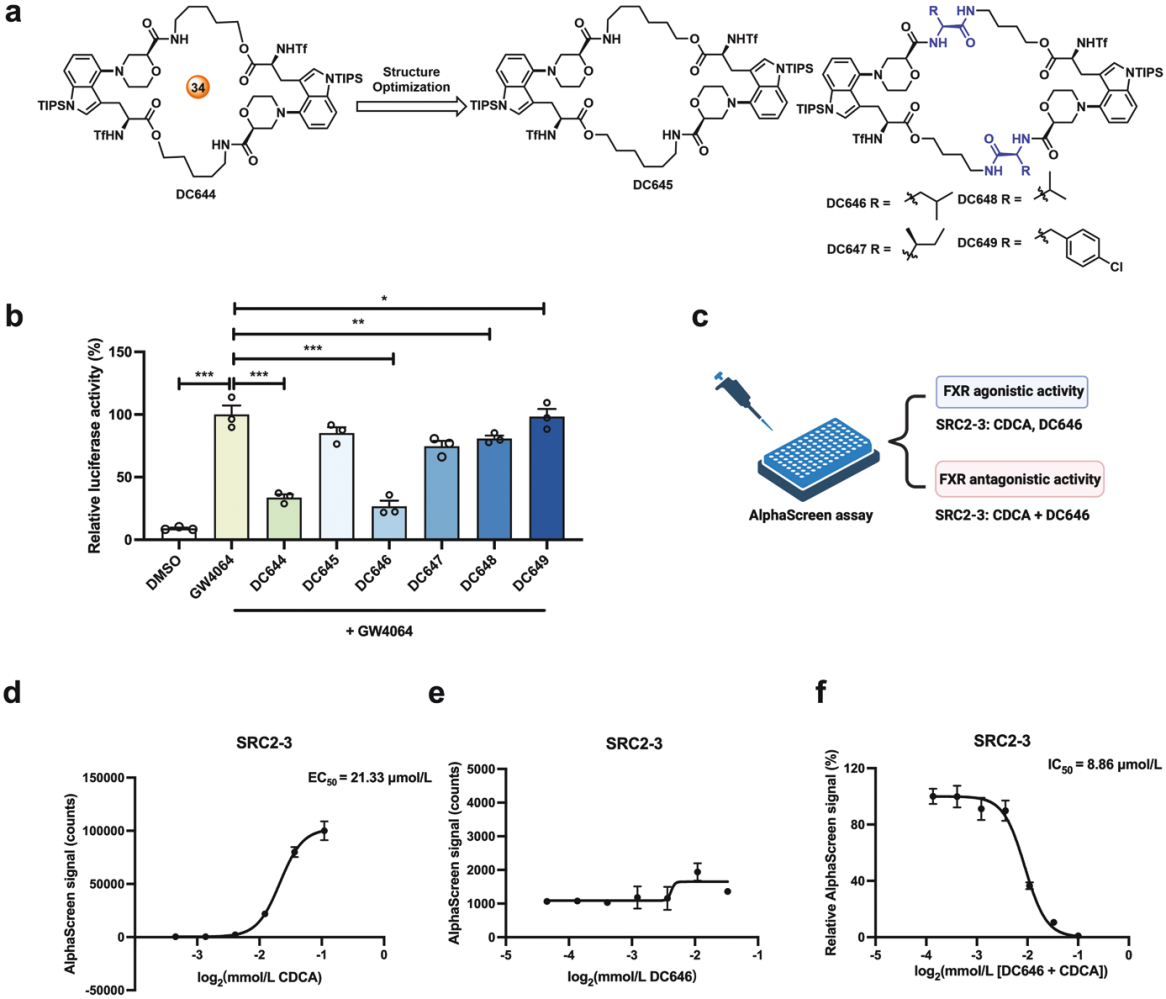
Cyclopeptide DC646 as a potent FXR antagonist. (a) The rational design of the target cyclopeptides DC645–DC649. (b) Relative luciferase activity of the transfected HEK293T cells treated with vehicle or tested cyclopeptides at 100 µmol/L in the presence of GW4064 (*n* = 3 in each group). (c) Strategy of AlphaScreen assay in (d–f). (d and e) Various concentrations of CDCA are incubated with His-hFXRα-LBD to obtain the EC_50_ of CDCA (d) or DC646 (e). (f) Various concentrations of DC646 are incubated with His-hFXRα-LBD to obtain the IC_50_ of DC646 in recruiting the SRC2-3 peptide in the presence of CDCA (*n* = 3 in each group). Data are presented as mean ± SEM and analyzed by one-way ANOVA with Dunnett’s *post hoc* test for (b). ^*^P ≤ 0.05, ^**^P ≤ 0.01, and ^***^P ≤ 0.001.

To discover cyclopeptide with more potent FXR antagonistic activity, we retained C4 morpholine-modified tryptophan-containing cyclopeptide scaffold and carried out structural optimization of the size and side chains of cyclopeptide to design five cyclopeptides DC645–DC649 (Fig. 2a). Then, they were synthesized as shown in Supplementary Figs. S1−S3. First, the actions of DC644–DC649 on FXR were evaluated by reporter assay. In the presence of the FXR agonist GW4064, DC646 strongly suppressed FXR activation by more than 70%, better than DC644 (Fig. 2b). The results were further validated by the AlphaScreen assay (Fig. 2c). Unlike the strong FXRα agonist chenodeoxycholic acid (CDCA, the median effective concentration [EC_50_] = 21.33 μmol/L), DC646 alone could hinder human FXR alpha (hFXRα)-LBD to recruit the coactivator SRC2-3 (Fig. 2d and e), indicating that DC646 had no agonistic activity. In the presence of CDCA, DC646 hindered the recruitment of SRC2-3, with a half-maximal inhibitory concentration (IC_50_) value of 8.86 μmol/L (Fig. 2f). Hence, cyclopeptide DC646 was identified as a better compound for FXR antagonist than DC644.

### Cyclopeptide DC646 exerts FXR antagonistic effects by interacting with coactivator

**Figure 3:**
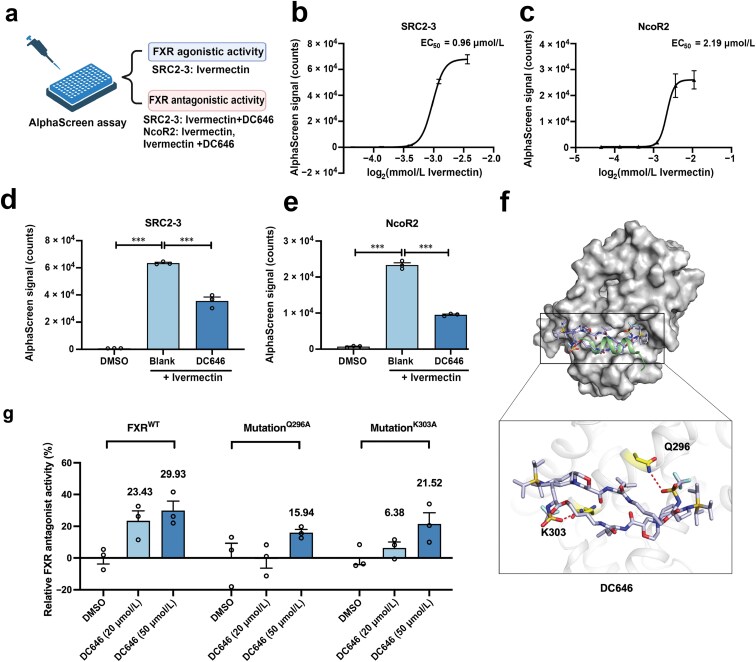
DC646 exerts FXR antagonistic effects by interacting with coactivator via Q296 and K303. (a) Strategy of the AlphaScreen assay in (b) and (c). (b and c) Various concentrations of Ivermectin are incubated with His-hFXRα-LBD to obtain the EC_50_ of Ivermectin in recruiting the peptides of SRC2-3 (b) or NcoR2 (c) (*n* = 3 in each group). (d and e) DC646 at 11 μmol/L is incubated with His-hFXRα-LBD to test its inhibitory ability in recruiting the peptide SRC2-3 (d) or NcoR2 (e) in the presence of Ivermectin (*n* = 3 in each group). (f) Molecular docking model of DC646 to FXR-LBD (PDB code: 4WVD). The key residues Q296 and K303 and DC646 are shown as sticks. (g) Effects of FXR key residue mutations on its transcriptional activity in response to DC646 by reporter assay (*n* = 3 in each group). Data are presented as mean ± SEM and analyzed by one-way ANOVA with Dunnett’s *post hoc* test for (d) and (e). ^***^P ≤ 0.001.

To explore the molecular mechanism underlying DC646-mediated FXR antagonism, we determined the structure of the DC646 by X-ray crystal analysis (Supplementary Fig. S5). We also made efforts to co-crystalize DC646 with hFXRα-LBD. However, these attempts failed due to the poor water solubility of DC646. As an alternative, the AlphaScreen assay was conducted to investigate the mechanism (Fig. 3a). In contrast to CDCA or other known FXR ligands, Ivermectin is a unique FXR ligand with a distinct binding mode in the FXR-LBP, which enhances the flexibility of the α12 helix to recruit both coactivator and corepressor [28]. Consistently, Ivermectin stimulated the hFXRα-LBD to recruit both the coactivator SRC2-3 (EC_50_ = 0.96 μmol/L, Fig. 3b) and the corepressor NcoR2 (EC_50_ = 2.19 μmol/L, Fig. 3c), while DC646 competitively disrupted both hFXRα-LBD-SRC2-3 and hFXRα-LBD-NcoR2 interactions induced by Ivermectin (Fig. 3d and e). These results demonstrated that DC646 is a targeted FXR antagonist with a molecular mechanism by acting as a PPI inhibitor to hinder the recruitment of both coactivator and corepressor to hFXRα-LBD.

To gain further insight into the antagonistic mechanism of DC646, we conducted molecular docking to predict the binding mode of DC646 to FXR-LBD. Similar to DC644, the docking model showed that DC646 also occupied the coactivator binding pocket. It has been reported that in complex with agonists, FXR-LBD interacts with coactivator via interactions involving K303, H313, E314, K321, and E467 of the FXR-LBD [29]. Consistently, putative hydrogen bonds were also observed between DC646 and Q296 or K303 in the docking model (Fig. 3f). These interactions indicated that DC646 well occupies the coactivator-binding site of FXR-LBD. To verify this binding model, we mutated Q296 and K303 on α3 helix to alanine followed by a reporter assay (Fig. 3g). The single-point mutation of Q296 and K303 significantly abolished the FXR trans-repression activity of DC646. This mutation experiment supports our assumption that the surface of FXR-LBD can act as a binding pocket for the cyclopeptide DC646 to exert antagonistic effects.

**Figure 4:**
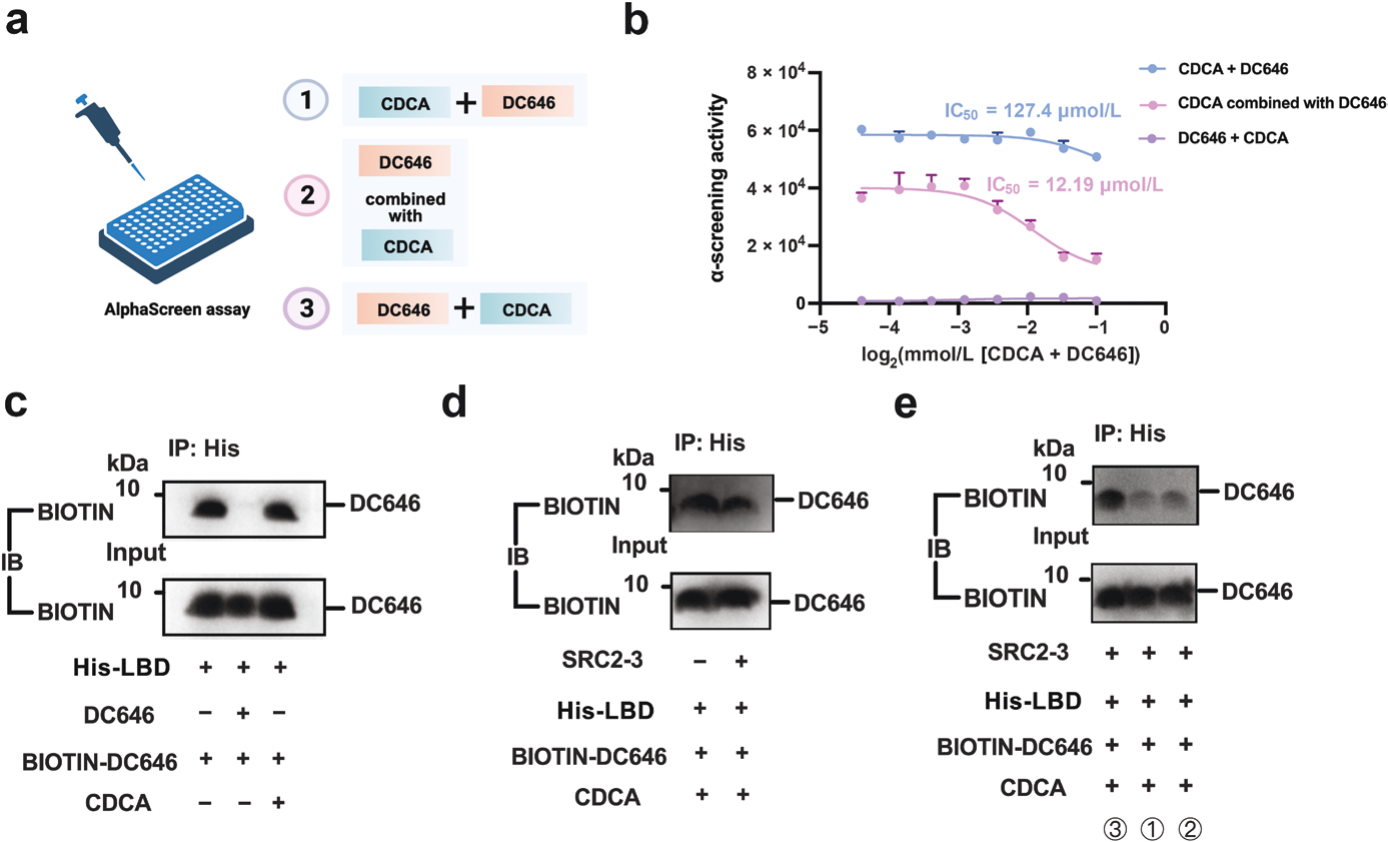
DC646 antagonizes FXR via a novel molecular mechanism. (a) Strategy of the AlphaScreen assay in (b). (b) IC_50_ of DC646 in recruiting the peptides of SRC2-3 under different adding orders of DC646, adding DC646 after CDCA, DC646 combined with CDCA, or CDCA after DC646, which have been incubated with His-hFXRα-LBD, to test its ability to inhibit recruitment of the SRC2-3 peptide (*n* = 3 in each group). (c) DC646 and CDCA bind to FXR-LBD by different binding sites. BIOTIN-DC646 (the synthesis of BIOTIN-DC646 is described in Supporting Information, Supplementary Fig. S4) and the purified FXR-LBD were incubated together with or without CDCA or DC646. These mixtures were immunoprecipitated with His beads and then immunoblotted with anti-BIOTIN antibodies. (d) DC646 antagonizes FXR by interfering FXR-LBD with the coactivator. In the existence of CDCA and the purified FXR-LBD, BIOTIN-DC646 was incubated with or without SRC2-3. These mixtures were immunoprecipitated with His beads and then immunoblotted with anti-BIOTIN antibodies. (e) DC646 antagonizes FXR by binding to the surface of FXR-LBD, thereby inhibiting the binding of FXR-LBD and coactivators. In the existence of SRC2-3 and the purified FXR-LBD, BIOTIN-DC646 was incubated with CDCA in different adding orders indicated by (a). These mixtures were immunoprecipitated with His beads and then immunoblotted with anti-BIOTIN antibodies.

To confirm this finding, an AlphaScreen assay was carried out by adding DC646 after CDCA, DC646 combined with CDCA, or CDCA after DC646 (Fig. 4a). The results showed that DC646 has antagonistic activity with an IC_50_ at 12.19 μmol/L when combined with CDCA. Moreover, adding DC646 before CDCA completely blunted the agonism of CDCA, and adding DC646 after CDCA showed no antagonism (Fig. 4b), indicating that DC646 antagonizes FXR by binding to the surface of FXR-LBD, thereby inhibiting the binding of FXR-LBD and coactivators. The PPI inhibitory effect of DC646 was further confirmed by co-immunoprecipitation assay. DC646 can directly bind with FXR-LBD, and addition of CDCA did not affect the binding of DC646 with FXR-LBD, implying different binding sites of DC646 and CDCA to FXR-LBD (Fig. 4c). Furthermore, SRC2-3 mediated the disruption of DC646 binding to FXR-LBD in the existence of CDCA (Fig. 4d), indicating that DC646, as a PPI inhibitor, antagonizes FXR by interfering FXR-LBD with the coactivator. Consistent with the AlphaScreen assay, adding CDCA after DC646 showed better binding capacity of DC646 with FXR-LBD comparing with DC646 combined with CDCA, and DC646 after CDCA largely blunted the binding of DC646 with FXR-LBD (Fig. 4e).

### DC646 attenuates the pathologies of methionine- and choline-deficient (MCD) diet-induced MASH mice via antagonism of intestinal FXR

**Figure 5:**
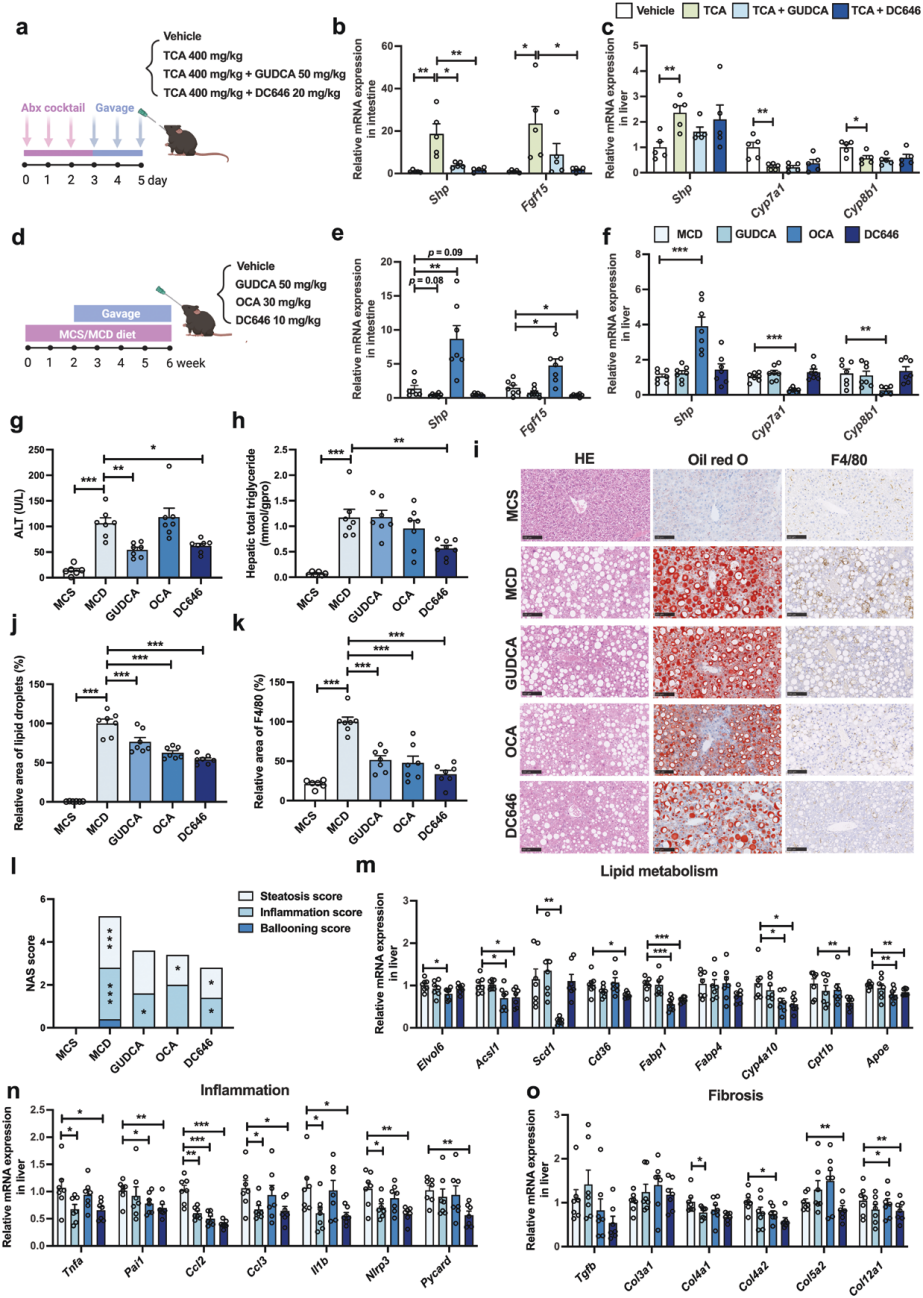
DC646 attenuates the pathologies of MCD-induced MASH mice. (a) Strategy of animal experiments. (b and c) Relative mRNA levels of the intestinal (b) or hepatic (c) FXR signaling (*n* = 5 in each group). (d) Strategy of animal experiments. Mice have been fed an MCS diet or an MCD diet to induce MASH for 2 weeks, before being orally administered with vehicle, GUDCA, OCA, or DC646 for 4 weeks. (e and f) Relative mRNA levels of the intestinal (e) or hepatic (f) FXR signaling. (g) ALT levels. (h) Hepatic TG contents. (i) Representative images of H&E, Oil Red O, and F4/80 staining. (j) Quantification of Oil Red O staining. (k) Quantification of F4/80 staining. (l) NAS score of each group. (m) Relative mRNA levels of lipid metabolism-related genes. (n) Relative mRNA levels of inflammation-related genes. (o) Relative mRNA levels of fibrosis-related genes. *n* = 6–7 in each group. Data are presented as mean ± SEM and analyzed by one-way ANOVA with Dunnett’s *post hoc* test for (b), (c), (e–h) and (j–o). ^*^P ≤ 0.05, ^**^P ≤ 0.01, and ^***^P ≤ 0.001.

Subsequently, the potential regulation of FXR signaling by DC646 was evaluated in intestinal organoids and in mice (Fig. 5a; Supplementary Fig. S6a and b). The results showed that DC646 effectively reversed the mRNA expression of the GW4064-induced FXR target gene small heterodimer partner (*Shp*) in the organoid culture, but this effect was not observed in *Fxr* intestinal knockout (*Fxr*^*△IE*^) organoids, indicating the FXR-dependent pattern. Further *in vivo* results also demonstrated that DC646 decreased the mRNA expression levels of taurocholic acid (TCA)-induced FXR target genes *Shp* and fibroblast growth factor 15 (*Fgf15*), but not hepatic FXR signaling (Fig. 5b and c). Moreover, tissue distribution monitoring revealed that DC646 was retained in the intestine (Supplementary Table S3), while the target selectivity study demonstrated that DC646 had no significant effect on the transactivation or trans-repression of the peroxisome proliferator-activated receptor (PPAR) α, PPARδ, and PPARγ (Supplementary Table S4). Furthermore, DC646 showed no significant impact on pregnane X receptor (*Pxr*), vitamin D receptor (*Vdr*), and constitutive androstane receptor (*Nr1i3*) target gene expression in organoids (Supplementary Fig. S7). Collectively, the above results demonstrate that DC646 is a highly selective intestinal FXR antagonist.

Studies have demonstrated that targeting intestinal FXR is a promising approach for treating metabolic diseases, such as obesity, MASH, and diabetes. In addition, intestine-restricted FXR antagonists may ameliorate obesity-related dysfunction and circumvent the potential adverse outcomes resulting from the inhibition of liver FXR. Given that DC646 has been identified as an intestinal FXR antagonist, we next evaluated its therapeutic effects on MASH. To this end, mice were fed with an MCD diet and orally administered with DC646, GUDCA (an FXR antagonist), or OCA (an FXR agonist) (Fig. 5d). As expected, DC646 selectively inhibited intestinal FXR signaling in MASH mice, without affecting hepatic FXR signaling (Fig. 5e and f). DC646 treatment significantly lowered the serum alanine aminotransferase (ALT) level to a similar extent as GUDCA, whereas OCA cannot improve the serum transferase level in the MCD diet-induced MASH model (Fig. 5g). In addition, the hepatic triglyceride (TG) levels were markedly reduced in DC646-treated mice, which was superior to the lipid-lowering effect of GUDCA and OCA (Fig. 5h). Consistently, hematoxylin and eosin (H&E) staining and Oil Red O staining displayed fewer lipid droplets in the DC646-treated group than the vehicle-, GUDCA-, and OCA-treated groups (Fig. 5i and j). Additionally, F4/80 staining showed that DC646 had good efficacy in reducing macrophage infiltration in the liver, indicating alleviation of hepatic inflammation (Fig. 5i and k). Furthermore, non-alcoholic fatty liver disease (NAFLD) activity scoring (NAS) demonstrated the effectiveness of DC646 in alleviating MASH pathologies, including steatosis, ballooning, and inflammation, which was superior to that of the GUDCA-treated and OCA-treated groups (Fig. 5l). The expression levels of genes involved in liver lipid metabolism, inflammation, and fibrosis revealed that GUDCA only had minor effect on reducing hepatic lipid accumulation, but significantly decreased the mRNA levels of inflammation-related genes including tumor necrosis factor-α (*Tnfa*), C-C motif chemokine ligand 2 (*Ccl2*), C-C motif chemokine ligand 3 (*Ccl3*), interleukin-1β (*Il1b*), and nucleotide-binding oligomerization domain-like receptor protein 3 (*Nlrp3)*, as well as fibrosis-related gene collagen, type IV, alpha 1 (*Col4a1*) (Fig. 5m–o). OCA markedly downregulated the mRNA levels of lipid metabolism-related genes including the elongation of very long-chain fatty acid elongase 6 (*Elovl6*), acyl-coenzyme A (CoA) synthetase long-chain family member 1 (*Acsl1*), stearoyl-CoA desaturase 1 (*Scd1*), fatty acid binding protein 1 (*Fabp1*), cytochrome P450, family 4, subfamily a, polypeptide 10 (*Cyp4a10*), and apolipoprotein E (*Apoe*), as well as fibrosis-related genes collagen, type IV, alpha 2 (*Col4a2*) and collagen, type VII, alpha 1 (*Col12a1*) (Fig. 5m−o). DC646, as our potential candidate for MASH, was found to reduce the mRNA levels of lipid metabolism-related genes including *Acsl1*, CD36 molecule (*Cd36*), *Fabp1*, *Cyp4a10*, carnitine palmitoyltransferase 1b (*Cpt1b*), and *Apoe* (Fig. 5m). The notable reduction was also observed in the mRNA levels of inflammation-related genes including *Tnfa*, plasminogen activator inhibitor 1 (*Pai1*), *Ccl2*, *Ccl3*, *Il1b*, *Nlrp3*, and PYD and CARD domain containing (*Pycard*), as well as fibrosis-related genes collagen, type V, alpha 2 (*Col5a2*) and *Col12a1* (Fig. 5n and o), indicating a better efficacy in alleviating lipid metabolism, inflammation, and fibrosis compared to positive controls, GUDCA and OCA. These results suggest that DC646 is promising in MASH treatment by directly inhibiting intestinal FXR with better anti-MASH activity than the endogenous intestinal FXR antagonist GUDCA and the FXR agonist OCA.

**Figure 6:**
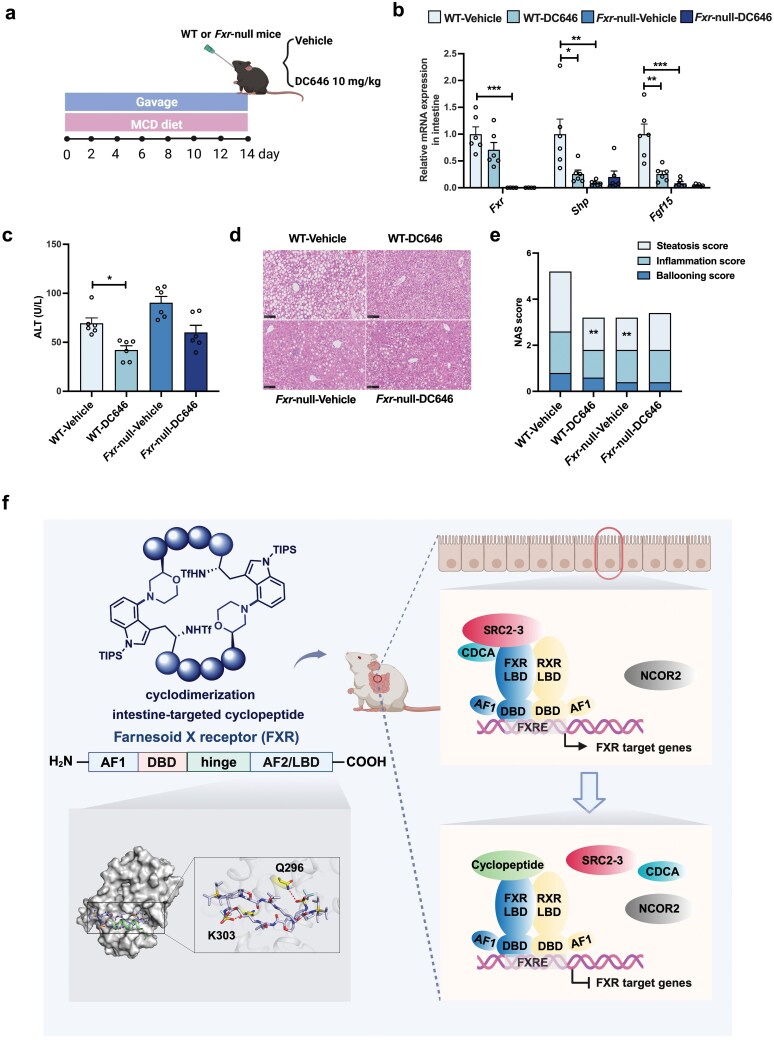
MASH-improving effect of DC646 depends on FXR. (a) Strategy of animal experiments. *Fxr*-null mice have been fed with an MCD to induce MASH for 2 weeks, and been orally administered with DC646. (b) Relative mRNA expression of FXR target genes in the intestine (*n* = 4–6 in each group, the Ct value of *Fxr* in two mice of *Fxr*-null group is not detected). (c) ALT levels. (d) Representative H&E images. (e) NAS score of each group. *n* = 6 in each group. (f) Illustration summary. The figure was created with BioRender.com. Data are presented as mean ± SEM and analyzed by one-way ANOVA with Dunnett’s *post hoc* test for (b), (c), and (e). ^*^P ≤ 0.05, ^**^P ≤ 0.01, and ^***^P ≤ 0.001.

FXR is mainly distributed in the liver and intestine. The aforementioned findings suggest that DC646 predominantly targets intestinal FXR, as indicated by its specific distribution and target selectivity in the intestine (Fig. 5; Supplementary Table S3). To confirm the role of FXR in the observed effects of DC646 on MASH, *Fxr*-null mice were utilized. *Fxr*-null mice and wild-type (WT) mice were fed with an MCD diet and orally administrated with vehicle or DC646 (Fig. 6a). Real-time quantitative PCR (RT-qPCR) analysis revealed that the mRNA expression of *Fxr* was completely absent in vehicle-treated *Fxr*-null mice compared with vehicle-treated WT mice (Fig. 6b). Notably, DC646 treatment significantly downregulated the mRNA levels of the FXR target genes in the intestine, including *Shp* and *Fgf15*, in WT mice but not in *Fxr*-null mice. Additionally, the decrease in ALT as well as the antisteatotic effect observed in WT mice treated with DC646 was abolished in *Fxr*-null mice (Fig. 6c and d). Consistently, the NAS score demonstrated that DC646 effectively reduced steatosis in WT mice but not in *Fxr*-null mice (Fig. 6e). To further confirm the role of intestinal FXR in the obvious effects of DC646 on MASH, *Fxr*^△IE^ mice were fed with a Gubra-Amylin NASH (GAN) diet for 20 weeks and orally administered with vehicle or 10 mg/kg DC646 daily from the 12th week for 8 weeks (Supplementary Fig. S8a). As expected, there was no difference in the mRNA levels of genes in the intestinal FXR signaling (Supplementary Fig. S8b), indicating that DC646 exerted no effect on antagonizing intestinal *Fxr* after intestinal *Fxr* was knocked out. DC646 administration did not reduce the liver/body weight ratio, serum ALT, serum aspartate aminotransferase (AST), liver TG, and liver total cholesterol (TC), as well as MASH pathologies indicated by NAS score in *Fxr*^△IE^ mice (Supplementary Fig. S8c–i). Together, these results demonstrate that DC646 exerts MASH alleviation effect through intestinal FXR.

Several studies have shown that ceramides contribute to the progression of MASH by exacerbating inflammation-induced cellular damage, worsening insulin resistance, and promoting mitochondrial dysfunction and oxidative stress. The inhibition of intestinal FXR facilitates a MASH-alleviating effect by decreasing ceramide synthesis [6, 7]. To delve deeper into the mechanisms behind the MASH improvement effect observed with DC646, 10 and 30 mg/kg DC646 were orally administered to high-fat diet (HFD)-fed mice. DC646 treatment repressed FXR signaling in the ileum in a dose-dependent manner, as revealed by a decrease in the mRNA levels of *Shp* and *Fgf15* (Supplementary Fig. S9a). Lipidomics analysis of ileum metabolites revealed that DC646 reduced ceramide levels in the ileum compared to control groups (Supplementary Fig. S9b). Additionally, DC646 notably decreased the mRNA levels of genes related to ceramide synthesis in the ileum including serine palmitoyl transferase, long chain base subunit 2 (*Sptlc2*), ceramide synthase *2* (*Cers2*), sphingomyelin phosphodiesterase (*Smpd*) *1*, *Smpd3*, and *Smpd4* (Supplementary Fig. S9c). Oral treatment with DC646 also markedly increased serum GLP-1 levels (Supplementary Fig. S9d), which has been reported to be effective on improving MASH through glucose control [30]. These findings suggest that inhibiting intestinal FXR reduces the synthesis and production of intestine-derived ceramides, thereby contributing to the beneficial effects of DC646 on metabolic dysfunction-associated fatty liver disease (MAFLD).

To determine the safety of DC646, a subacute toxicity test with the oral injection of vehicle, 20 mg/kg, 100 mg/kg, or 200 mg/kg DC646 to C57BL/6 male mice was carried out for 7 days (Supplementary Fig. S10a). During the week, the body weight and food/water intake were monitored every day. It was demonstrated that administration of 20 and 100 mg/kg DC646 did not affect the body weight and food/water intake (Supplementary Fig. S10b−d), while administration of 200 mg/kg DC646 mildly decreased the food/water intake with normal body weight (Supplementary Fig. S10b−d). There was no difference in organ morphology, small intestine, and colon length, as well as organ weight including liver, kidneys, brain, heart, lungs, spleen, testicle, and epididymis in the four groups (Supplementary Fig. S10e−g). Additionally, DC646 did not cause liver injury indicated by serum ALT and AST (Supplementary Fig. S10h and i). The cellular structures and morphologies of the heart, liver, spleen, lungs, and kidneys were also not influenced by DC646 at the three dosages (Supplementary Fig. S10j). Although a high dose of DC646 reduced appetite, it did not cause body weight loss or obvious organ toxicity. Collectively, DC646 is a compound with good tolerance up to the highest dosage (200 mg/kg) tested in this study, which is more than 20-folds over its minimum effective dose, suggesting a good safety profile.

Cyclopeptide DC646 serves as a PPI inhibitor to mediate intestine-targeted FXR antagonism by interacting with FXR-LBD and disrupting the interaction between FXR-LBD and coactivator. This mechanism is distinct from those of known FXR antagonists. DC646 also shows potentials for the therapeutics of MASH, which is through intestinal FXR-ceramide axis and GLP-1 production (Fig. 6f).

## Discussion

NRs have emerged as significant targets for drug development due to their regulatory role in various physiological and pathological processes. Given the pivotal role of FXR in these processes, ligands that modulate FXR activity have shown promise as therapeutic agents for a range of diseases. While several FXR agonists for MASH treatment are currently undergoing clinical trials, challenges related to dyslipidemia and pruritus continue to impede the development of FXR agonists. Recently, attention has shifted towards the development of intestine-targeted FXR antagonists, which exhibit physiological activities such as weight control, alleviation of insulin resistance, and improvement of MASH. However, the FXR antagonists reported so far target the LBD, specifically the LBP, thereby indirectly modulating FXR activity by preventing the binding of primary bile acids to FXR-LBP and disrupting the interaction and communication between FXR and coactivators. In this study, we conducted screening and optimization of our cyclopeptide library and discovered, for the first time, intestine-targeted FXR antagonists that directly affect the interaction between FXR and coactivators.

FXR activates gene transcription by recruiting coactivators to initiate the transcription of its target genes. When bound to secondary bile acids such as GUDCA, the structure of FXR-LBP transitions into an antagonistic conformation, which is essential for the functioning of AF2. The FXR bound to GUDCA interacts with various coactivators in the cellular environment through the AF2 site on the surface of FXR, thereby regulating the transcription of downstream genes. In order to screen for compounds that directly affect the AF2 site, blocking the PPIs between FXR-LBD and coactivators, we calculated the size of FXR-bound coactivators in the published experimental complex structures and the size of compounds in our cyclopeptide library. Through activity screening, we found that the cyclopeptide DC644, with a size larger than that of coactivators, exhibited promising FXR antagonistic activity. Molecular docking simulations confirmed that DC644 indeed interacted with the surface of FXR-LBD, occupying the coactivator-binding pocket. These results encouraged us to further explore the discovery of cyclopeptides with stronger FXR antagonistic activity. Through structural optimization of the cyclopeptide size and side chains, we identified the cyclopeptide DC646, which strongly inhibited FXR activation by more than 70%. Furthermore, in the presence of the endogenous agonist CDCA, DC646 inhibited the recruitment of the coactivator SRC2-3, with an IC_50_ value of 8.86 μmol/L. In the presence of Ivermectin, DC646 not only affected the recruitment of coactivators but also hindered the recruitment of corepressors, indicating that DC646 functions as a PPI inhibitor, disrupting the interaction between FXR-LBD and cofactors, thereby regulating FXR activity. Molecular docking simulations and site-directed mutagenesis of key amino acid residues Q296 and K303 further confirmed that the binding site of DC646 was the AF2 site of FXR-LBD. To validate this finding, we conducted an AlphaScreen assay with different administration sequences of CDCA and DC646. The results showed that DC646 exhibited antagonistic activity when combined with CDCA, with an IC_50_ value of 12.19 μmol/L. Adding DC646 before CDCA completely nullified the activating effect of CDCA, while adding DC646 after CDCA did not exhibit antagonistic activity, indicating that DC646 antagonized FXR by binding to the surface of FXR-LBD and inhibiting the binding of coactivators. Additionally, based on the structure of DC646, we designed and synthesized a probe cyclopeptide, BIOTIN-DC646, which contained a biotin moiety. Through co-immunoprecipitation assay, we further confirmed the inhibitory effect of DC646 on PPIs, consistent with the results of the AlphaScreen assay.

FXR is primarily expressed in the liver and intestine. FXR in the liver may act as a tumor suppressor involved in the progression of HCC, with its expression negatively correlated with various malignant clinical and pathological features of HCC. Furthermore, the antagonistic effect of FXR in the liver is associated with liver injury and bile stasis. Therefore, it is important to avoid FXR antagonism in the liver during the development of intestine-targeted FXR antagonists. In the experiments conducted on intestinal organoids, we confirmed that cyclopeptide DC646 effectively reversed the GW4064-induced mRNA expression of FXR target gene *Shp* in an FXR-dependent manner. In both intestinal organoids and target selectivity experiments, we also found that DC646 did not significantly affect the expression of other target genes of NRs, such as *Pxr*, *Vdr*, and *Nr1i3*, nor did it exhibit significant transactivation or transrepression of PPARα, PPARδ, and PPARγ, indicating a higher target selectivity. Further *in vivo* experiments in mice demonstrated that DC646 induced the mRNA expression of FXR target genes *Shp* or *Fgf15*, without affecting the expression of genes involved in hepatic FXR signaling. Moreover, tissue distribution monitoring *in vivo* showed that DC646 was retained in the intestine and not present in the liver. The subacute toxicity study also demonstrated that DC646 can tolerate doses 20-fold higher than the minimum effective dose, with no significant organ toxicity, exhibiting a good safety profile. These results confirm that DC646 is a highly selective intestine-targeted FXR antagonist.

MASH is a complex liver dysfunction associated with insulin resistance, mitochondrial dysfunction, and lipid accumulation. In MAFLD/MASH, systemic FXR activation enhances biliary secretion and reduces bile acid synthesis and uptake, maintaining bile acid homeostasis. Hepatic FXR activation inhibits *de novo* lipogenesis and reduces steatosis [31]. Intestinal FXR activation attenuates bile acid synthesis and preserves intestinal barrier integrity [32]. However, intestinal FXR antagonism reduces lipid accumulation by inhibiting ceramide-sterol regulatory element-binding protein 1c (SREBP1c) axis-mediated lipogenesis [6, 7, 33]. Under obesity conditions, intestinal FXR is persistently activated, and its inhibition enhances metabolic function [34]. In MAFLD/MASH, the activity of intestinal FXR may vary a lot, which depends on metabolic status. Chronic activation may impair metabolic function, while antagonism can inhibit steatosis and reactivate hepatic FXR. Resmetirom, a thyroid hormone receptor beta (THR-β) agonist, was recently approved by the United States Food and Drug Administration (FDA) for MASH treatment, but only for patients with F2/F3 fibrosis [35]. This highlights the need for more therapies. Increasing evidence suggests that targeting the “gut-liver axis” is a novel strategy for treating MASH [36, 37]. The discovery of GlyMCA has overcome the issue of easy metabolism by bacterial bile salt hydrolase, which affects endogenous intestinal FXR antagonists, and has shown great potency in improving obesity and insulin resistance caused by lipid accumulation [5]. Recently, the anti-MASH effect of GlyMCA has been uncovered, which occurs through the inhibition of ceramide synthesis, rather than weight loss, as evidenced by reduced lipotoxicity, inflammation, and fibrosis [7]. Both THR-β agonists and intestinal FXR inhibition share overlapping mechanisms, including effects on bile acid and glucose metabolism via GLP-1 [38, 39]. GLP-1 receptor (GLP-1R) is also clinically effective for MASH [40]. This renders the inhibition of intestinal FXR as a highly promising therapeutic strategy for the treatment of MASH. DC646 exhibits significant improvements in steatosis, as well as inflammation and fibrosis to a lesser extent. In our study, we used an MCD diet to induce MASH, which prominently manifests as hepatic steatosis, accompanied by inflammation and mild fibrosis [41]. This may explain the superior efficacy of DC646 in ameliorating steatosis under the MCD model compared to GUDCA and OCA. The discovery of DC646 provides a new approach to developing an anti-MASH drug. Considering its potent lipid-lowering effects, the potential of DC646 as a therapeutic option for other metabolic diseases, such as obesity and diabetes, can be further explored. Additionally, to further confirm the role of intestinal FXR in the effects of DC646 on MASH, we conducted *in vivo* efficacy evaluations in *Fxr*-deficient mice and found that the observed reductions in ALT levels and anti-steatosis effects in WT mice treated with DC646 were abolished in *Fxr*-deficient mice, demonstrating that the beneficial effects of DC646 on steatosis and MASH development are mediated through its specific targeting of intestinal FXR. In HFD-fed mouse models, DC646 can inhibit intestinal FXR, reduce the synthesis and production of enteric-derived ceramides, and induce the increase of GLP-1 expression, which further elucidated the beneficial mechanism of DC646 on MAFLD.

In summary, we have developed novel cyclopeptide antagonists targeting intestinal FXR, characterized by an excellent safety profile, for mechanistic exploration and the treatment of MASH. This work not only reveals the potential of intestine-targeted FXR antagonists as a therapeutic strategy for MASH but also uncovers a new binding pocket for FXR antagonists.

### Limitations of the study

It has been reported that deficiency of intestinal FXR is associated with increased serum levels of GLP-1 [42, 43]; however, activation of intestinal FXR can also stimulate GLP-1 release through the activation of Takeda G protein-coupled receptor 5 (TGR5) signaling [44, 45]. The specific role of intestinal FXR in stimulating GLP-1 remains to be fully elucidated in this study. Additionally, our study did not investigate the effects of DC646 on female mice. Given the sex-specific differences observed in the pathogenesis of MASH and dietary responses, further extensive research and mechanistic exploration are necessary.

## Materials and methods

### Materials

All the reagents are of analytical grade. Dimethyl sulfoxide (DMSO) (Cat# 472301), CDCA (Cat# C9377), and TCA sodium salt hydrate (Cat# T4009) were obtained from Sigma. GUDCA (Cat# HY-N1424), GlyMCA (Cat# HY-114392), and sitagliptin (Cat# HY-13749) were sourced from MedChemExpress. GW4064 (Cat# S2782) was acquired from Selleck Co.; OCA (Cat# 459789-99-2) was obtained from Shanghai MissYou Chemical Co., Ltd. Neomycin sulfate (Cat# MB1716), streptomycin sulfate (Cat# MB1275), and bacitracin (Cat# MB1374) were obtained from Dalian Meilun Biological Technology Co., Ltd. The methionine- and choline-sufficient (MCS) diet (Cat# A02082003B), MCD diet (Cat# A02082002B), HFD (Cat# D12492), and GAN diet (Cat# D09100310) were purchased from Research Diets, Inc. TRIeasy total RNA extraction reagent (Cat# 10606ES60), qPCR SYBR Green Master Mix (Cat# 11184ES08), and HiScript III RT SuperMix for qPCR (Cat# R323-01) were sourced from Yeasen Biotechnology (Shanghai) Co., Ltd. Biotin-labeled SRC2-3 peptide (Bio-SRC2-3, Bio-QEPVSPKKKENALLRYLLDKDD TKD) and Biotin-labeled NCoR2 peptide (Biotin-NCoR2, Bio-GHSFADPASNLGLEDIIRK ALMGSF) were synthesized by Shanghai Shenggong Co. Matrigel (Cat# 356255), Dulbecco’s modified Eagle’s medium (DMEM) (Cat# C11995500CP), and fetal bovine serum (FBS) (Cat# 10091148) were obtained from Gibco. Human embryonic kidney (HEK293T) cells were obtained from the American Type Culture Collection (ATCC) cell bank (Cat# CRL-3216). IntestiCult Organoid Growth Medium with Supplements 1 and 2 (Cat# 06005) was obtained from STEMCELL Ltd. FIREFLYGLO Luciferase Assay System (Cat# MA0519) was obtained from Dalian Meilun Biological Technology Co., Ltd. CellTiter-Blue® Cell Viability Assay (G8082) was obtained from Promega Corporation. FXR coactivator recruitment assay kit (Cat# A15140) was obtained from Thermo Fisher Scientific. The histidine detection kit for AlphaScreen assay (Cat# 6760619C) was purchased from PerkinElmer Life Sciences. TG assay kit (Cat# A110-2-1), TC assay kit (Cat# A111-1-1), ALT assay kit (Cat# C009-3-1), and AST assay kit (Cat# C010-2-1) were obtained from Nanjing Jiancheng Bioengineering Institute. The mutation assay was carried out by QuickMutation™ Site-Directed Mutagenesis Kit (Cat# D0206M) from Beyotime Biotechnology. Active GLP-1 kits (Cat# EGLP-35K) were obtained from Millipore Corporation.

### Animal studies

All the animal protocols were conducted according to the institutional ethical guidelines on animal care and were approved by the Institute Animal Care and Use Committees at Shanghai Institute of Materia Medica, Chinese Academy of Sciences. All mice were housed in a standard specific-pathogen-free environment. Mice were maintained under a standard 12-h light/12-h dark cycle with water and food provided *ad libitum*. Male C57BL/6 J mice (8–10 weeks old, 23–25 g) were obtained from Beijing Huafukang Bioscience Co. Inc. *Fxr*-null mice were purchased from Shanghai Model Organisms Center, Inc.

For the *in vivo* intestinal FXR antagonism experiment, after a 3-day treatment with an antibiotic cocktail (1 mg/mL neomycin, 1 mg/mL streptomycin, and 1 mg/mL bacitracin) in drinking water, 8- to 10-week-old male C57BL/6 J mice were orally administered with vehicle or TCA (400 mg/kg/day), TCA combined with GUDCA (50 mg/kg), or TCA combined with DC646 (20 mg/kg) once a day. The serum, liver, and intestine were collected 2 h after the third dosing.

For the long-term MASH model, 8- to 10-week-old male C57BL/6 J mice were fed with an MCS diet or MCD diet for 2 weeks, before the MCD models were administered with vehicle or GUDCA (50 mg/kg), OCA (30 mg/kg), and DC646 (10 mg/kg) for 4 weeks. The serum, liver, and intestine were collected 2 h after the final dosing. Male *Fxr*^△IE^ mice (8–10 weeks old) were fed with a GAN diet for 20 weeks and administered with vehicle or DC646 (10 mg/kg) from the 12th week for 8 weeks. The serum, liver, and intestine were collected 2 h after the final dosing.

For the short-term MASH model, 8- to 10-week-old male C57BL/6 J or *Fxr*-null mice were fed with an MCD diet, combined with oral administration of vehicle or DC646 (10 mg/kg) for 2 weeks. The serum, liver, and intestine were collected 2 h after the final dosing.

For the subacute toxicity test of DC646, oral injection of vehicle, 20 mg/kg, 100 mg/kg, or 200 mg/kg DC646 to C57BL/6 male mice was carried out for 7 days. During the week, the body weight and food/water intake were monitored every day. The serum, liver, kidneys, brain, heart, lungs, spleen, testicle, and epididymis were weighed and collected 2 h after the final dosing.

### Serum GLP-1 detection

Mice were fasted for 6 h and gavaged with a single dose of 25 mg/kg sitagliptin, followed by 2 g/kg glucose 45 min later [43]. Blood samples were collected 15 min after glucose administration, and GLP-1 was measured using active GLP-1 kits.

### Cell culture

HEK293T cells were used for the luciferase report assays and were cultured in DMEM with 10% FBS. The cells were incubated in DMEM media with 1% FBS for 4–6 h and then exposed to the indicated concentrations of compounds for 24 h.

### Luciferase reporter assay

For the FXR antagonism experiment, HEK293T cells were seeded in 96-well plates and transfected with pCMV-Script-hFXR and pGL4.11-hSHP-luciferase at a density of 3 × 10^4^ cells/well, before culturing overnight. The compounds at indicated concentrations were added to the cells and incubated at 37°C for 1 h, before stimulating with 1% DMSO (negative control) and 5 μmol/L GW4064 (positive control) for 24 h. For the FXR agonism experiment, HEK293T cells were seeded in 96-well plates and transfected with pCMV-Script-hFXR and pGL4.11-hSHP-luciferase at a density of 3 × 10^4^ cells/well, before culturing overnight. Then compounds at indicated concentrations were added to the cells and incubated at 37°C for 24 h. For the mutation experiment, HEK293T cells were seeded in 96-well plates and transfected with pcDNA3.1-hFXR or pcDNA3.1-hFXR-mutation and pGL4.11-hSHP-luciferase at a density of 3 × 10^4^ cells/well, before culturing overnight. The mutation assay was carried out by the guidance of QuickMutation™ Site-Directed Mutagenesis Kit. The primer sequences are as followings: Q296 forward: 5′-GGCAACCAATCATGTAGCGGTTCTTGTAGAATTCAC-3′; Q296 reverse: 5′-GTGAATTCTACAAGAACCGCTACATGATTGGTTGCC-3′; K303 forward: 5′-GATTGCTTTGCTGGAAGGGTCTGCGGTTGAAGC-3′; K303 reverse: 5′-GCTTCAACCGCAGACCCTTCCAGCAAAGCAATC-3′.

The luciferase activities and cell viabilities were detected by the FIREFLYGLO Luciferase Assay System and Cell Titer-Blue® Cell Viability Assay according to the manufacturer’s instructions. Firefly luciferase activities and cell viabilities were measured using Spectra Max M5e (Molecular Devices).

### Intestinal crypt isolation and culture

Intestinal crypts were isolated from 6- to 8-week-old C57BL/6 mice and *Fxr*^*△IE*^ mice as previously described with minor revision [46]. Briefly, the abdomen of the mouse was dissected, and the small intestine was isolated with fats removed. The isolated intestine was flushed with cold PBS to remove the luminal contents. Then, the intestine was opened longitudinally and washed three times with PBS gently. The tissue was cut into small fragments (2 mm^2^) and further washed three times with cold PBS gently, followed by incubation with 5 mmol/L EDTA-PBS at 4°C. After 40 min, the fragments were vortexed vigorously, and then the suspension was filtered through a 70-mm filter and centrifuged at 50 *g* for 10 min. The crypts were counted and plated with Matrigel (1:1, 25 μL) and 500 μL IntestiCult Organoid Growth Medium with Supplements 1 and 2. The isolated crypts were cultured for 3–5 days and treated with indicated chemicals as described.

### AlphaScreen assay

An AlphaScreen technology-based assay was performed according to the manufacturer’s instructions with a minor revision. Briefly, the experiment was conducted with compounds at the indicated concentrations, 100 nmol/L 6*His-hFXRα-LBD and 100 nmol/L biotinylated co-factor peptide in the buffer containing 50 mmol/L MOPS, 50 μmol/L CHAPS, 50 mmol/L NaF, and 0.1 mg/mL BSA, pH 7.4. The mixture was incubated with 50 μg/mL nickel acceptor beads and 50 μg/mL streptavidin donor beads in a 384-well plate under green light in the dark at room temperature, before conducting fluorescence measurement with excitation at 680 nm and emission at 550 nm on an Envision microplate analyzer (PE 2102-8340, PerkinElmer Life Sciences). The binding signals were detected in groups with (i) GUDCA at 50 μmol/L, GlyMCA at 50 μmol/L, and DC644, SJN10, SJN18, HT14, HT15, and HT18-2 at 10 μmol/L combined with CDCA; (ii) various concentrations of CDCA, DC646, and DC646 combined with CDCA (50 μmol/L); or (iii) Ivermectin, DC646 combined with Ivermectin (5 μmol/L), and 6*his-biotinylated peptide as a positive control and DMSO as a negative control.

### Immunoprecipitation

For the co-immunoprecipitation of Biotin-DC646 and FXR-LBD, 50 μmol/L Biotin-DC646 were incubated with 50 μmol/L purified FXR-LBD at room temperature for 2 h at indicated conditions. These mixtures were then immunoprecipitated with beads which had been incubated with anti-His antibody (12698S, Cell Signaling Technology) at room temperature for 1 h. Immunoblotting was performed using the anti-BIOTIN antibody (sc-101339, Santa Cruz Biotechnology).

### RT-qPCR analysis

The intestine and liver were frozen in liquid nitrogen and stored at −80°C. RNA was extracted, and complementary DNA was synthesized from 1 μg total RNA. RT-qPCR was performed on a Bio-Rad CFX384 Touch detection system (Bio-Rad). The primer sequences for RT-qPCR are shown in Supplementary Table S5. RNA levels were analyzed by 2-^ΔΔ*Ct*^, normalized to 18S ribosomal RNA and expressed as fold change relative to the control group.

### Biochemistry analysis

Hepatic TG and TC, and plasma ALT and AST levels were measured using commercial kits from Nanjing Jiancheng Bioengineering Institute according to the manufacturer’s instructions. To measure hepatic TG contents, liver tissues (~10 mg) were homogenized in 100 μL 1% TritonX-100 and centrifuged at 3000 rpm for 10 min. Supernatants were collected, and 2 μL samples were incubated with 200 μL reaction buffer at 37°C for 10 min. Hepatic TG or TC levels were read at 510 or 500 nm using a microplate reader (Versamax, Molecular Devices) and normalized using the protein concentration. To measure plasma ALT and AST levels, reaction buffer 1 and reaction buffer 2 were mixed at a ratio of 1:1, before adding 2 μL plasma sample and 200 μL mixed reaction buffer in a 96-well plate. The data were acquired using the kinetic mode with an interval of 30 s and 31 total reads at 340 nm using a microplate reader (Versamax, Molecular Devices).

### Histological analysis

Tissue specimens were fixed in 10% formalin for 12–24 h before being dehydrated and paraffin-embedded. The sections were stained with H&E. The NAS was calculated as a summary score including individual components of steatosis (0–3), lobular inflammation (0–3), and hepatocyte ballooning (0–2) for each liver section by a blinded liver pathologist, and the results were reviewed by a blinded hepatologist according to a previous protocol [47]. To visualize liver fibrosis, paraffin-embedded tissue samples were stained with Sirius Red to characterize the collagen deposition. To visualize lipid accumulation and inflammation in the liver, frozen liver sections were stained with Oil Red O and F4/80, and images were acquired using a Leica DM6B laser microdissection system. At least three discontinuous liver sections were evaluated for each mouse.

### Ceramide analysis

For ileal lipidomics, samples were homogenized, extracted with chloroform, and prepared for analysis. Separation was achieved using an Acquity UPLC CSH C18 column, and analysis was conducted via liquid chromatography electrospray ionization tandem mass spectrometry (LC-ESI-MS/MS). Mobile phases were comprised with acetonitrile–water and isopropanol/acetonitrile solutions, both containing 10 mmol/L ammonium acetate and 0.1% formic acid [7].

### Pharmacokinetic study

Male ICR mice (6–8 weeks old, 18–22 g, Shanghai Lingchang BioTech Co., Ltd., China) were used in the pharmacokinetic study. The mice were administered with a single dose of cyclopeptide DC646 in 5% DMSO and 95% hydroxypropyl methylcellulose solution (0.5% hydroxypropyl methylcellulose in deionized water in weight ratio) (volume ratio) by oral gavage (20 mg/kg). Before the test, the mice were fasted for 12 h and were fed regularly 2 h after administration. Subsequently, 50 μL of blood was collected from the portal vein at 1, 4, 8, and 24 h in an EDTA-K2 nebulization tube. Samples were centrifuged at 12,000 rpm for 2 min to separate the plasma, which was then stored at −20°C to determine the drug concentration in the blood. After sacrifice, the livers and intestines were collected, washed with ice-cold saline, and stored at −20°C before compound measurement.

### Quantification and statistical analysis

Experimental data are presented as the mean ± SEM. Statistical analysis was performed using GraphPad Prism 8.0.1 (GraphPad Software Inc., San Diego, CA, USA). Statistical significance was determined using unpaired two-tailed Student’s *t*-test and multiple *t*-test between two groups, and using one-way analysis of variance (ANOVA) with Dunnett’s *post hoc* test for multi-group comparisons. The *P* values are noted in each figure legend and indicated as ^*^P < 0.05, ^**^P < 0.01, and ^***^P < 0.001. The statistical details for each experiment are indicated in the figure legend.

### Molecular docking

To predict the inhibitor-binding models of FXR, we employed the X-ray structures of human FXR in complex with ligands (PDB codes: 1OSH, 3BEJ, 3DCT, 3FLI, 3L1B, 3RUT, 3RVF, 4WVD, 5IAW, 5ICK, 5WZX, 5Z12, 6HL0, 6HL1, 7D42, and 7VUE) [28, 29, 48–58]. Fpocket [59] is used to detect the LBPs on these structures. A compound was docked to FXR using Autodock4. Searches were performed using Lamarckian Genetic Algorithm with default settings. The docking model with the lowest ligand-binding affinity score was selected for analysis.

## Supplementary Material

loaf004_suppl_Supplementary_Material

## Data Availability

Deposition Numbers 2172935 (for DC646) contains the supplementary crystallographic data for this paper. These data are provided free of charge by the joint Cambridge Crystallo-graphic Data Centre and Fachinformationszentrum Karlsruhe Access Structures service. Data supporting the findings of this study are available within the paper and Supplementary Information. No custom computer code is used to generate the results reported in the paper. Correspondence and requests for materials may be addressed to H.L. Source data are provided in this paper.
