# Supplementary material for: Development of cyclopeptide inhibitors specifically disrupting FXR–coactivator interaction in the intestine as a novel therapeutic strategy for MASH
Source: Life Metab. 2025 Feb 8;4(2):loaf004. doi: 10.1093/lifemeta/loaf004 (PMC11992618; doi:10.1093/lifemeta/loaf004)
Supplement: loaf004_suppl_Supplementary_Material [file loaf004_suppl_supplementary_material.docx]

Supplementary Materials for

Development of cyclopeptide inhibitors specifically disrupting FXR-coactivator interaction in the intestine as a novel therapeutic strategy for MASH

Yazhou Li^1,2,‡^, Tingying Jiao^2,3,‡^, Xi Cheng^1,2,‡^, Lu Liu^2^, Mengjiao Zhang^4^, Jian Li^2^, Jue Wang^5^, Shulei Hu^2^, Cuina Li^2^, Tao Yu^2^, Yameng Liu^2^, Yangtai Li^6^, Yu Zhang^2,7^, Chuying Sun^4^, Jina Sun^2,7^, Jiang Wang^2,7,*^, Cen Xie^2,4,*^, Hong Liu^1,2,4,*^

Correspondence to: [hliu@simm.ac.cn](mailto:hliu@simm.ac.cn); xiecen@simm.ac.cn; jwang@lglab.ac.cn

This PDF file includes:

Supplementary Text

Supplementary Figures S1−S10

Supplementary Tables S1−S5

Supplementary Text

1. General information

Unless specified otherwise, the reagents were purchased from commercial sources and used without further purification. Analytical thin-layer chromatography (TLC) was performed on HSGF 254 with a 0.2−0.3 mm thickness. Column chromatography was performed on silica gel (300−400 mesh) using petroleum ether (PE)/ethyl acetate (EA) or dichloromethane (DCM)/methanol (MeOH). For reactions that require heating, the heat source was an oil bath. All products were characterized by their nuclear magnetic resonance (NMR), low-resolution mass spectrometry (LRMS), and high-resolution mass spectrometry (HRMS) spectra. Proton coupling patterns were described as singlet (s), doublet (d), triplet (t), quartet (q), multiplet (m), doublet of doublets (dd), doublet of triplets (dt), triplet of doublets (td), and doublet of doublet of doublets (ddd). The chemical shifts were reported in parts per million (ppm, δ) downfield from tetramethylsilane (TMS). HRMS were measured on a Micromass Ultra Q-TOF spectrometer. Crystal structure determination were performed on a Bruker D8 VENTURE diffractometer.

2. Chemical synthesis

General procedures for the preparation of substrates

According to the general procedure (Supplementary Figure S1), cyclopeptide substrates 1a and 1d were prepared. Triethylamine (Et_3_N, 1.0 mL, 7.5 mmol) and trifluoromethanesulfonic anhydride (0.9 mL, 5.5 mmol) **were slowly added to a stirred solution of compound a (5.0 mmol) in DCM (30 mL) at −78 °C**. After stirring at −78 °C for 4 h, the mixture was quenched by the addition of saturated NaHCO_3_ and then extracted with CH_2_Cl_2_ for three times. The organic layer was combined, dried over anhydrous Na_2_SO_4_, and concentrated under vacuum. Purification by column chromatography on silica gel (PE/EA) yielded product b.

**n-butyllithium (**n-BuLi**,** 4.1 mmol) **was slowly added to a solution of compound b (2.0 mmol) in anhydrous tetrahydrofuran (THF, 20 mL) at −78 °C**. After stirring at −78°C for 10 min, **triisopropylsilyl chloride (**TIPSCl**,** 0.8 mL, 4.0 mmol) was added dropwise. After stirring at −78 °C for 3 h, the reaction was quenched by adding aqueous NH_4_Cl solution. The mixture was extracted with ethyl acetate (EtOAc) for three times. The organic layer was combined, dried over anhydrous Na_2_SO_4_, and concentrated under vacuum. The resulting residue was purified by column chromatography on silica gel to yield product c.

Lithium hydroxide (LiOH·H_2_O, 10.0 mmol) was added to a solution of compound c (5.0 mmol) in THF (5.0 mL) and H_2_O (2.5 mL). After stirring at room temperature for 36 h, the reaction was quenched by adding 1N HCl solution and extracted with EtOAc for three times. The organic layer was combined, dried over anhydrous Na_2_SO_4_, and concentrated under vacuum. The resulting residue was purified by column chromatography on silica gel to yield product d.

The (S)-4-(tert-butoxycarbonyl)morpholine-2-carboxylic acid (e, 1.0 mmol) and 4-methylmorpholine (NMM, 1.7 mmol) were dissolved in dimethyl formamide (DMF) at 0°C. Amino alcohol (1.0 mmol) and 1-hydroxy-7-azabenzotriazole (HOAT 1.2 mmol) were then added to the reaction mixture. 1-ethyl-3-(3-(dimethylamino)propyl)carbodiimide hydrochloride (EDCI, 1.2 mmol) was added in one portion at 0°C. The reaction was performed at 0°C for 1.5 h and then at room temperature for 6 h before quenched by adding H_2_O. The aqueous layer was extracted with EtOAc. The organic phase was washed successively with H_2_O, 0.5 N HCl solution, saturated aqueous NaHCO_3_ solution, and brine before dried over anhydrous Na_2_SO_4_. The organic layer was evaporated to dryness under vacuum. Purification by column chromatography on silica gel (PE/EA) yielded the product f.

EDCI (0.3 mmol), 4-dimethylaminopyridine (DMAP, 0.1 mmol), and compound c (0.2 mmol) were added to a solution of compound f (0.2 mmol) in 10 mL anhydrous DCM. The resulting solution was stirred at room temperature overnight. Then, the reaction was quenched with water and extracted with DCM. The combined organic layer was washed with brine, dried over Na_2_SO_4_, and concentrated in vacuo. The residue was subjected to column chromatography, and product g was obtained.

HCl (4 mol/L)/1,4-dioxane (8.0 equiv.) were added to a solution of compound g (1.0 equiv.) in anhydrous dichloromethane (0.5 mol/L). After stirring at room temperature for 1 h, the reaction solution was concentrated in vacuo to yield product h.

Potassium phosphate dibasic (3.0 equiv.) and amine (h) (1.5 equiv.) were added to a solution of benzoyl peroxide (1.0 equiv.) in DMF (0.4 mol/L) at 0^o^C. After 30 min, the mixture **wa**s allowed to warm to room temperature and stirred for 12 h. The reaction mixture **wa**s monitored by TLC. After disappearance of starting material, the reaction mixture **wa**s quenched with distilled water and extracted with EA (two times). The combined organic layer **wa**s washed with aqueous NaHCO_3_ (two times) and brine (one time), and dried over Na_2_SO_4_. After removal of solvent, the residue **wa**s purified by flash chromatography on silica gel to yield a corresponding desired products 1a or 1b.

Supplementary Figure S1 Synthesis of cyclopeptide substrates 1a and 1b.

According to the general procedure (Supplementary Figure S2), cyclopeptide substrates 1c−1g are prepared.

Supplementary Figure S2 Synthesis of cyclopeptide substrates 1c−1g.

General procedures for the preparation of cyclopeptide compounds DC644−649

1a−1f (80.0 mg), Pd(CH_3_CN)_2_Cl_2_ (20 mol %), AgOAc (2 equiv.), Na_3_PO_4_ (3 equiv.), and DCE (2.5 mL) were added to a 25-mL capped vial. The reaction mixture was stirred at 85^o^C for 24 h. After that, the reaction mixture was filtered through a pad of celite washing with **EtOAc**. The organic layer was washed with saturated NaHCO_3_, dried over Na_2_SO_4_, and concentrated in vacuo. The residue was purified by silica gel chromatography using PE/EA to yield the products DC644−DC649.

Supplementary Figure S3 Synthesis of cyclopeptide compounds DC644−DC649.

General procedures for the preparation of cyclopeptide compound BITION-DC646

Compound 1c (200.0 mg, 0.219 mmol), 1g (220.2 mg, 0.219 mmol), Pd(CH_3_CN)_2_Cl_2_ (22.8 mg, 0.088 mmol), AgOAc (146.7 mg, 0.879 mmol), Na_3_PO_4_ (216.2 mg, 1.320 mmol), and DCE (5.0 mL) were added to a 25-mL capped vial. The reaction mixture was stirred at 85^o^C for 24 h. After that, the reaction mixture was filtered through a pad of celite washing with **EtOAc**. The organic phases were washed with saturated NaHCO_3_, dried over Na_2_SO_4_, and concentrated in vacuo. Purification by column chromatography on silica gel (PE/EA 4:1−2:1) yielded **the product** 1h (91.6 mg, 25%) as white solid.

Compound 1h (90.0 mg, 0.054 mmol) was dissolved in methanol (5 mL) in a 25-mL round bottom flask and Pd/C (10.0 mg) was added. The mixture was stirred under hydrogen at room temperature for 12 h. The solid was filtered through a celite pad and the filtrate was concentrated in vacuo to yield the product 1i in 88 % yield (74.9 mg).

The 1i (60 mg, 0.036 mmol) and NMM (7.28 mg, 0.072 mmol) were dissolved in DMF (5 mL) at 0°C. N-(14-amino-3,6,9,12-tetraoxatetradecyl)-5-((3aS,4S,6aR)-2-oxohexahydro-1H-thieno[3,4-d]imidazol-4-yl)pentanamide (1j, 16.6 mg, 0.036 mmol) and HOAT (5.0 mg, 0.037 mmol) were then added to the reaction mixture. EDCI (8.3 mg, 0.043 mmol) was added in one portion at 0°C. The reaction was performed at 0°C for 1.5 h and then at room temperature for 6 h before quenched by adding H_2_O. The aqueous layer was extracted with EtOAc. The organic phase was washed successively with H_2_O, 0.5 N HCl solution, saturated aqueous NaHCO_3_ solution, and brine before dried over anhydrous Na_2_SO_4_. The organic layer was evaporated to dryness under vacuum. Purification by preparative TLC (DCM/MeOH = 10:1 (v/v)) yielded BIOTIN-DC646 (30.6 mg, 42%) as a white solid.

Supplementary Figure S4 Synthesis of BIOTIN-DC646.

3. Analytical data of substrates and products

(S)-2-((5-(((S)-2-(trifluoromethylsulfonamido)-3-(1-(triisopropylsilyl)-1H-indol-3-yl)propanoyl)oxy)pentyl)carbamoyl)morpholino benzoate (1a)

^1^H NMR (400 MHz, CDCl_3_) δ 7.99 (d, J = 7.4 Hz, 2H), 7.60 – 7.51 (m, 2H), 7.49 – 7.37 (m, 3H), 7.19 – 7.07 (m, 3H), 6.63 (s, 1H), 6.40 (d, J = 3.8 Hz, 1H), 4.51 (dd, J = 14.0, 5.9 Hz, 1H), 4.30 (d, J = 8.1 Hz, 1H), 4.11 – 3.89 (m, 5H), 3.45 (d, J = 6.3 Hz, 1H), 3.35 (d, J = 5.9 Hz, 2H), 3.28 – 3.13 (m, 2H), 2.91 (s, 1H), 2.75 (s, 1H), 1.75 – 1.59 (m, 3H), 1.59 – 1.43 (m, 4H), 1.37 – 1.22 (m, 2H), 1.11 (d, J = 7.5 Hz, 18H). ^13^C NMR (101 MHz, CDCl_3_) δ 170.5, 168.5, 164.5, 141.2, 133.3, 130.6, 130.3, 129.5, 128.9, 128.5, 121.9, 119.9, 119.5 (q, J = 320.8 Hz), 118.3, 114.2, 110.5, 75.1, 66.0, 65.3, 58.7, 57.8, 56.3, 38.6, 29.4, 29.0, 27.8, 23.1, 18.1, 12.8. ^19^F NMR (375 MHz, CDCl_3_) δ -77.6. HR-MS (ESI-TOF) m/z: [M+H]^+^ Calcd for C_38_H_54_F_3_N_4_O_8_SSi 811.3378; Found 811.3372.

(S)-2-((6-(((S)-2-(trifluoromethylsulfonamido)-3-(1-(triisopropylsilyl)-1H-indol-3-yl)propanoyl)oxy)hexyl)carbamoyl)morpholino benzoate (1b)

^1^H NMR (500 MHz, CDCl_3_) δ 7.99 (d, J = 7.5 Hz, 2H), 7.59 – 7.50 (m, 2H), 7.47 (d, J = 7.7 Hz, 1H), 7.42 (t, J = 7.8 Hz, 2H), 7.18 – 7.08 (m, 3H), 6.62 (s, 1H), 6.39 (d, J = 1.6 Hz, 1H), 4.55 – 4.48 (m, 1H), 4.32 (s, 1H), 4.17 – 3.83 (m, 4H), 3.47 (d, J = 5.2 Hz, 1H), 3.35 (d, J = 6.0 Hz, 2H), 3.25 (d, J = 5.8 Hz, 2H), 2.92 (d, J = 5.9 Hz, 1H), 2.76 (s, 1H), 1.76 – 1.61 (m, 3H), 1.59 – 1.44 (m, 4H), 1.35 – 1.23 (m, 5H), 1.12 (d, J = 7.6 Hz, 18H). ^13^C NMR (125 MHz, CDCl_3_) δ 170.4, 168.5, 164.4, 141.2, 133.3, 130.6, 130.3, 129.5, 128.9, 128.5, 121.9, 119.9, 119.5 (q, J = 320.8 Hz), 118.3, 114.1, 110.6, 75.1, 66.0, 65.3, 58.7, 57.8, 56.3, 38.7, 29.4, 29.2, 28.1, 26.1, 25.2, 18.1, 12.8. HR-MS (ESI-TOF) m/z: [M+H]^+^Calcd for C_39_H_56_F_3_N_4_O_8_SSi 825.3535; Found: 825.3540.

(S)-2-(((S)-4-methyl-1-oxo-1-((4-((N^α^-((trifluoromethyl)sulfonyl)-1-(triisopropylsilyl)-L-tryptophyl)oxy)butyl)amino)pentan-2-yl)carbamoyl)morpholino benzoate (1c)

^1^H NMR (600 MHz, CDCl_3_) δ 7.97 (d, J = 7.6 Hz, 2H), 7.80 (brs, 1H), 7.61 – 7.52 (m, 2H), 7.46 (d, J = 8.1 Hz, 1H), 7.41 (t, J = 7.7 Hz, 2H), 7.19 (s, 1H), 7.16 – 7.06 (m, 4H), 4.59 (s, 1H), 4.44 (dd, J = 7.4, 6.0 Hz, 1H), 4.30 (s, 1H), 4.19 – 4.10 (m, 1H), 4.08 – 3.98 (m, 1H), 3.98 – 3.80 (m, 2H), 3.38 (dd, J = 14.6, 5.4 Hz, 2H), 3.30 (dd, J = 14.6, 8.2 Hz, 1H), 3.15 (pd, J = 13.0, 6.7 Hz, 2H), 2.92 (d, J = 10.7 Hz, 1H), 2.70 (s, 1H), 1.74 – 1.52 (m, 8H), 1.49 – 1.38 (m, 2H), 1.31 – 1.19 (m, 1H), 1.10 (dd, J = 7.8, 1.7 Hz, 18H), 0.93 (d, J = 5.7 Hz, 3H), 0.90 (d, J = 5.8 Hz, 3H).^13^C NMR (150 MHz, CDCl_3_) δ 171. 5, 170.2, 168.4, 164.0, 140.7, 133.0, 130.1, 129.9, 129.0, 128.2, 128.1, 121.2, 119.3, 119.0 (q, J = 320.9 Hz), 117.8, 113.6, 110.8, 74.5, 65.1, 64.8, 58.2, 57.7, 55.6, 50.7, 41.0, 38.6, 28.5, 25.2, 25.0, 24.4, 22.4, 21.4, 17.6, 12.3. HR-MS (ESI-TOF) m/z: [M+H]^+^ Calcd for C_43_H_63_F_3_N_5_O_9_SSi 910.4062; Found: 910.4037.

(S)-2-(((2S,3S)-3-methyl-1-oxo-1-((4-((1-(triisopropylsilyl)-L-tryptophyl)oxy)butyl)amino)pentan-2-yl)carbamoyl)morpholino benzoate--trifluoro(hydrosulfonyl)methane (1/1) (1d)

^1^H NMR (600 MHz, CDCl_3_) δ 7.99 (d, J = 7.6 Hz, 2H), 7.57 (dd, J = 9.8, 3.7 Hz, 2H), 7.47 (d, J = 8.0 Hz, 1H), 7.44 (t, J = 7.8 Hz, 2H), 7.40 (d, J = 9.3 Hz, 1H), 7.21 – 7.08 (m, 4H), 6.81 (s, 1H), 4.51 – 4.44 (m, 1H), 4.38 – 4.26 (m, 2H), 4.17 – 4.10 (m, 1H), 4.08 – 4.02 (m, 1H), 3.99 (s, 1H), 3.92 (s, 1H), 3.48 – 3.35 (m, 2H), 3.32 (dd, J = 14.7, 7.6 Hz, 1H), 3.18 (dd, J = 12.8, 6.6 Hz, 2H), 2.99 – 2.90 (m, 1H), 2.73 (s, 1H), 1.87 (d, J = 6.5 Hz, 1H), 1.72 – 1.62 (m, 3H), 1.61 – 1.49 (m, 3H), 1.49 – 1.40 (m, 2H), 1.31 – 1.18 (m, 1H), 1.12 (dd, J = 7.6, 1.4 Hz, 18H), 0.93 (d, J = 6.8 Hz, 3H), 0.90 (t, J = 7.4 Hz, 3H). ^13^C NMR (150 MHz, CDCl_3_) δ 170.5, 170.1, 168.3, 164.0, 140.7, 132.9, 130.1, 129.9, 129.0, 128.3, 128.1, 121.3, 119.3, 118.9 (q, J = 321.0 Hz), 117.8, 113.6, 110.6, 74.6, 65.2, 64.8, 58.3, 57.6, 56.7, 55.7, 38.5, 36.8, 28.6, 25.3, 25.0, 24.4, 17.6, 15.0, 12.3, 10.6. HR-MS (ESI-TOF) m/z: [M+H]^+^ Calcd for C_43_H_63_F_3_N_5_O_9_SSi 910.4062; Found 910.4042.

(S)-2-(((S)-3-methyl-1-oxo-1-((4-((1-(triisopropylsilyl)-L-tryptophyl)oxy)butyl)amino)butan-2-yl)carbamoyl)morpholino benzoate--trifluoro(hydrosulfonyl)methane (1/1) (1e)

^1^H NMR (600 MHz, CDCl_3_) δ 7.99 (d, J = 7.6 Hz, 2H), 7.57 (t, J = 8.0 Hz, 2H), 7.47 (d, J = 8.0 Hz, 1H), 7.43 (t, J = 7.8 Hz, 2H), 7.31 (d, J = 9.3 Hz, 1H), 7.21 (d, J = 9.1 Hz, 1H), 7.17 – 7.08 (m, 3H), 6.80 (s, 1H), 4.53 – 4.41 (m, 1H), 4.31 (s, 2H), 4.18 – 4.08 (m, 1H), 4.04 (dt, J = 27.7, 11.3 Hz, 2H), 3.92 (s, 2H), 3.44 (d, J = 6.9 Hz, 1H), 3.35 (ddd, J = 22.1, 14.7, 6.5 Hz, 2H), 3.21 – 3.14 (m, 2H), 3.00 – 2.90 (m, 1H), 2.74 (s, 1H), 2.15 – 2.05 (m, 1H), 1.73 – 1.60 (m, 3H), 1.60 – 1.52 (m, 2H), 1.52 – 1.38 (m, 2H), 1.11 (d, J = 7.6 Hz, 18H), 0.95 (t, J = 7.5 Hz, 6H). ^13^C NMR (150 MHz, CDCl_3_) δ 170.4, 170.1, 168.4, 164.0, 140.7, 132.9, 130.1, 129.9, 129.0, 128.3, 128.0, 121.3, 119.3, 119.0 (q, J = 321.0 Hz), 117.8, 113.6, 110.5, 74.6, 65.3, 64.8, 58.4, 57.6, 55.7, 38.4, 30.8, 28.7, 25.4, 24.9, 18.8, 17.8, 17.6, 12.3. HR-MS (ESI-TOF) m/z: [M+H]^+^Calcd for C_42_H_61_F_3_N_5_O_9_SSi 896.3906; Found: 896.3898.

(S)-2-(((S)-3-(4-chlorophenyl)-1-oxo-1-((4-((1-(triisopropylsilyl)-L-tryptophyl)oxy)butyl)amino)propan-2-yl)carbamoyl)morpholino benzoate--trifluoro(hydrosulfonyl)methane (1/1) (1f)

^1^H NMR (600 MHz, CDCl_3_) δ 8.03 – 7.98 (m, 2H), 7.57 (t, J = 7.5 Hz, 1H), 7.54 (d, J = 7.3 Hz, 1H), 7.48 – 7.41 (m, 3H), 7.23 (t, J = 12.2 Hz, 3H), 7.16 – 7.08 (m, 6H), 6.40 (s, 1H), 4.68 (d, J = 7.4 Hz, 1H), 4.50 (s, 1H), 4.27 (d, J = 7.1 Hz, 1H), 4.10 – 3.96 (m, 3H), 3.90 (s, 1H), 3.78 (s, 1H), 3.42 (s, 1H), 3.39 – 3.31 (m, 2H), 3.13 – 3.03 (m, 2H), 3.02 – 2.85 (m, 3H), 2.50 (s, 1H), 1.71 – 1.61 (m, 3H), 1.46 – 1.39 (m, 2H), 1.37 – 1.27 (m, 2H), 1.11 (dd, J = 7.6, 1.0 Hz, 18H). ^13^C NMR (150 MHz, CDCl_3_) δ 170.1, 169.7, 168.3, 164.0, 140.7, 134.5, 133.0, 132.4, 130.3, 130.1, 129.9, 129.0, 128.23, 128.18, 128.1, 121.3, 119.3, 119.0 (q, J = 320.9 Hz), 117.8, 113.7, 110.3, 74.3, 65.3, 64.7, 57.9, 57.5, 55.7, 53.3, 38.3, 37.5, 28.8, 25.3, 24.7, 17.6, 12.3. HR-MS (ESI-TOF) m/z: [M+H]^+^Calcd for C_46_H_60_ClF_3_N_5_O_10_SSi 978.3516; Found 978.3505.

(S)-2-(((S)-4-(benzyloxy)-1,4-dioxo-1-((4-((N^α^-((trifluoromethyl)sulfonyl)-1-(triisopropylsilyl)-L-tryptophyl)oxy)butyl)amino)butan-2-yl)carbamoyl)morpholino benzoate (1g)

^1^H NMR (400 MHz, CDCl_3_) δ 7.99 (d, J = 7.5 Hz, 2H), 7.68 (s, 1H), 7.61 – 7.51 (m, 2H), 7.50 – 7.40 (m, 3H), 7.38 – 7.27 (m, 5H), 7.19 – 7.07 (m, 3H), 6.97 (s, 1H), 6.62 (t, J = 5.6 Hz, 1H), 5.13 (s, 2H), 4.81 (dd, J = 13.0, 7.0 Hz, 1H), 4.51 (s, 1H), 4.35 (d, J = 8.0 Hz, 1H), 4.19 – 3.84 (m, 5H), 3.44 (d, J = 6.4 Hz, 1H), 3.35 (d, J = 6.1 Hz, 2H), 3.17 – 2.87 (m, 4H), 2.84 – 2.64 (m, 2H), 1.75 – 1.59 (m, 3H), 1.56 – 1.46 (m, 2H), 1.40 (dt, J = 20.8, 6.9 Hz, 2H), 1.16 – 1.06 (m, 18H).^13^C NMR (125 MHz, CDCl_3_) δ 171.4, 170.5, 169.8, 168.9, 164.4, 141.3, 135.3, 133.4, 130.6, 130.4, 129.5, 128.8, 128.6, 128.5, 128.4, 128.3, 121.8, 119.9, 119.5 (q, J = 321.1 Hz), 118.3, 114.1, 110.6, 74.9, 66.9, 65.9, 65.3, 58.3, 57.9, 56.1, 48.8, 38.8, 36.0, 29.4, 26.0, 25.0, 18.1, 12.8. HR-MS (ESI): m/z calcd for C_48_H_63_F_3_N_5_O_11_SSi [M+H]^+^: 1002.3961, found: 1002.3937.

4.2 Analytical data of products

N,N'-((1^2^S,1^4^R,14^2^S,14^4^R,4S,17S)-5,13,18,26-tetraoxo-2^1^,15^1^-bis(triisopropylsilyl)-2^1^H,15^1^H-6,19-dioxa-12,25-diaza-1(4,2),14(2,4)-dimorpholina-2,15(4,3)-diindolacyclohexacosaphane-4,17-diyl)bis(1,1,1-trifluoromethanesulfonamide) (DC644)

The general procedure is followed using compound 1a (80.0 mg, 0.098 mmol), Pd(CH_3_CN)_2_Cl_2_ (5.1 mg, 20 mol %), AgOAc (32.9 mg, 0.19 mmol), and Na_3_PO_4_ (48.5 mg, 0.29 mmol) at 85 ^o^C in DCE (2.5 mL). Purification by column chromatography on silica gel (PE/EA 4:1-2:1) yields DC644 (21.8 mg, 32%) as white solid. m. p. 113-115 ºC.^1^H NMR (500 MHz, CDCl_3_) δ 11.41 (d, J = 5.6 Hz, 2H), 7.36 (d, J = 8.4 Hz, 2H), 7.19 (s, 2H), 7.11 (t, J = 8.0 Hz, 2H), 6.88 (d, J = 7.7 Hz, 2H), 6.73 (t, J = 6.1 Hz, 2H), 4.57 (dd, J = 10.9, 1.9 Hz, 2H), 4.33 – 4.17 (m, 8H), 4.06 – 3.95 (m, 2H), 3.83 (d, J = 12.7 Hz, 2H), 3.51 – 3.35 (m, 6H), 3.27 – 3.08 (m, 6H), 2.59 – 2.47 (m, 2H), 1.82 – 1.62 (m, 6H), 1.62 – 1.53 (m, 4H), 1.51 – 1.41 (m, 2H), 1.13 (dd, J = 7.5, 4.7 Hz, 36H), 1.05 (d, J = 6.8 Hz, 2H), 0.88 (t, J = 6.8 Hz, 2H). ^13^C NMR (125 MHz, CDCl_3_) δ 170.8, 167.9, 142.8, 142.2, 131.4, 124.2, 121.4, 112.0, 110.0, 109.6, 74.3, 65.6, 65.1, 60.1, 59.0, 49.3, 38.0, 29.2, 29.1, 27.9, 22.7, 17.6, 17.5, 12.4. ^19^F NMR (375 MHz, CDCl_3_) δ -78.3. LR-MS (ESI): 1377.8 [M+H]^+^. HR-MS (ESI-TOF) m/z: [M+H]^+^Calcd for C_62_H_95_F_6_N_8_O_12_S_2_Si_2_ 1377.5948; Found 1377.5938.

N,N'-((1^2^S,1^4^R,15^2^S,15^4^R,4S,18S)-5,14,19,28-tetraoxo-2^1^,16^1^-bis(triisopropylsilyl)-2^1^H,16^1^H-6,20-dioxa-13,27-diaza-1(4,2),15(2,4)-dimorpholina-2,16(4,3)-diindolacyclooctacosaphane-4,18-diyl)bis(1,1,1-trifluoromethanesulfonamide) (DC645)

The general procedure is followed using compound 1b (80.0 mg, 0.097 mmol), Pd(CH_3_CN)_2_Cl_2_ (5.0 mg, 20 mol %), AgOAc (32.3 mg, 0.19 mmol), and Na_3_PO_4_ (47.7 mg, 0.29 mmol) at 85 ^o^C in DCE (2.5 mL). Purification by column chromatography on silica gel (PE/EA 4:1-2:1) yields DC645 (20.5 mg, 30%) as white solid. m. p. 103-105 ºC. ^1^H NMR (600 MHz, CDCl_3_) δ 11.38 (d, J = 5.5 Hz, 2H), 7.35 (d, J = 8.4 Hz, 2H), 7.19 (s, 2H), 7.10 (t, J = 8.0 Hz, 2H), 6.88 (d, J = 7.7 Hz, 2H), 6.80 (t, J = 5.8 Hz, 2H), 4.56 (dd, J = 11.0, 2.1 Hz, 2H), 4.40 – 4.31 (m, 2H), 4.28 (d, J = 6.8 Hz, 4H), 4.23 – 4.11 (m, 2H), 4.06 – 3.97 (m, 2H), 3.82 (d, J = 12.8 Hz, 2H), 3.53 – 3.35 (m, 6H), 3.29 – 3.17 (m, 4H), 3.12 (dt, J = 19.7, 6.6 Hz, 2H), 2.61 – 2.48 (m, 2H), 1.79 – 1.60 (m, 12H), 1.60 – 1.48 (m, 4H), 1.48 – 1.35 (m, 4H), 1.12 (dd, J = 7.5, 5.0 Hz, 36H), 0.87 (dd, J = 12.8, 6.1 Hz, 2H). ^13^C NMR (150 MHz, CDCl_3_) δ 171.3, 168.6, 143.3, 142.6, 131.8, 124.6, 121.8, 112.5, 110.6, 110.0, 75.0, 66.1, 65.7, 60.7, 59.7, 49.7, 38.7, 29.7, 29.3, 28.5, 26.3, 25.8, 18.0, 17.9, 12.8. ^19^F NMR (375 MHz, CDCl_3_) δ -78.3. LR-MS (ESI): 1405.8 [M+H]^+^. HR-MS (ESI-TOF) m/z: [M+H]^+^ Calcd for C_64_H_99_F_6_N_8_O_12_S_2_Si_2_ 1405.6261; Found: 1405.6264.

N,N'-((1^2^R,1^4^S,16^2^R,16^4^S,4R,13R,19R,28R)-13,28-diisobutyl-5,12,15,20,27,30-hexaoxo-2^1^,17^1^-bis(triisopropylsilyl)-2^1^H,17^1^H-6,21-dioxa-11,14,26,29-tetraaza-1(4,2),16(2,4)-dimorpholina-2,17(4,3)-diindolacyclotriacontaphane-4,19-diyl)bis(1,1,1-trifluoromethanesulfonamide) (DC646)

The general procedure is followed using compound 1c (80.0 mg, 0.088 mmol), Pd(CH_3_CN)_2_Cl_2_ (4.6 mg, 20 mol %), AgOAc (29.3 mg, 0.17 mmol), and Na_3_PO_4_ (43.2 mg, 0.26 mmol) at 85 ^o^C in DCE (2.5 mL). Purification by column chromatography on silica gel (PE/EA 4:1-2:1) yields DC646 (27.0 mg, 39%) as white solid. m. p. 260-262 ºC. ^1^H NMR (600 MHz, CDCl_3_) δ 11.12 (d, J = 5.7 Hz, 2H), 7.53 (t, J = 5.0 Hz, 2H), 7.37 (d, J = 8.3 Hz, 2H), 7.32 (d, J = 9.2 Hz, 2H), 7.22 (s, 2H), 7.13 (t, J = 8.0 Hz, 2H), 6.91 (d, J = 7.7 Hz, 2H), 4.58 (dd, J = 15.0, 8.2 Hz, 2H), 4.50 (ddd, J = 19.5, 13.4, 6.9 Hz, 4H), 4.38 – 4.30 (m, 2H), 4.29 – 4.18 (m, 4H), 4.13 – 3.97 (m, 2H), 3.70 (d, J = 12.6 Hz, 2H), 3.51 (dd, J = 14.8, 4.1 Hz, 2H), 3.43 (td, J = 11.0, 4.5 Hz, 2H), 3.39 – 3.29 (m, 2H), 3.24 – 3.11 (m, 4H), 3.07 – 2.93 (m, 2H), 2.48 (t, J = 11.9 Hz, 2H), 1.83 (ddd, J = 14.6, 9.9, 5.1 Hz, 2H), 1.68 (dt, J = 14.8, 7.3 Hz, 6H), 1.61 – 1.38 (m, 12H), 1.13 (t, J = 7.8 Hz, 36H), 0.75 (t, J = 5.6 Hz, 12H). ^13^C NMR (125 MHz, CDCl_3_) δ 171.0, 170.8, 168.6, 142.8, 142.2, 131.2, 124.2, 121.5, 118.8 (d, J = 321.8 Hz), 112.1, 109.9, 109.8, 74.5, 65.4, 64.1, 59.7, 59.2, 50.5, 49.3, 41.8. 38.8, 29.2, 26.3, 24.9, 24.3, 22.2, 21.9, 17.5, 17.4, 12.3. ^19^F NMR (375 MHz, CDCl_3_) δ -78.3. LR-MS (ESI): 1576.0 [M+H]^+^. HR-MS (ESI-TOF) m/z: [M+H]^+^ Calcd for C_72_H_113_F_6_N_10_O_14_S_2_Si_2_ 1575.7316; Found 1575.7312.

N,N'-((1^2^S,1^4^R,16^2^S,16^4^R,4S,13S,19S,28S)-13,28-di((S)-sec-butyl)-5,12,15,20,27,30-hexaoxo-2^1^,17^1^-bis(triisopropylsilyl)-2^1^H,17^1^H-6,21-dioxa-11,14,26,29-tetraaza-1(4,2),16(2,4)-dimorpholina-2,17(4,3)-diindolacyclotriacontaphane-4,19-diyl)bis(1,1,1-trifluoromethanesulfonamide) (DC647)

The general procedure is followed using compound 1d (80.0 mg, 0.088 mmol), Pd(CH_3_CN)_2_Cl_2_ (4.6 mg, 20 mol %), AgOAc (29.3 mg, 0.17 mmol), and Na_3_PO_4_ (43.2 mg, 0.26 mmol) (80.0 mg) at 85 ^o^C in DCE (2.5 mL). Purification by column chromatography on silica gel (PE/EA 4:1-2:1) yields DC647 (31.9 mg, 46%) as white solid. m. p. 260-262 ºC. ^1^H NMR (500 MHz, CDCl_3_) δ 11.14 (d, J = 5.9 Hz, 2H), 7.49 (t, J = 5.0 Hz, 2H), 7.44 (d, J = 9.6 Hz, 2H), 7.37 (d, J = 8.3 Hz, 2H), 7.21 (s, 2H), 7.13 (t, J = 8.0 Hz, 2H), 6.93 (d, J = 7.8 Hz, 2H), 4.57 (td, J = 10.9, 2.8 Hz, 2H), 4.47 (ddd, J = 16.7, 10.2, 4.6 Hz, 4H), 4.35 – 4.20 (m, 6H), 4.11 – 3.99 (m, 2H), 3.70 (d, J = 12.5 Hz, 2H), 3.57 – 3.39 (m, 4H), 3.38 – 3.25 (m, 2H), 3.25 – 3.04 (m, 6H), 2.56 – 2.44 (m, 2H), 1.86 (ddd, J = 14.2, 10.0, 5.2 Hz, 2H), 1.78 – 1.55 (m, 12H), 1.52 – 1.36 (m, 4H), 1.13 (t, J = 7.5 Hz, 36H), 1.02 – 0.91 (m, 2H), 0.80 (d, J = 6.8 Hz, 6H), 0.74 (t, J = 7.4 Hz, 6H). ^13^C NMR (125 MHz, CDCl_3_) δ 171.0, 170.1, 168.9, 142.8 142.2, 131.2, 124.2, 121.5, 118.8 (q, J = 322.4 Hz), 112.1, 109.94, 109.86, 74.8, 65.3, 63.8, 59.7, 59.6, 56.0, 49.3, 38.8, 37.9, 29.3, 26.4, 24.8, 23.9, 17.5, 17.4, 14.8, 12.3, 10.7. ^19^F NMR (375 MHz, CDCl_3_) δ -78.3. LR-MS (ESI): 1597.8 [M+Na]^+^. HR-MS (ESI-TOF) m/z: [M-2H]^-2^ C_72_H_110_F_6_N_10_O_14_S_2_Si_2_ 786.3549; Found 786.3562.

N,N'-((1^2^S,1^4^R,16^2^S,16^4^R,4S,13S,19S,28S)-13,28-diisopropyl-5,12,15,20,27,30-hexaoxo-2^1^,17^1^-bis(triisopropylsilyl)-2^1^H,17^1^H-6,21-dioxa-11,14,26,29-tetraaza-1(4,2),16(2,4)-dimorpholina-2,17(4,3)-diindolacyclotriacontaphane-4,19-diyl)bis(1,1,1-trifluoromethanesulfonamide) (DC648)

The general procedure is followed using compound 1e (80.0 mg, 0.089 mmol), Pd(CH_3_CN)_2_Cl_2_ (4.6 mg, 20 mol %), AgOAc (29.8 mg, 0.18 mmol), and Na_3_PO_4_ (43.9 mg, 0.27 mmol) at 85 ^o^C in DCE (2.5 mL). Purification by column chromatography on silica gel (PE/EA 4:1-2:1) yields DC648 (29.0 mg, 42%) as white solid. m. p. 293-295 ºC. ^1^H NMR (500 MHz, CDCl_3_) δ 11.19 (d, J = 5.9 Hz, 2H), 7.52 (dd, J = 14.1, 7.3 Hz, 4H), 7.40 (d, J = 8.3 Hz, 2H), 7.24 (s, 2H), 7.16 (t, J = 8.0 Hz, 2H), 6.96 (d, J = 7.8 Hz, 2H), 4.62 (td, J = 11.0, 2.4 Hz, 2H), 4.52 (ddd, J = 16.4, 10.3, 4.3 Hz, 4H), 4.36 – 4.18 (m, 6H), 4.15 – 4.02 (m, 2H), 3.73 (d, J = 12.5 Hz, 2H), 3.51 (ddd, J = 15.0, 13.0, 4.5 Hz, 4H), 3.37 – 3.11 (m, 8H), 2.64 – 2.42 (m, 2H), 2.11 – 1.95 (m, 2H), 1.90 (ddd, J = 14.1, 10.0, 4.7 Hz, 2H), 1.78 – 1.58 (m, 10H), 1.57 – 1.42 (m, 2H), 1.16 (t, J = 7.4 Hz, 36H), 0.84 (dd, J = 15.4, 6.8 Hz, 12H). ^13^C NMR (125 MHz, CDCl_3_) δ 171.0, 170.0, 169.0, 142.8, 142.2, 131.2, 124.2, 121.5, 118.8 (q, J = 321.7 Hz), 112.1, 110.0, 109.8, 74.8, 65.3, 63.7, 59.7, 56.4, 49.3, 38.9, 31.9, 29.3, 26.4, 24.7, 18.8, 17.5, 17.5, 17.3. ^19^F NMR (375 MHz, CDCl_3_) δ -78.3. LR-MS (ESI): 1547.8. HR-MS (ESI-TOF) m/z: [M+H]^+^ Calcd for C_70_H_108_F_6_N_10_O_14_S_2_Si_2_ 1547.7003; Found 1547.7014.

N,N'-((1^2^S,1^4^R,16^2^S,16^4^R,4S,13S,19S,28S)-13,28-bis(4-chlorobenzyl)-5,12,15,20,27,30-hexaoxo-2^1^,17^1^-bis(triisopropylsilyl)-2^1^H,17^1^H-6,21-dioxa-11,14,26,29-tetraaza-1(4,2),16(2,4)-dimorpholina-2,17(4,3)-diindolacyclotriacontaphane-4,19-diyl)bis(1,1,1-trifluoromethanesulfonamide) (DC649)

The general procedure is followed using compound 1f (80.0 mg, 0.082 mmol), Pd(CH_3_CN)_2_Cl_2_ (4.2 mg, 20 mol %), AgOAc (27.3 mg, 0.163 mmol), and Na_3_PO_4_ (40.2 mg, 0.245 mmol) at 85 ^o^C in DCE (2.5 mL). Purification by column chromatography on silica gel (PE/EA 4:1-2:1) yields DC649 (22.4 mg, 32%) as white solid. m. p. 136-138 ºC. ^1^H NMR (500 MHz, CDCl_3_) δ 11.09 (d, J = 6.0 Hz, 2H), 7.54 (d, J = 9.2 Hz, 2H), 7.40 (d, J = 8.3 Hz, 2H), 7.27 (s, 2H), 7.25 (d, J = 5.1 Hz, 2H), 7.17 (t, J = 8.0 Hz, 2H), 7.00 (d, J = 8.4 Hz, 4H), 6.98 – 6.92 (m, 6H), 4.83 (dd, J = 16.0, 7.0 Hz, 2H), 4.60 (td, J = 11.0, 2.8 Hz, 2H), 4.44 (dd, J = 11.0, 2.1 Hz, 2H), 4.27 – 4.17 (m, 6H), 4.13 – 4.01 (m, 2H), 3.61 – 3.50 (m, 4H), 3.43 (td, J = 11.6, 5.4 Hz, 2H), 3.26 – 3.03 (m, 8H), 2.91 (ddd, J = 43.7, 13.6, 6.9 Hz, 4H), 2.51 – 2.33 (m, 2H), 1.88 – 1.75 (m, 2H), 1.70 (dt, J = 15.0, 7.5 Hz, 6H), 1.66 – 1.57 (m, 2H), 1.47 – 1.35 (m, 2H), 1.31 (dd, J = 14.6, 7.3 Hz, 2H), 1.14 (t, J = 8.0 Hz, 36H). ^13^C NMR (125 MHz, CDCl_3_) δ 171.6, 169.8, 169.3, 143.3, 142.6, 134.6, 132.7, 131.9, 131.0, 128.2, 124.8, 122.1, 119.3 (q, J = 321.4 Hz), 112.7, 110.6, 110.1, 75.0, 65.8, 64.0, 60.1, 59.8, 52.6, 49.9, 39.6, 39.2, 29.7, 26.8, 25.0, 18.0, 17.9, 12.8. ^19^F NMR (375 MHz, CDCl_3_) δ -78.3. LR-MS (ESI): 1713.8 [M+H]^+^. HR-MS (ESI-TOF) m/z: [M+H]^+^Calcd for C_78_H_107_Cl_2_F_6_N_10_O_14_S_2_Si_2_ 1711.6224; Found 1711.6176.

N-(1-((1^2^S,1^4^R,16^2^S,16^4^R,4S,13S,19S,28S)-28-isobutyl-5,12,15,20,27,30-hexaoxo-4,19-bis((trifluoromethyl)sulfonamido)-2^1^,17^1^-bis(triisopropylsilyl)-2^1^H,17^1^H-6,21-dioxa-11,14,26,29-tetraaza-1(4,2),16(2,4)-dimorpholina-2,17(4,3)-diindolacyclotriacontaphane-13-yl)-2-oxo-6,9,12,15-tetraoxa-3-azaheptadecan-17-yl)-5-((3aS,4S,6aR)-2-oxohexahydro-1H-thieno[3,4-d]imidazol-4-yl)pentanamide (BIOTIN-DC646)

^1^H NMR (500 MHz, Chloroform-d) δ = 11.23 (d, J=6.0, 1H), 11.19 (d, J=5.9, 1H), 8.13 (d, J=7.5, 1H), 7.73 – 7.42 (m, 2H), 7.39 – 7.30 (m, 4H), 7.20 (d, J=7.9, 2H), 7.10 (td, J=7.9, 5.5, 2H), 6.94 – 6.78 (m, 3H), 5.92 (s, 1H), 5.57 (d, J=89.7, 1H), 4.62 (dt, J=11.8, 4.8, 2H), 4.54 – 4.44 (m, 4H), 4.38 – 4.17 (m, 8H), 4.05 (dt, J=11.2, 5.1, 2H), 3.76 – 3.26 (m, 29H), 3.21 – 3.02 (m, 6H), 2.89 (ddd, J=32.2, 13.1, 5.4, 2H), 2.79 – 2.66 (m, 2H), 2.62 – 2.45 (m, 3H), 2.21 (dt, J=7.9, 4.0, 4H), 1.82 – 1.59 (m, 12H), 1.56 – 1.40 (m, 8H), 1.18 – 1.06 (m, 36H), 0.79 – 0.70 (m, 6H). ^13^C NMR (125 MHz, Chloroform-d) δ = 172.8, 171.2, 171.0, 170.9, 170.0, 169.2, 169.1, 168.5, 163.2, 142.8, 142.2, 131.4, 131.2, 130.4, 128.4, 124.1, 121.53, 121.45, 120.1, 117.5, 112.04, 111.98, 110.0, 109.9, 109.8, 74.6, 74.5, 70.0, 69.96, 69.92, 69.4, 67.7, 65.6, 65.5, 64.6, 64.5, 61.4, 59.9, 59.7, 59.0, 58.8, 55.0, 50.3, 50.2, 49.5, 49.3, 42.1, 40.1, 38.74, 38.67, 38.6, 38.3, 37.4, 35.2, 31.5, 29.9, 29.3, 29.2, 28.9, 27.5, 26.1, 26.0, 25.21, 25.17, 25.0, 24.3, 23.3, 22.5, 22.4, 22.2, 21.6, 17.6, 17.54, 17.51, 17.48, 13.7, 12.3, 10.5. ^19^F NMR (470 MHz, Chloroform-d) δ = -78.2, -78.3. HR-MS (ESI-TOF) m/z: [M+H]^+^ Calcd for C_90_ H_143_F_6_N_14_O_21_S_3_Si_2_ 2021.9152; Found 2021.9150.

4. NMR spectra of substrates and products

| 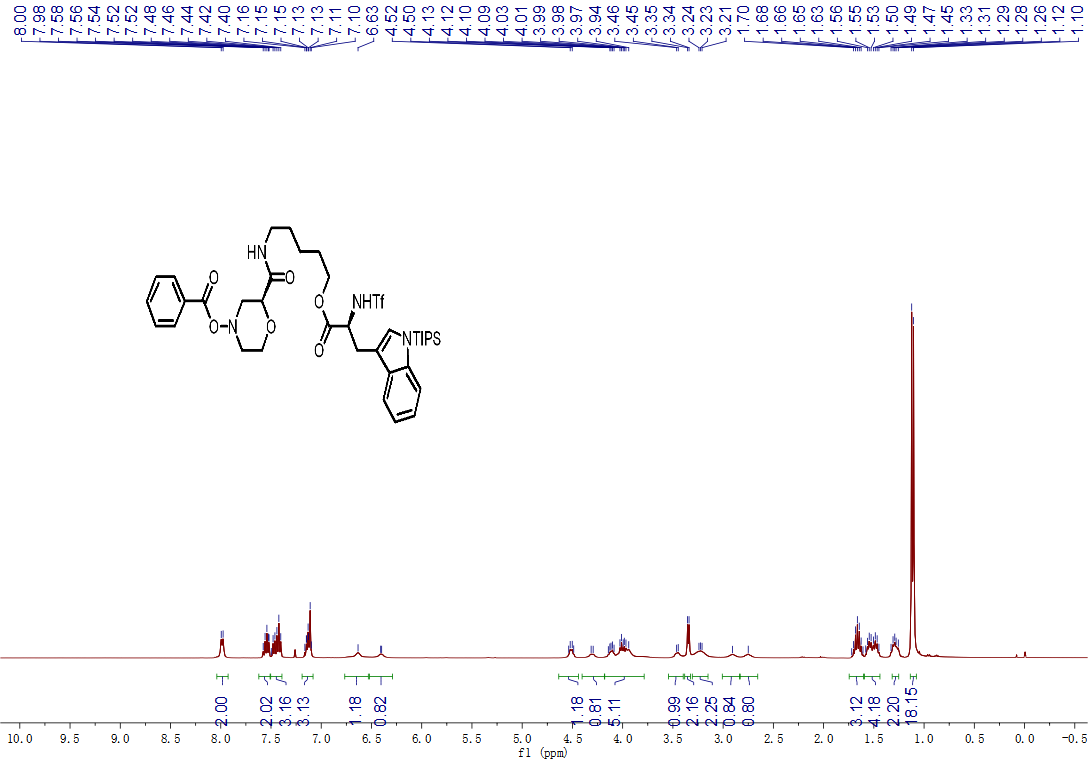  ^1^H-NMR spectrum of compound 1a |
| --- |
| 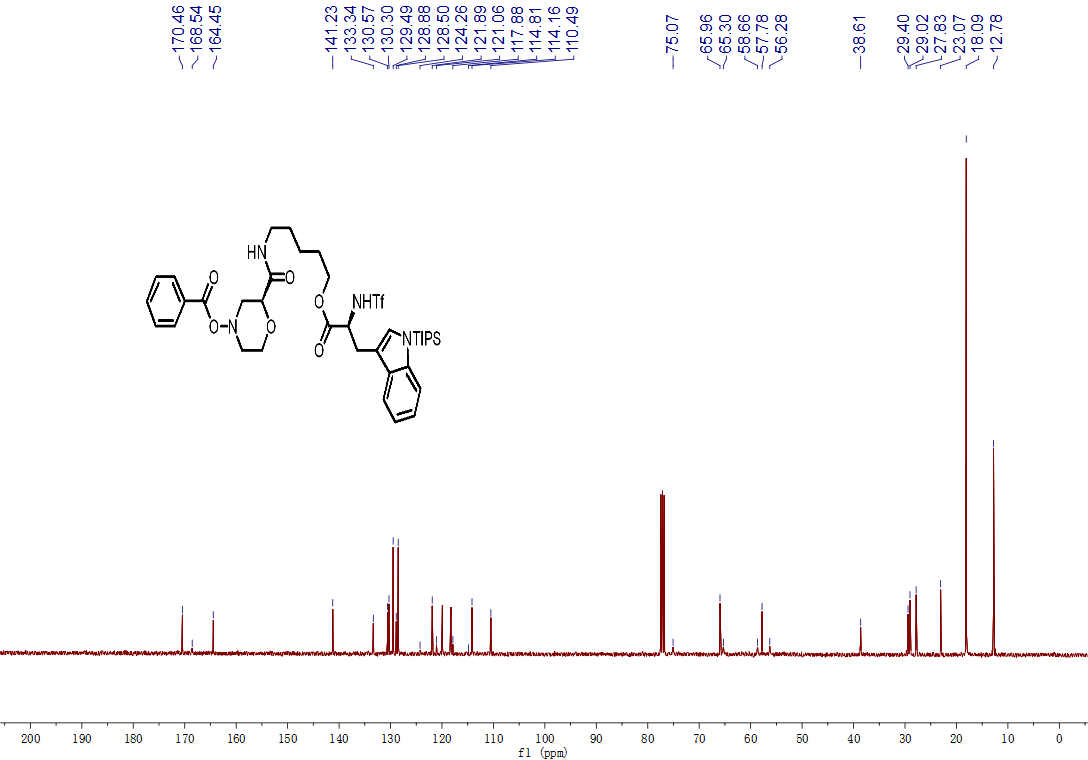  ^13^C-NMR spectrum of compound 1a |
| 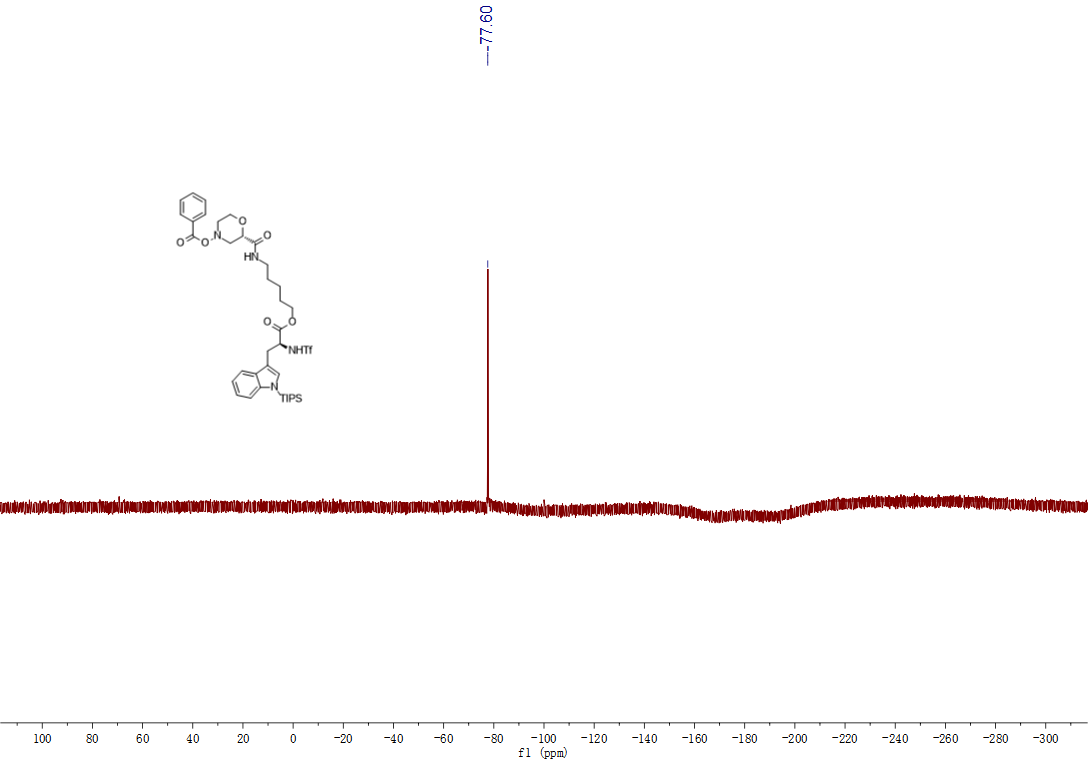  ^19^F-NMR spectrum of compound 1a |
| 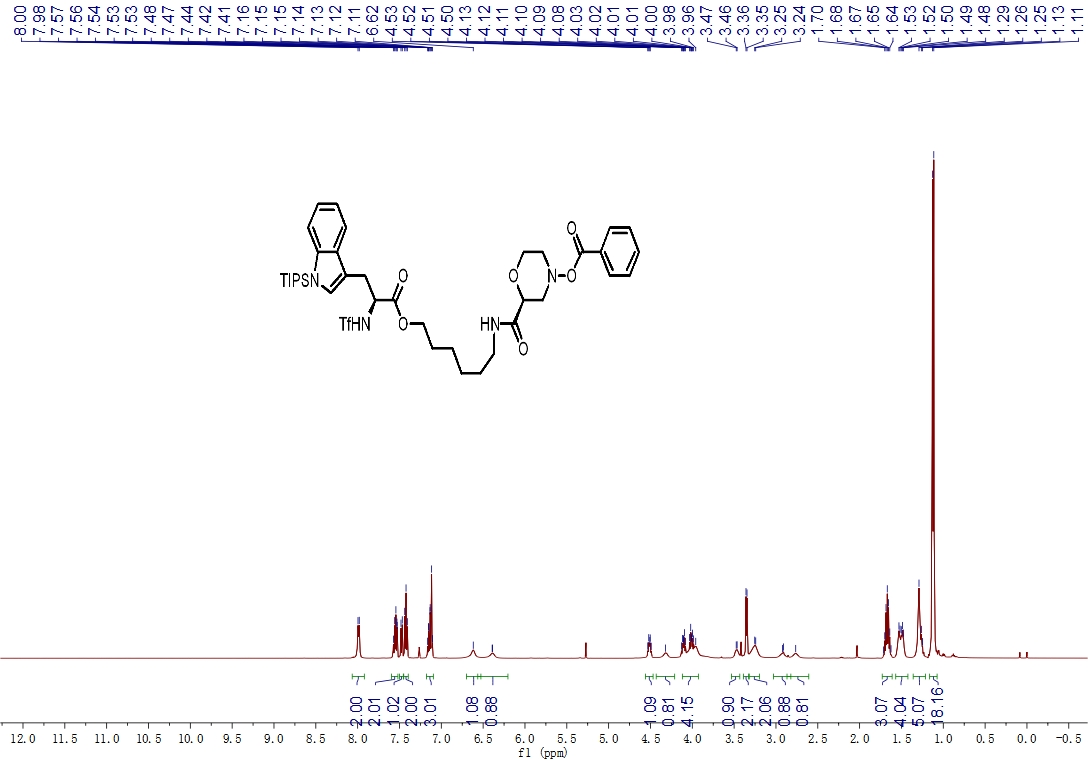  ^1^H-NMR spectrum of compound 1b |
| 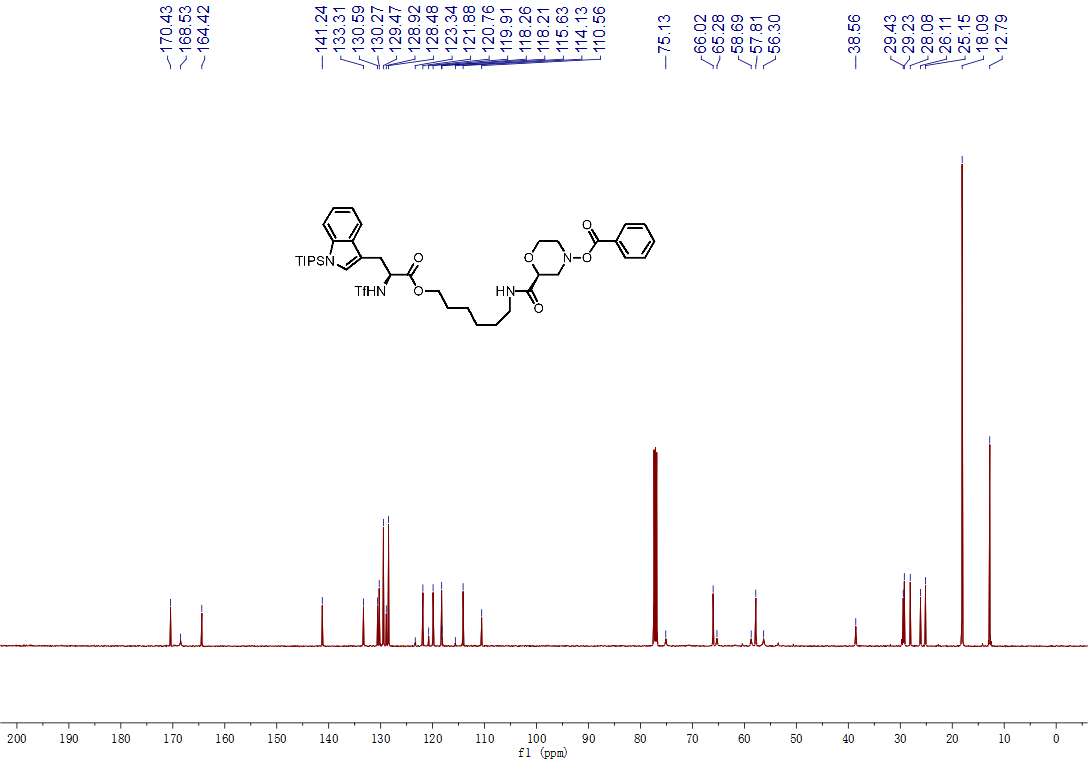  ^13^C-NMR spectrum of compound 1b |
|  |
| 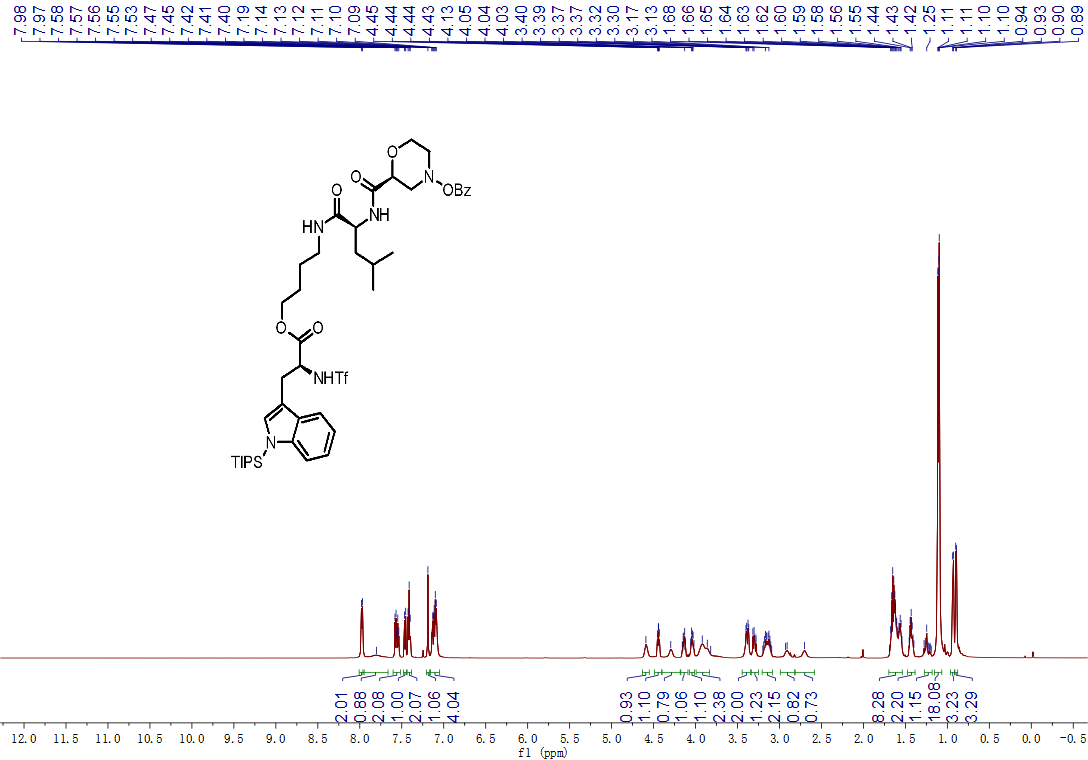  ^1^H-NMR spectrum of compound 1c |
| 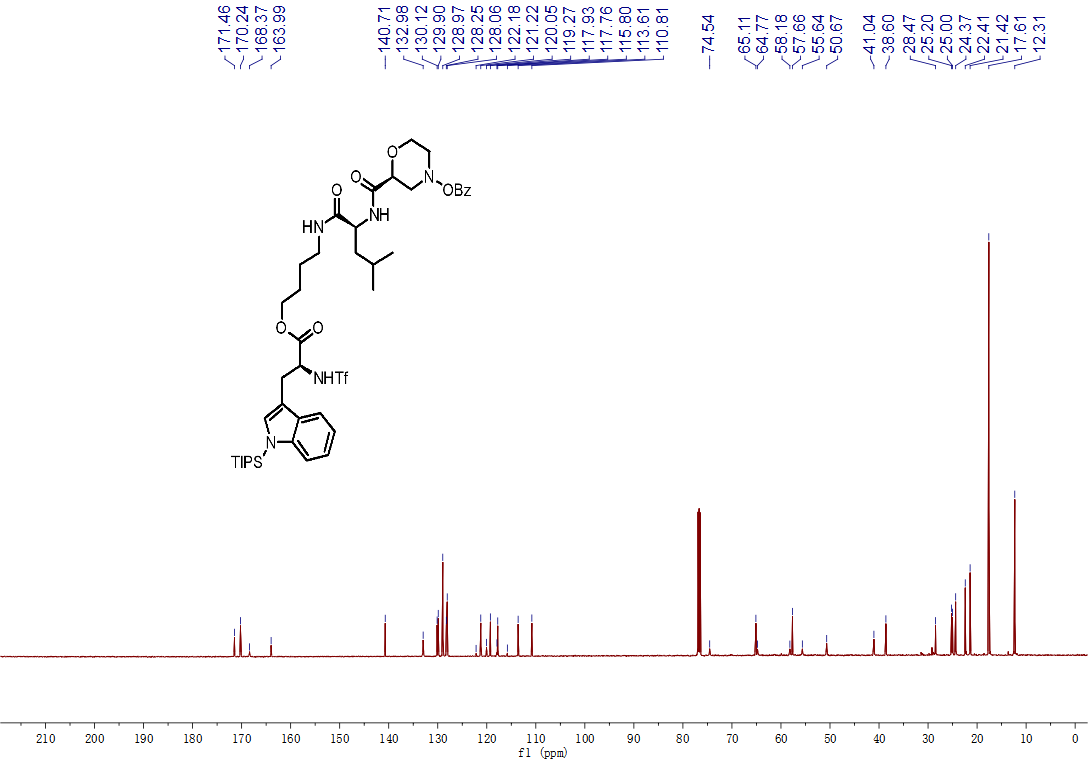  ^13^C-NMR spectrum of compound 1c |
|  |
| 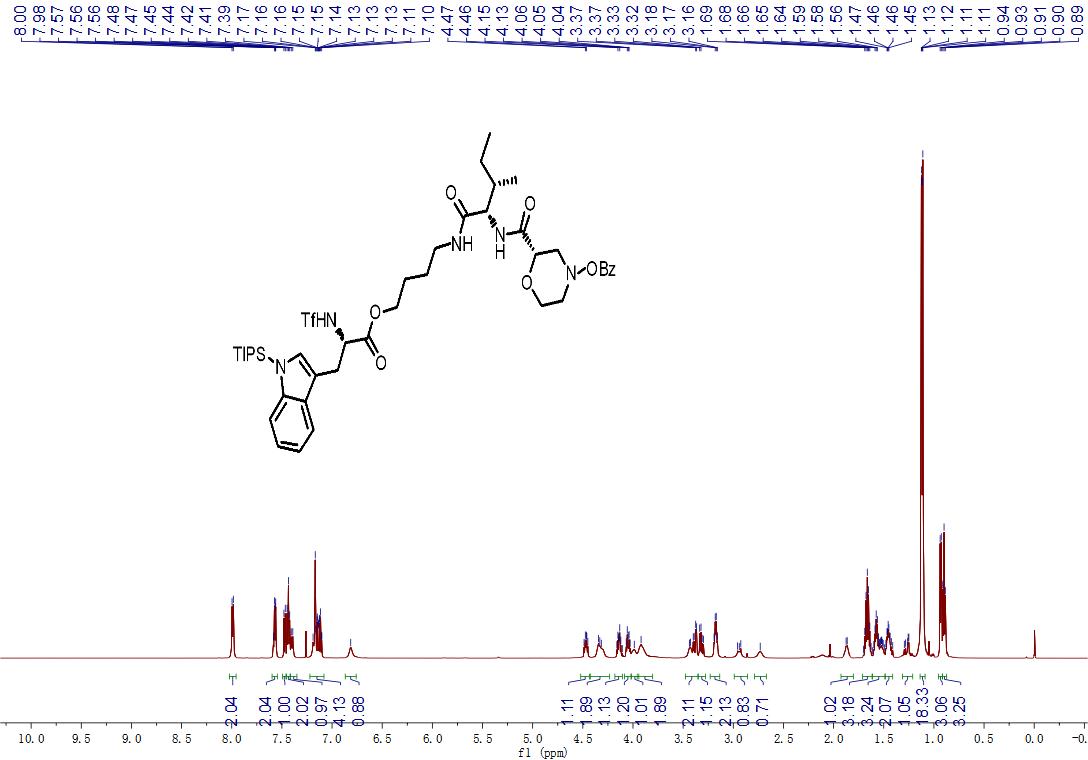  ^1^H-NMR spectrum of compound 1d |
| 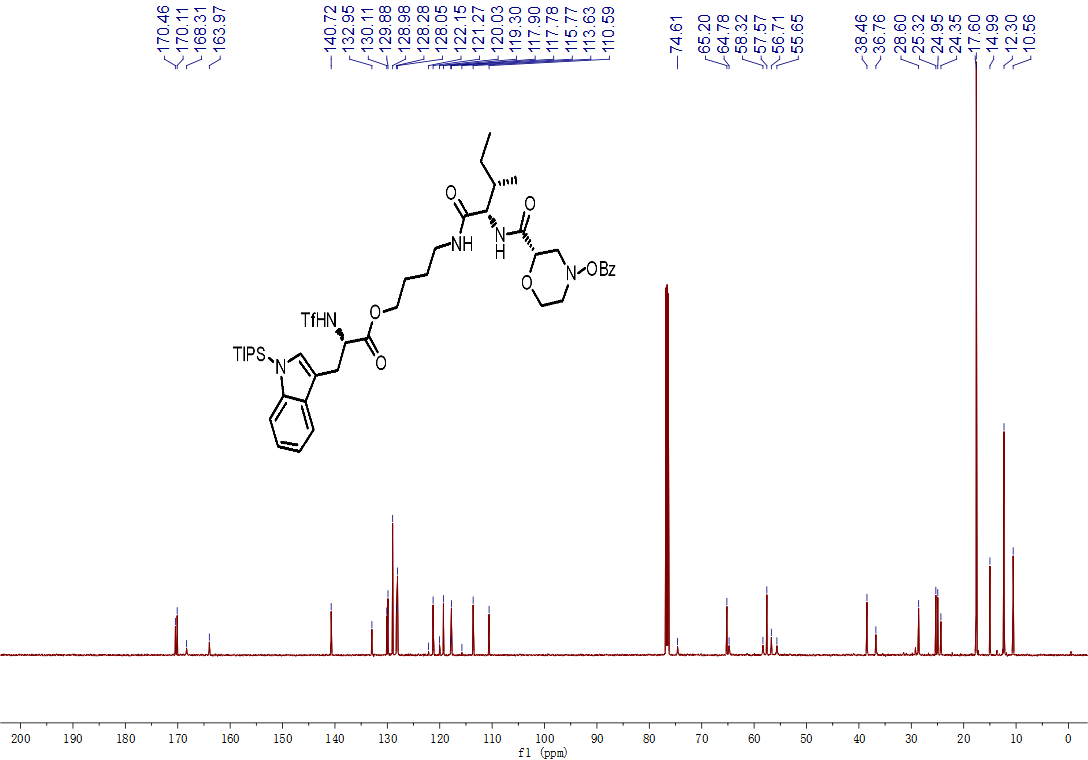  ^13^C-NMR spectrum of compound 1d |
| 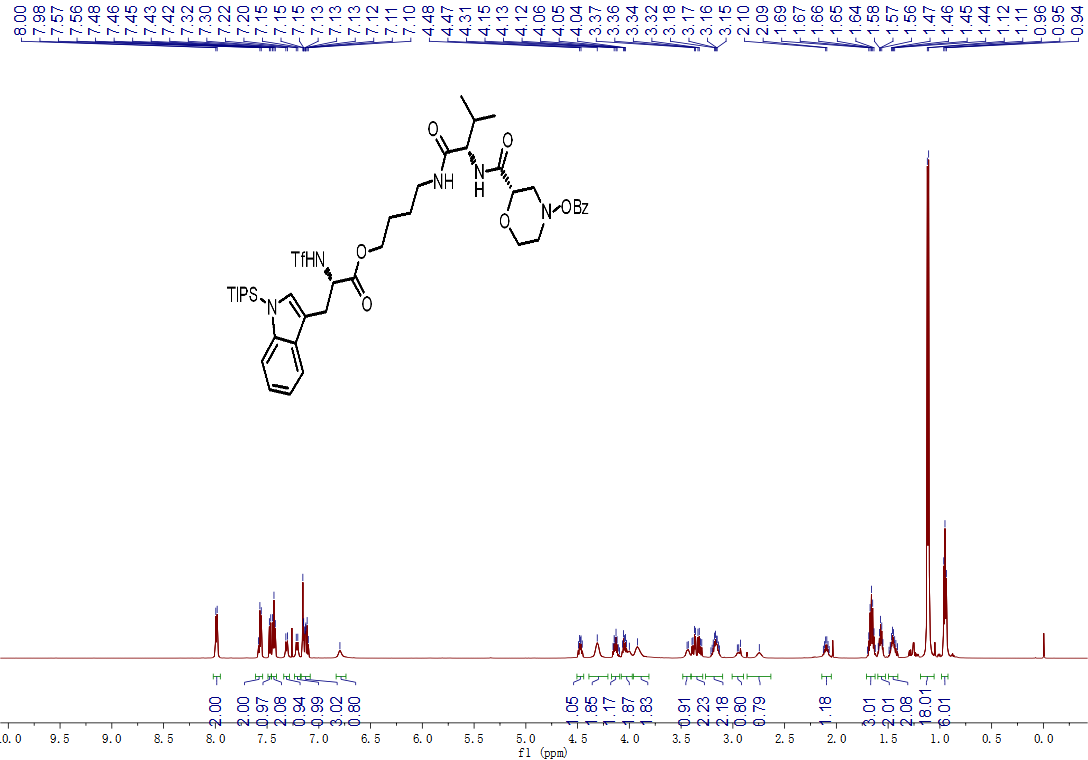  ^1^H-NMR spectrum of compound 1e |
| 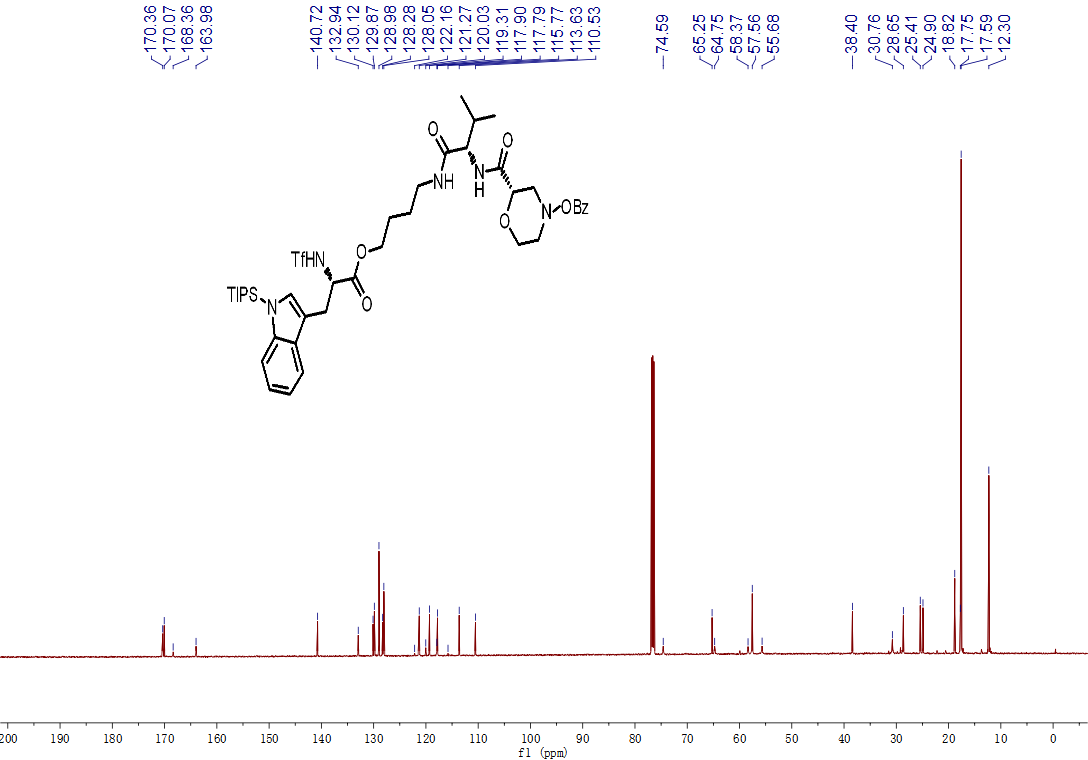  ^13^C-NMR spectrum of compound 1e |
|  |
|  |
| 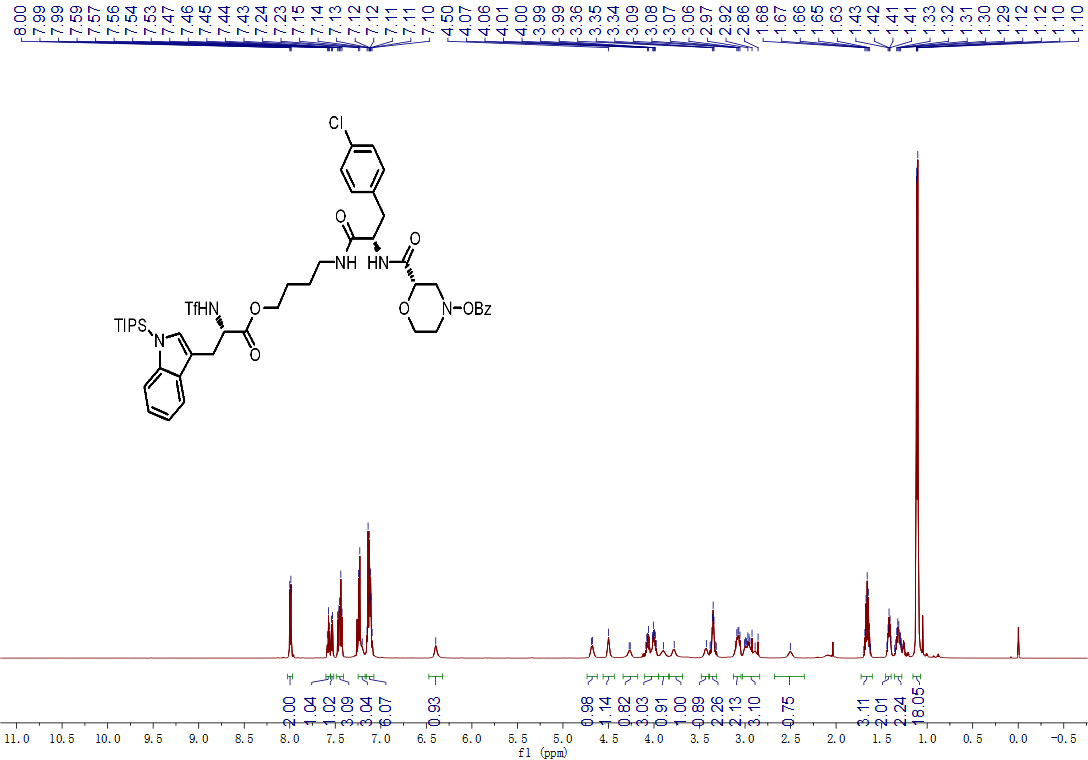  ^1^H-NMR spectrum of compound 1f |
| 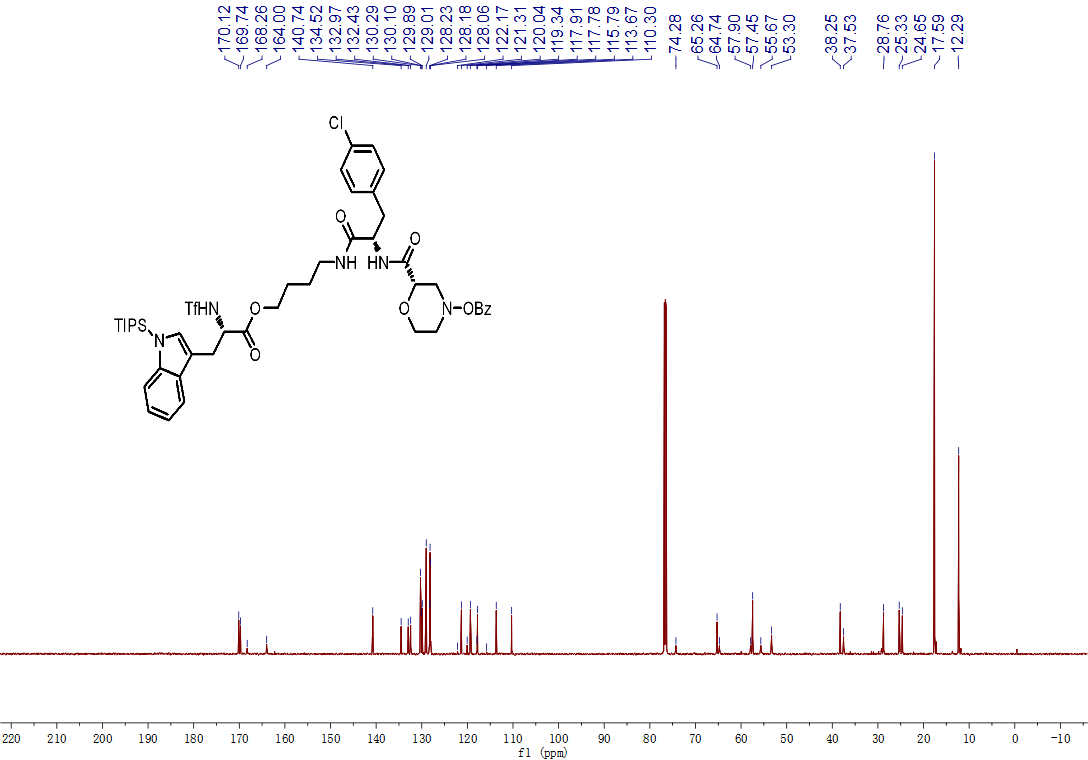  ^13^C-NMR spectrum of compound 1f |
| 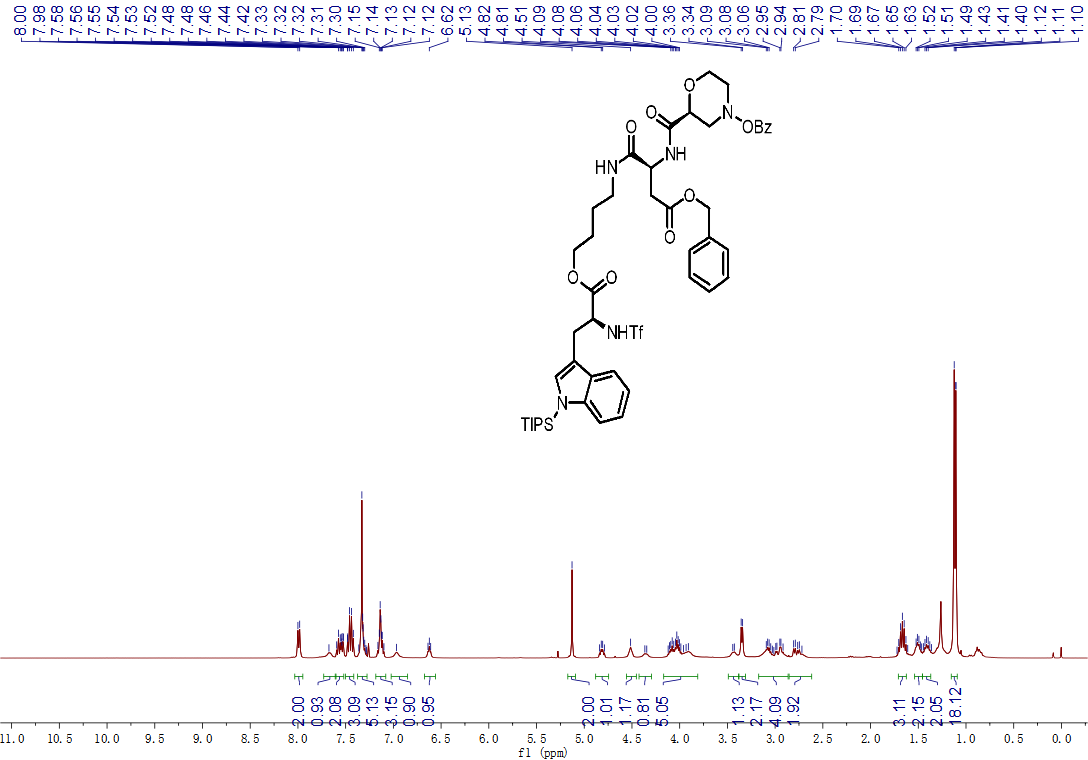  ^1^H-NMR spectrum of compound 1g |
| 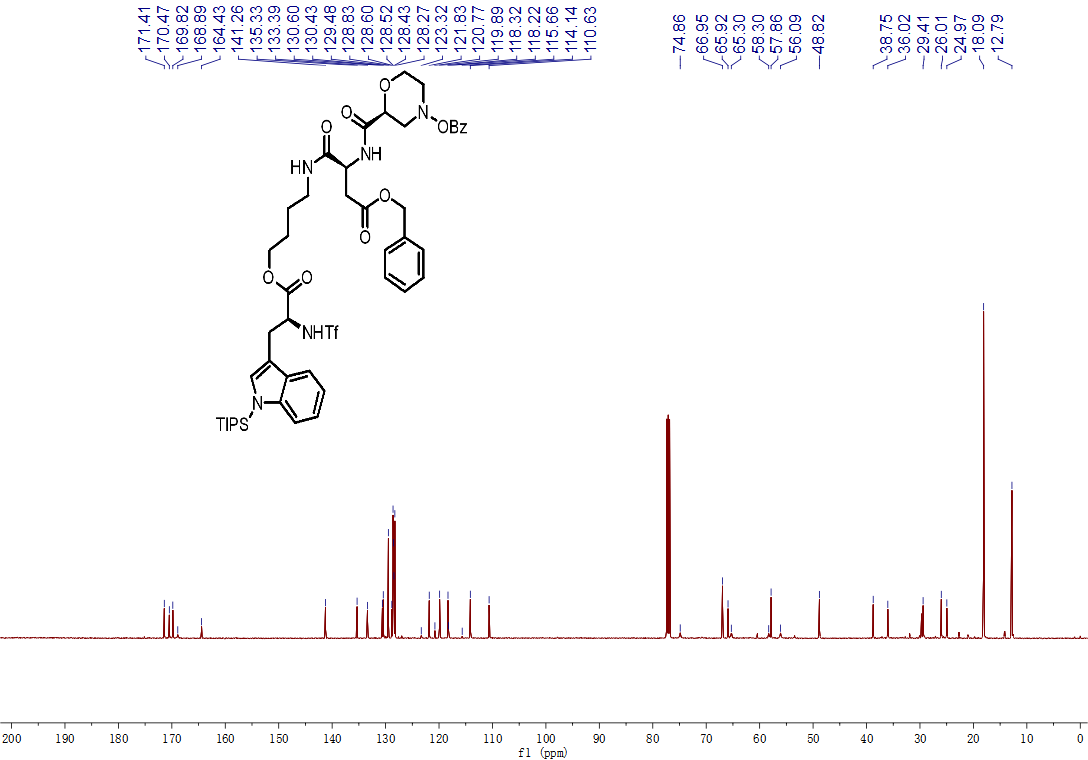  ^13^C-NMR spectrum of compound 1g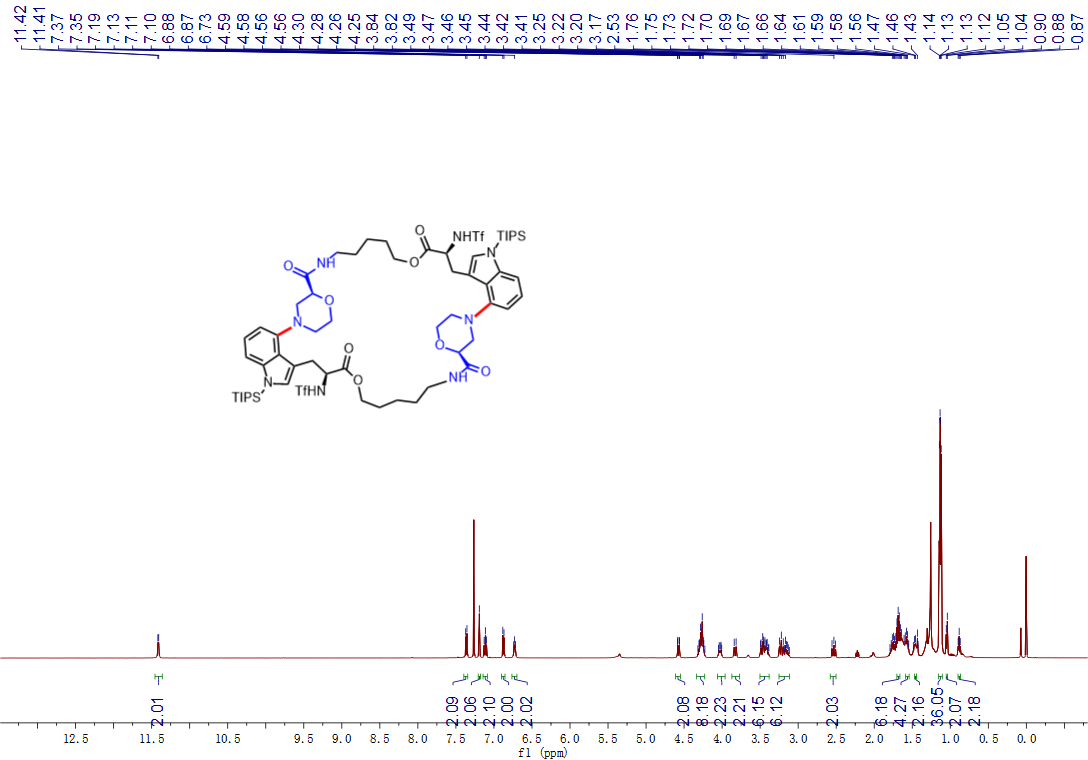  ^1^H-NMR spectrum of compound DC644 |
| 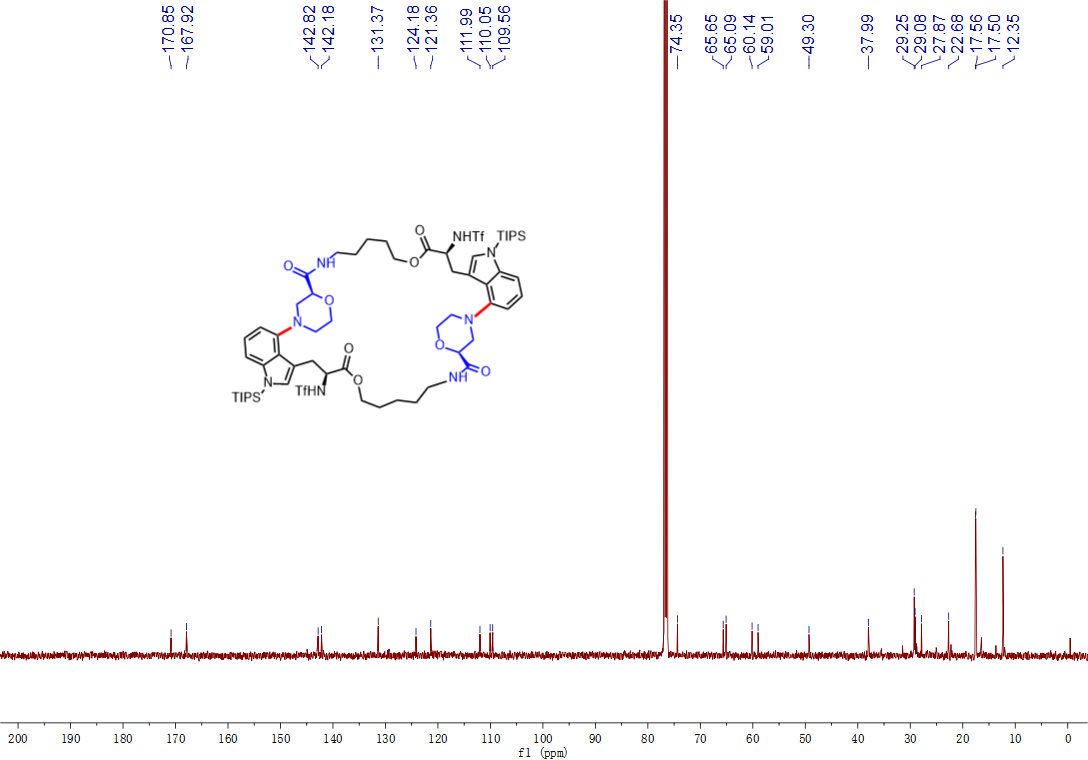  ^13^C-NMR spectrum of compound DC644 |
| 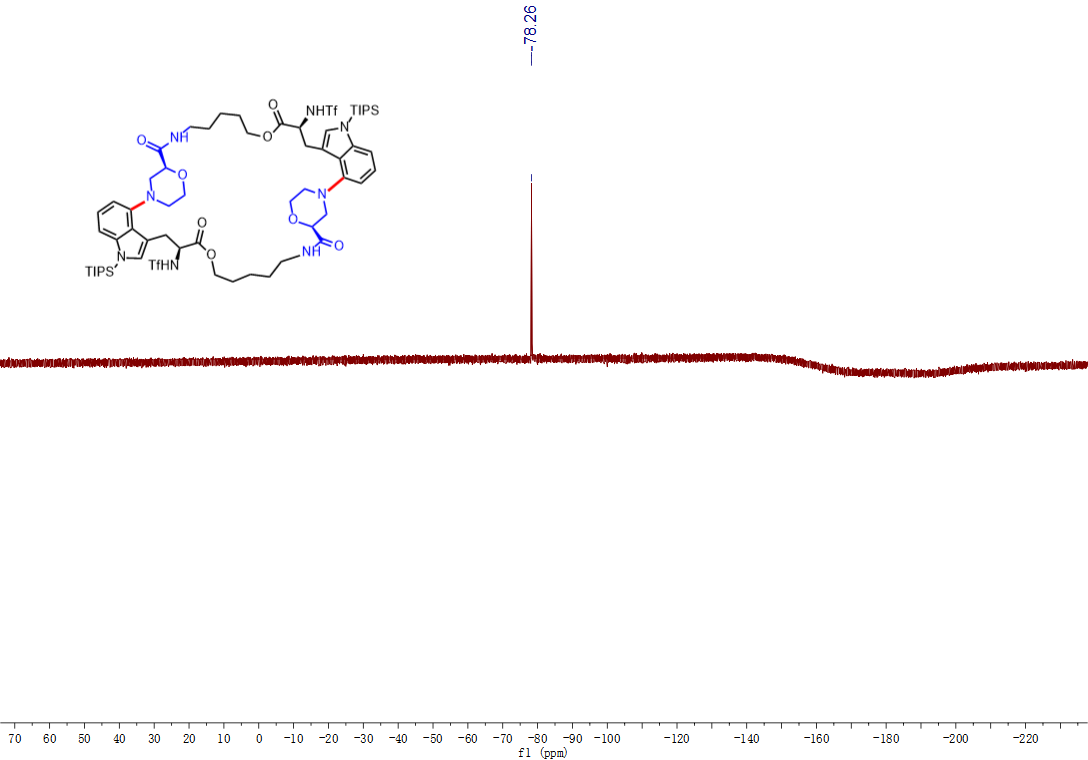  ^19^F-NMR spectrum of compound DC644 |
| 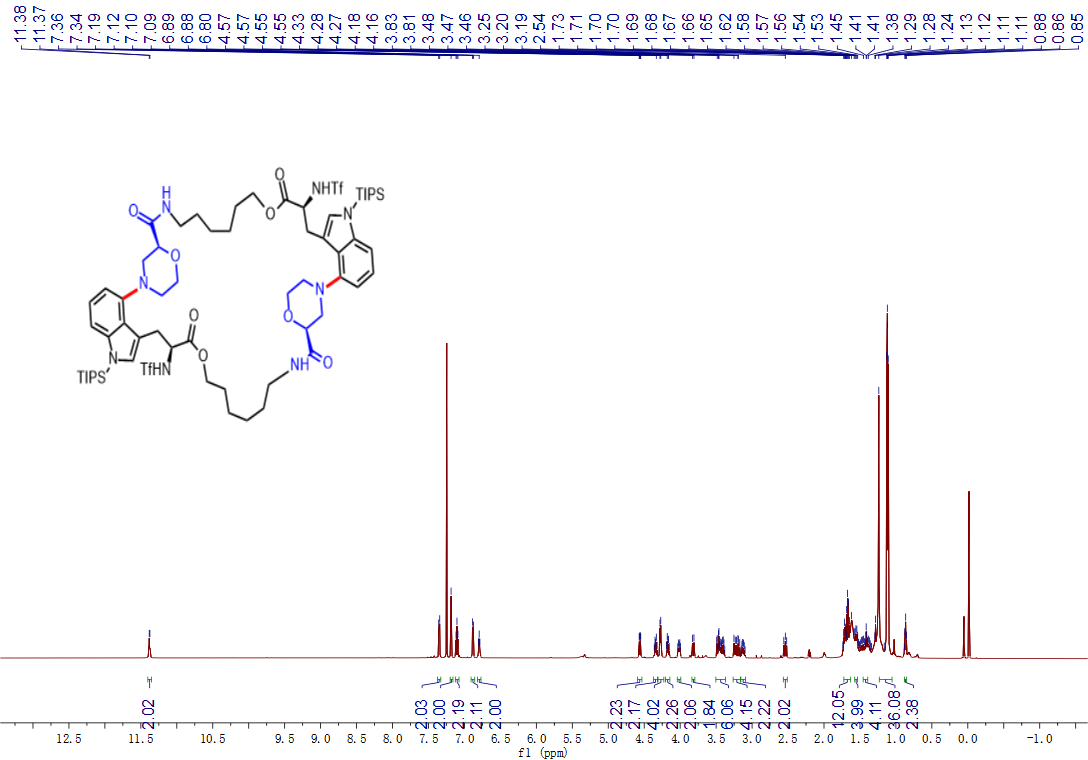  ^1^H-NMR spectrum of compound DC645 |
| 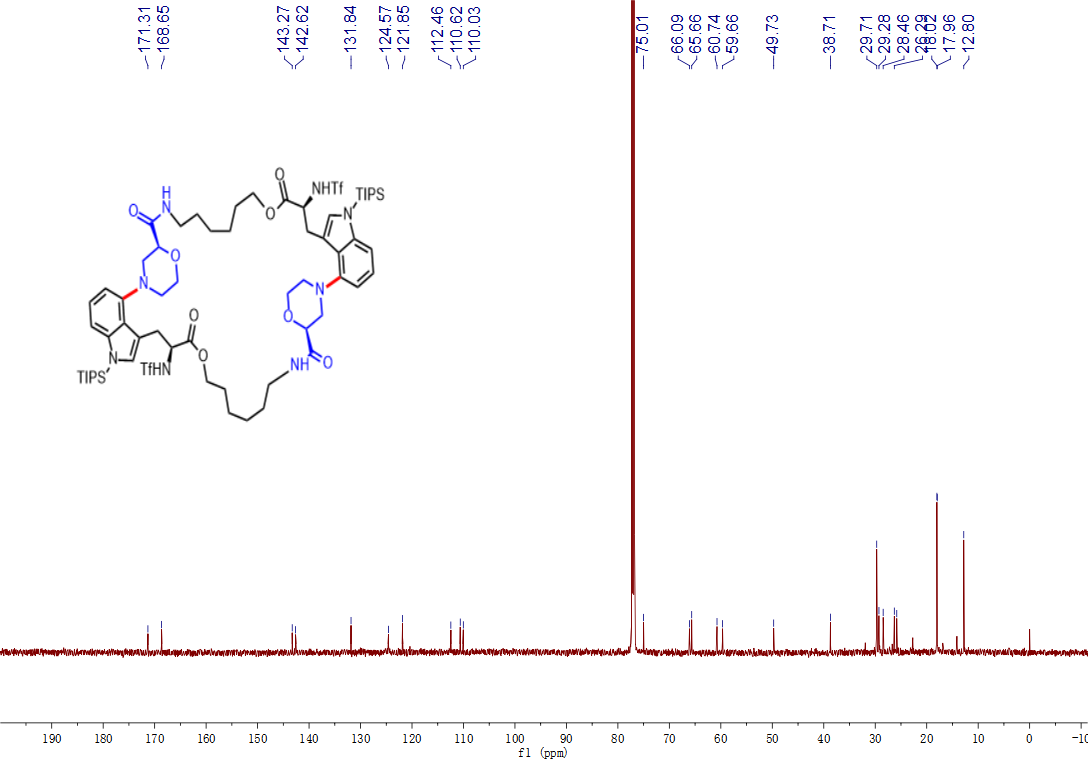  ^13^C-NMR spectrum of compound DC645 |
| 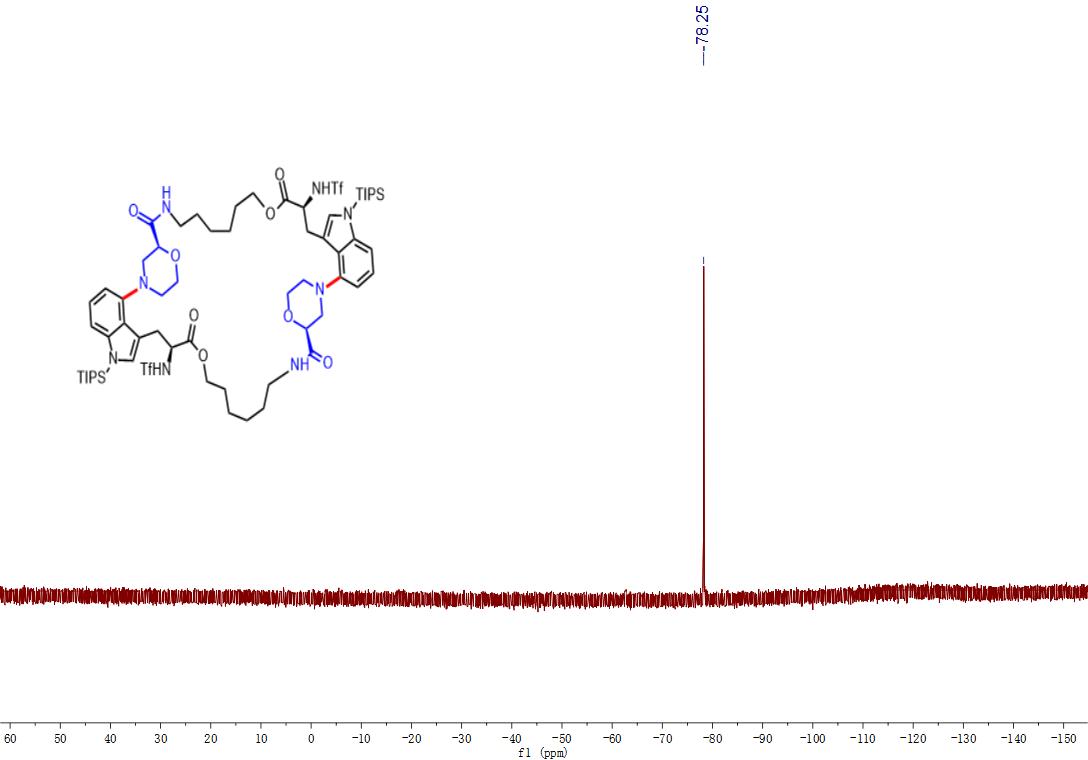  ^19^F-NMR spectrum of compound DC645 |
|  |
| 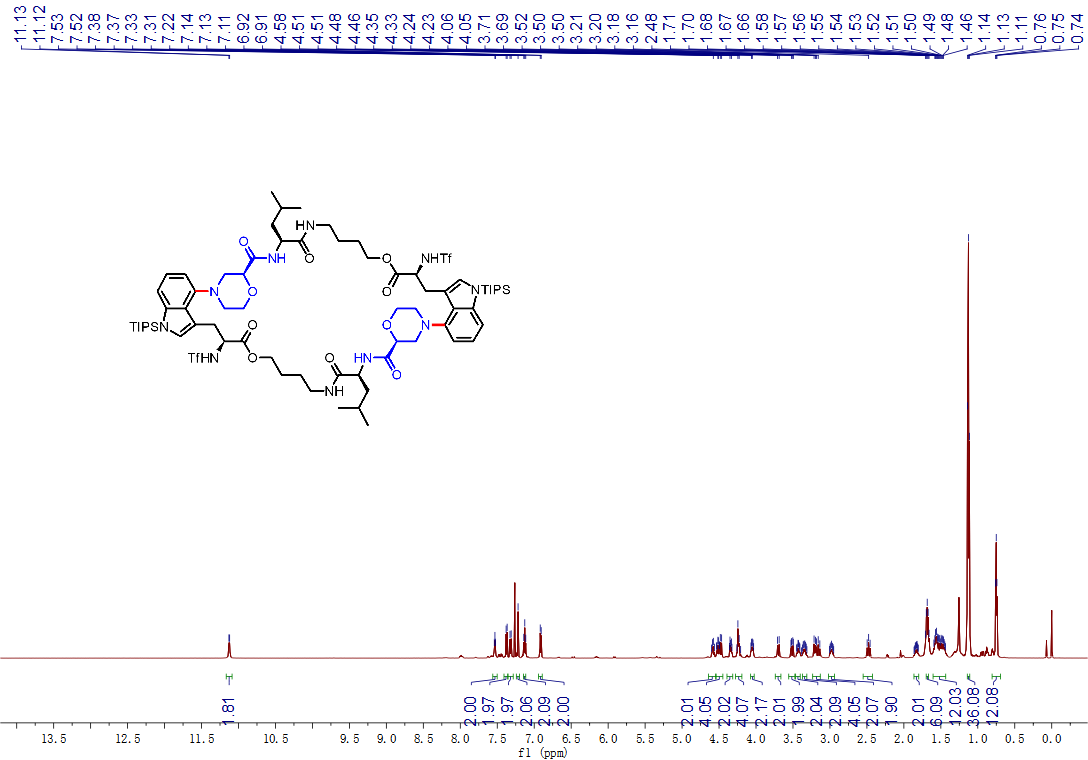  ^1^H-NMR spectrum of compound DC646 |
| 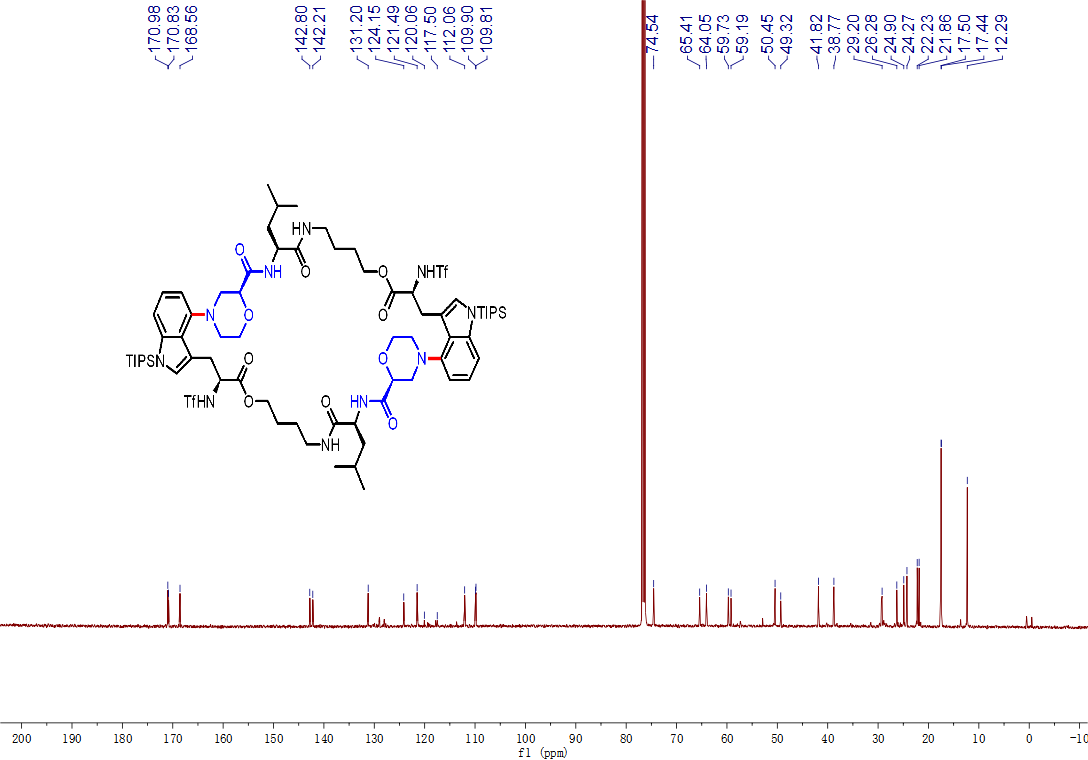  ^13^C-NMR spectrum of compound DC646 |
| 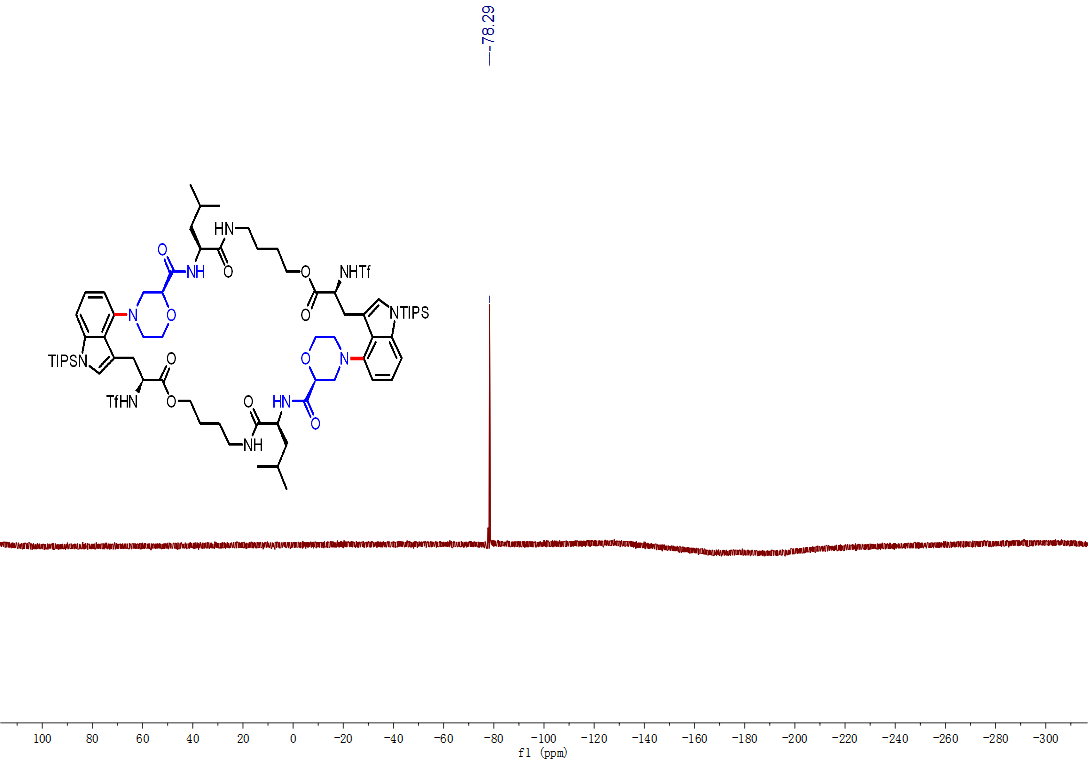  ^19^F-NMR spectrum of compound DC646 |
|  |
| 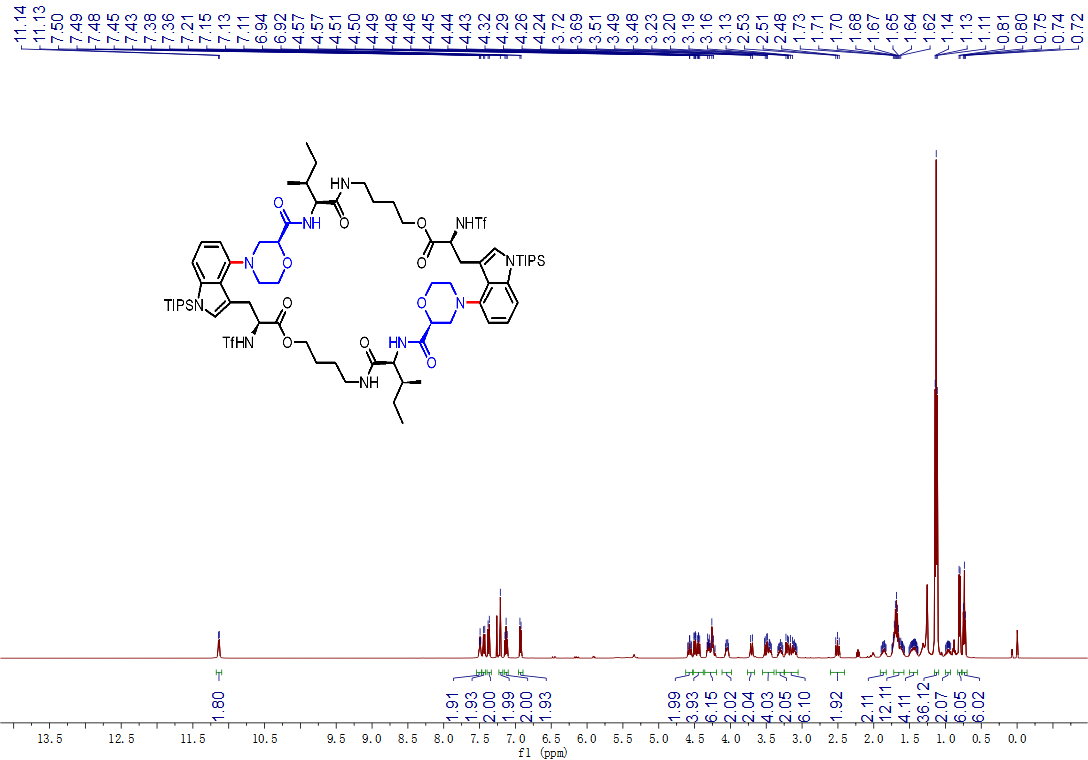  ^1^H-NMR spectrum of compound DC647 |
| 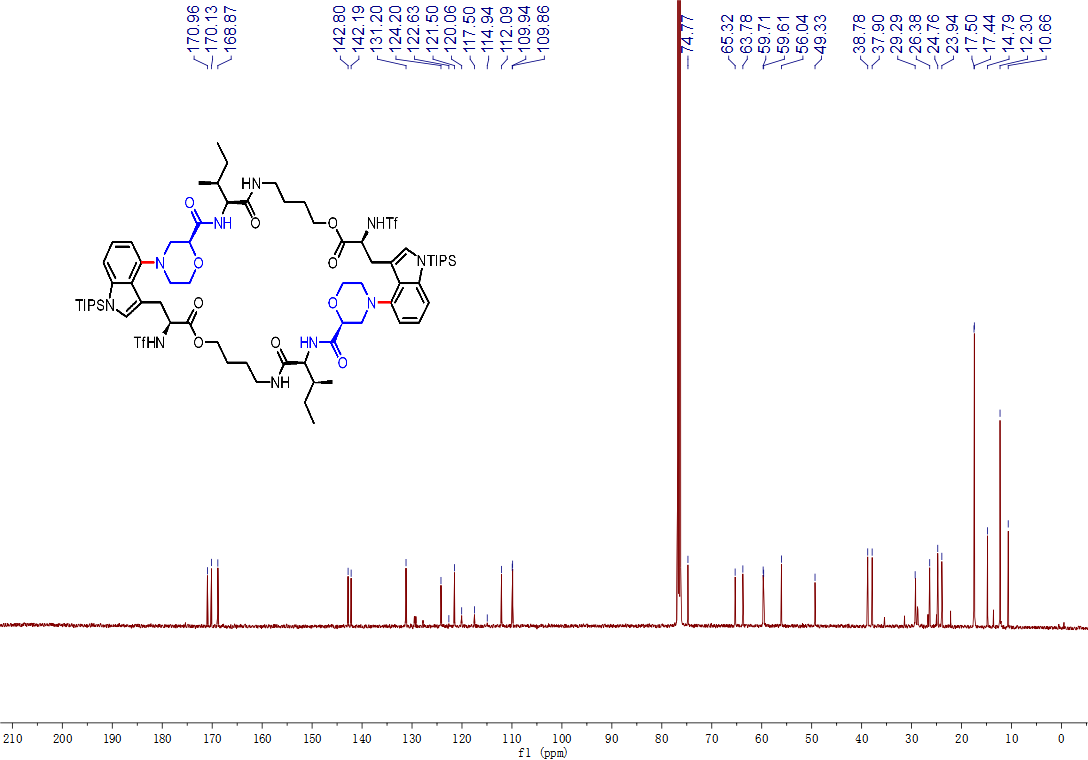  ^13^C-NMR spectrum of compound DC647 |
| 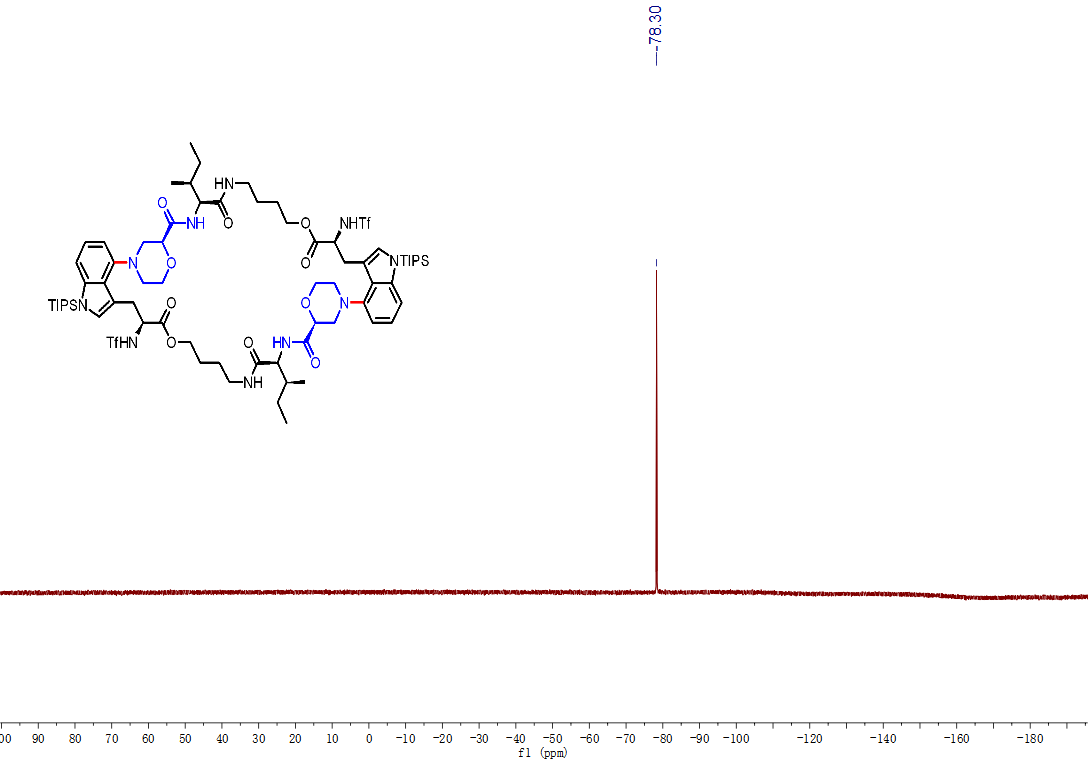  ^19^F-NMR spectrum of compound DC647 |
| 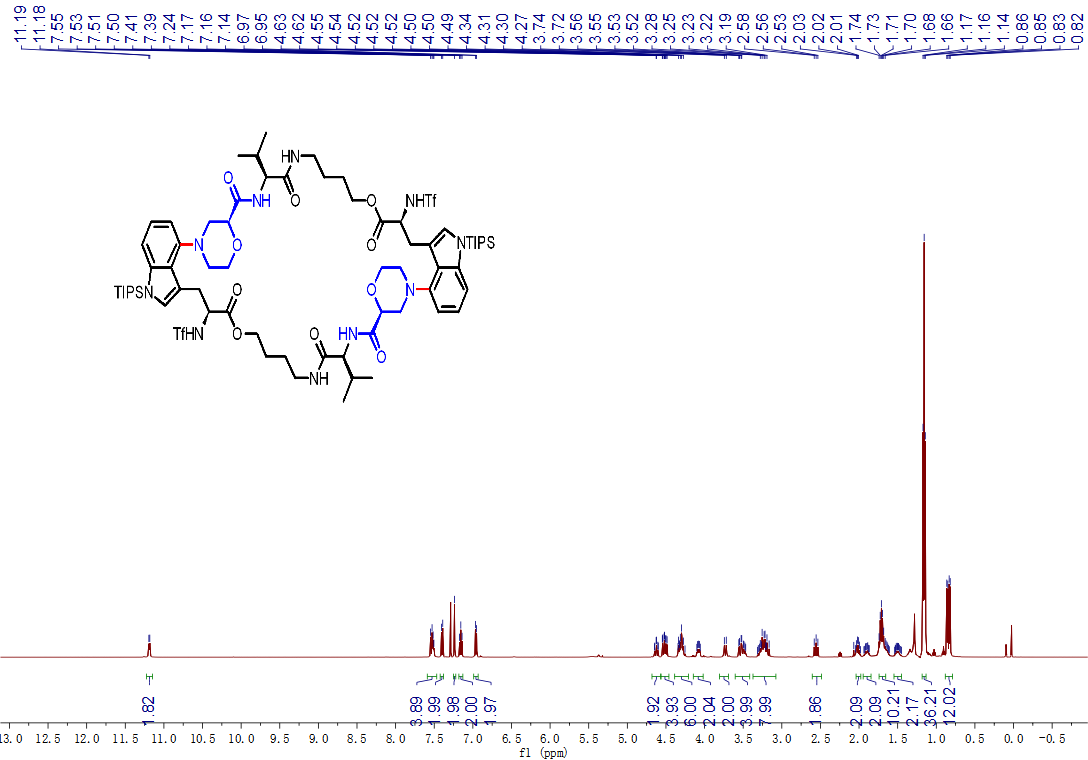  ^1^H-NMR spectrum of compound DC648 |
| 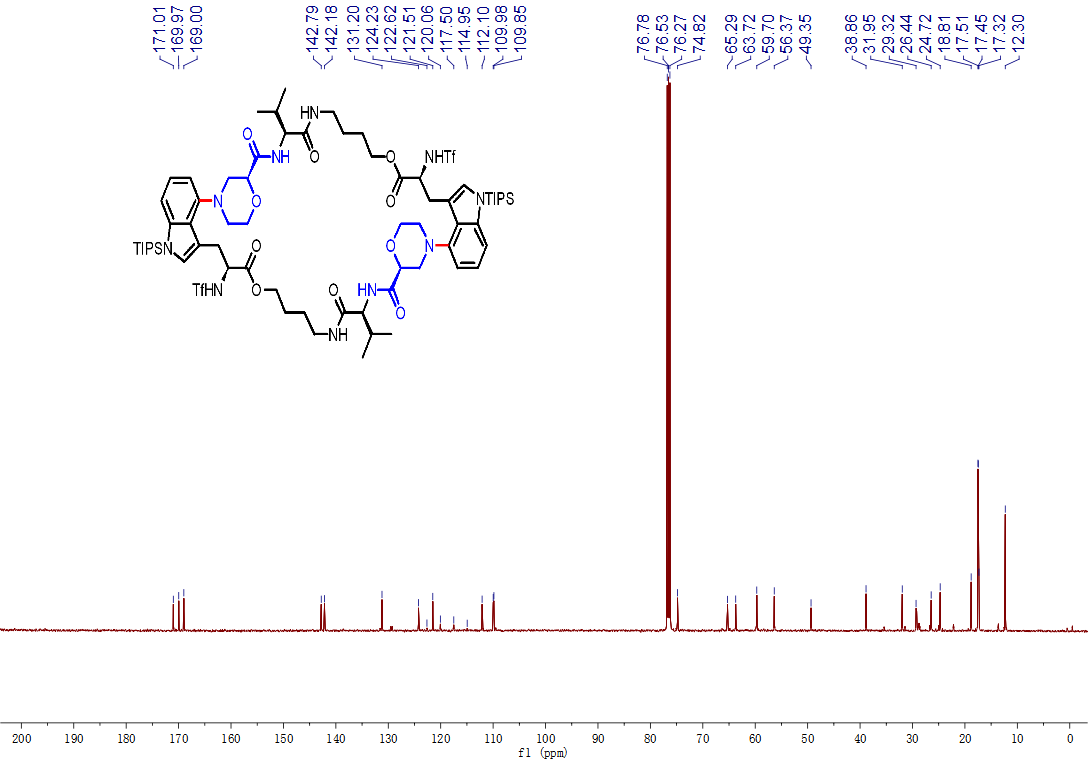  ^13^C-NMR spectrum of compound DC648 |
| 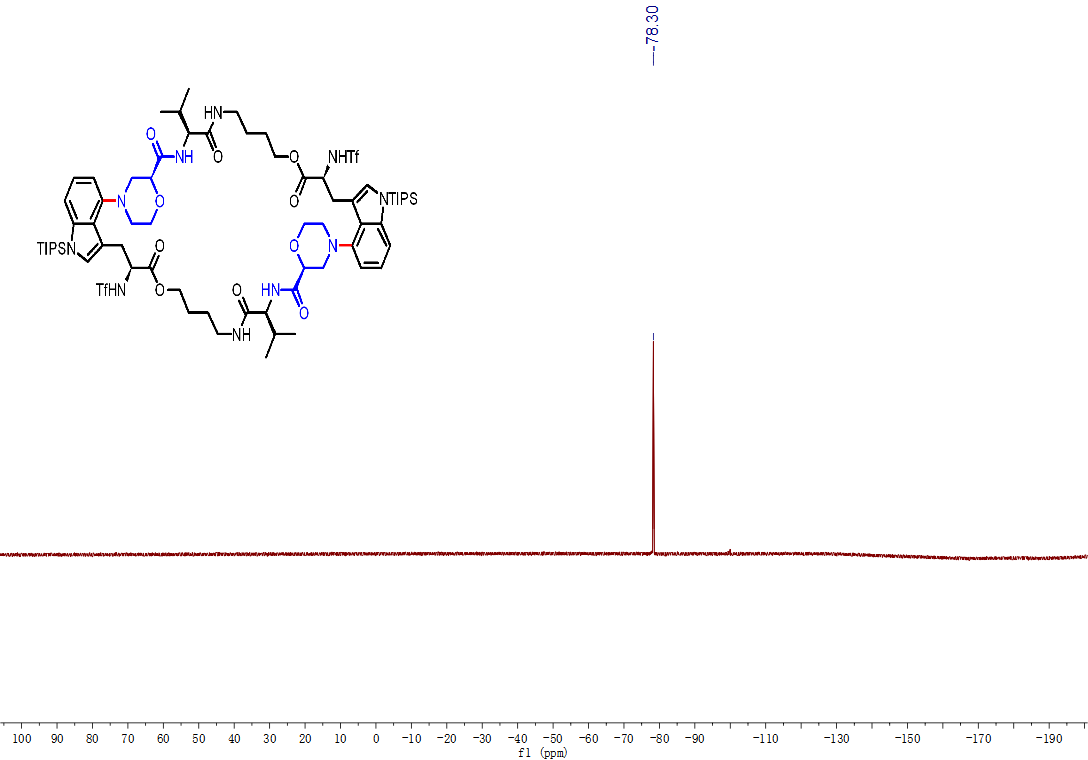  ^19^F-NMR spectrum of compound DC648 |
|  |
|  |
| 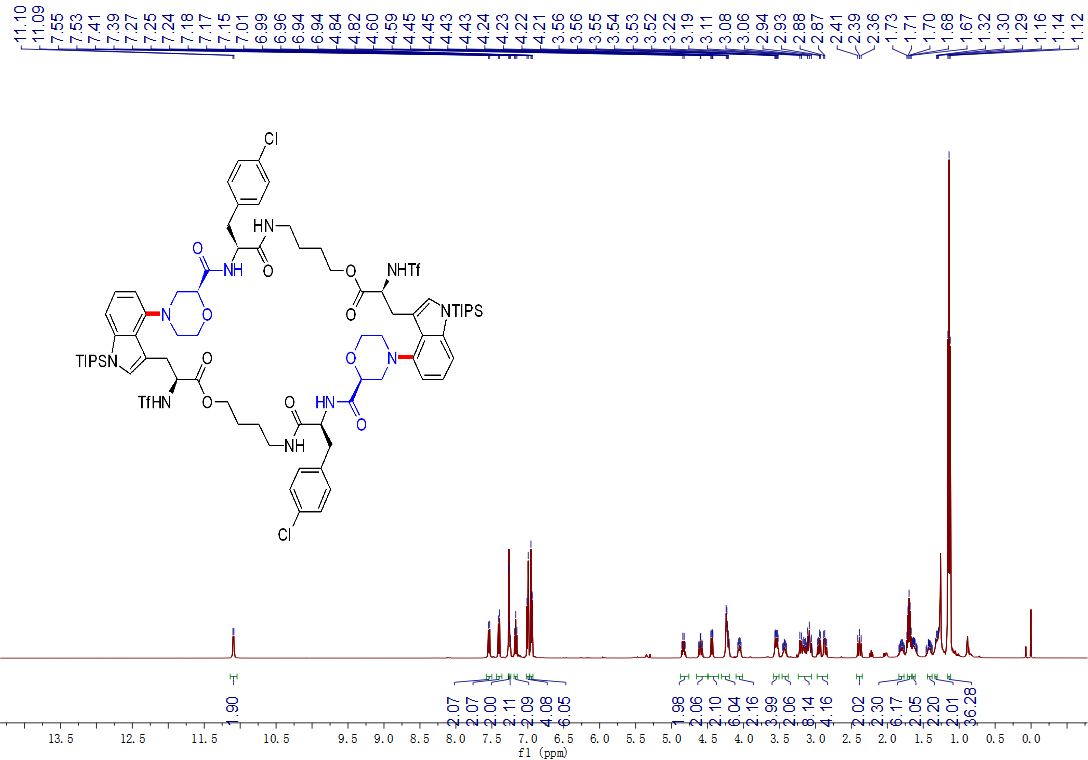  ^1^H-NMR spectrum of compound DC649 |
| 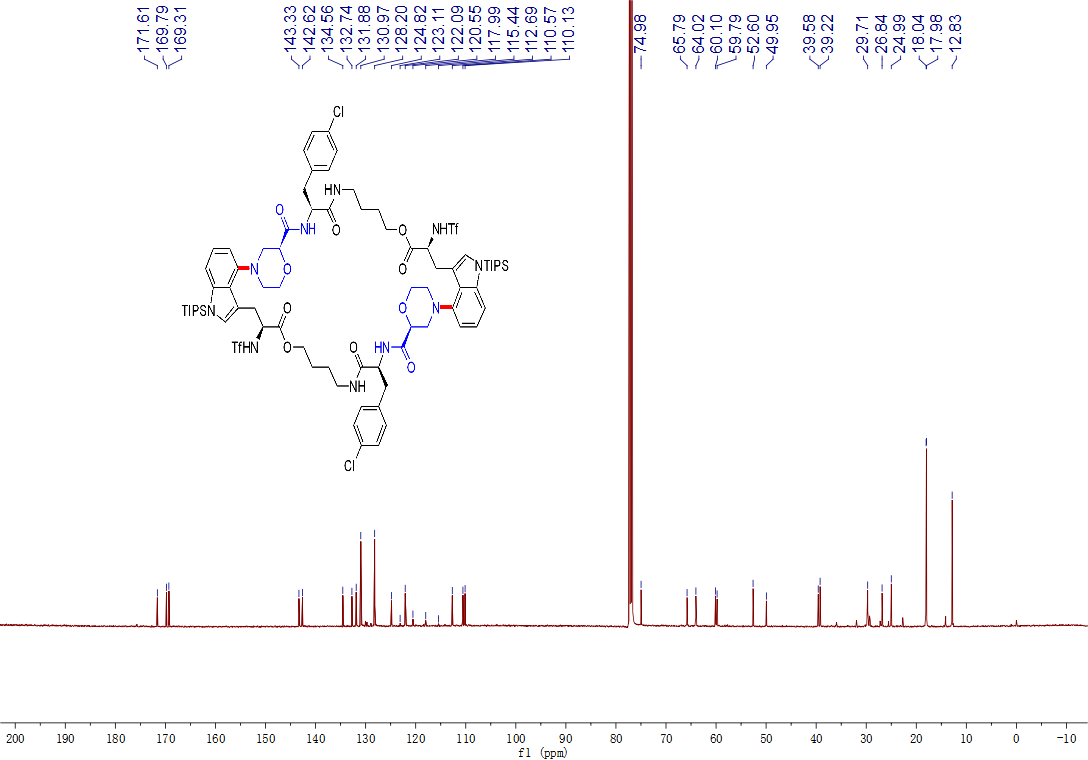  ^13^C-NMR spectrum of compound DC649 |
| 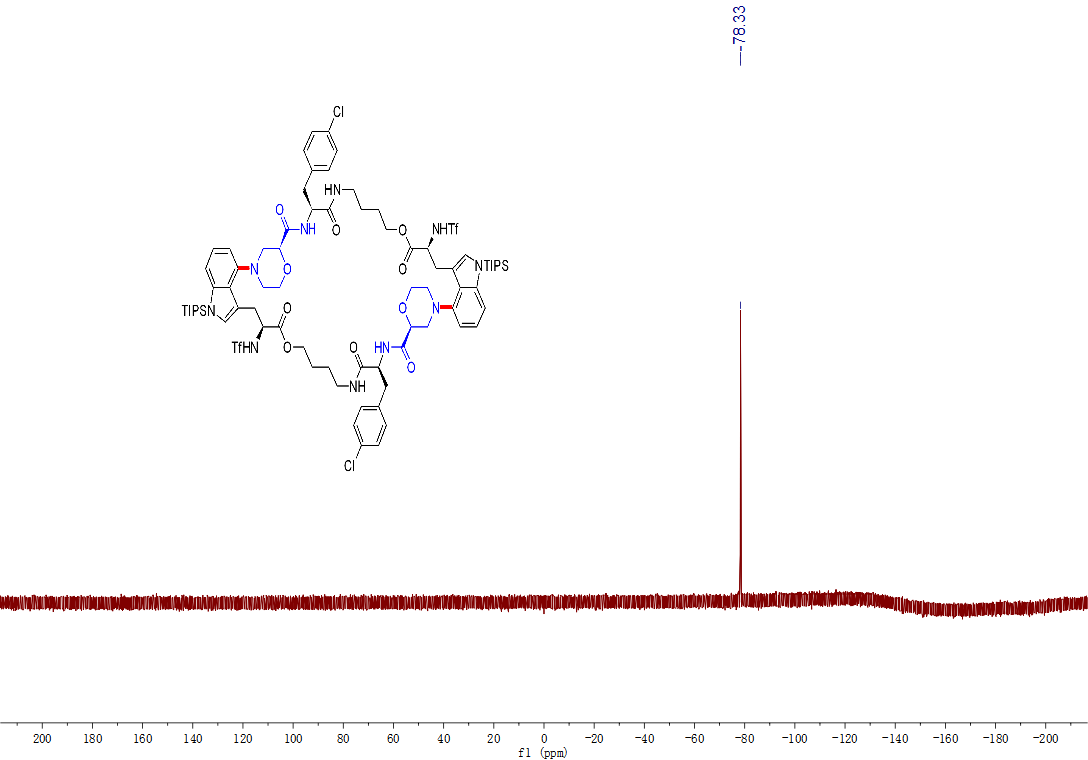  ^19^F-NMR spectrum of compound DC649  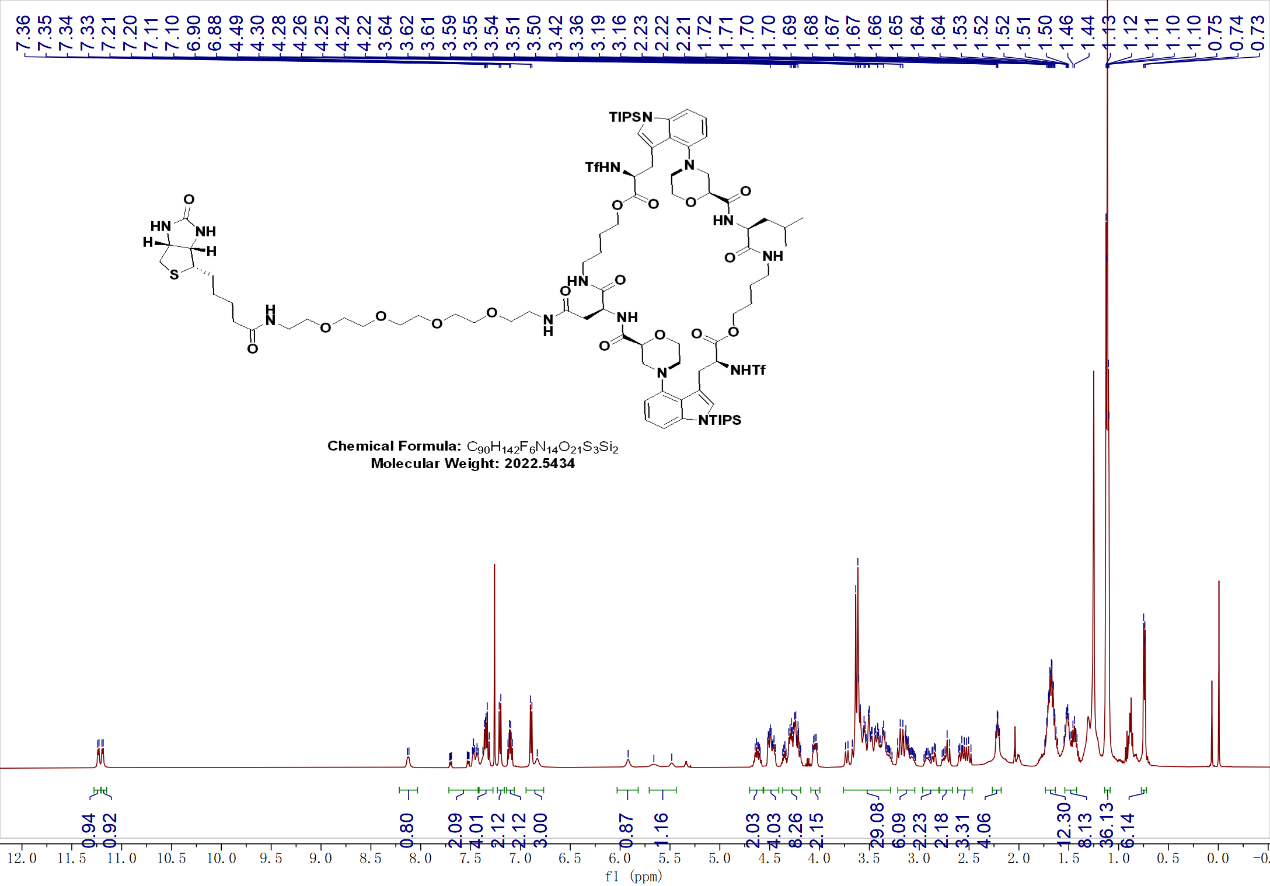  ^1^H-NMR spectrum of compound BIOTIN-DC646  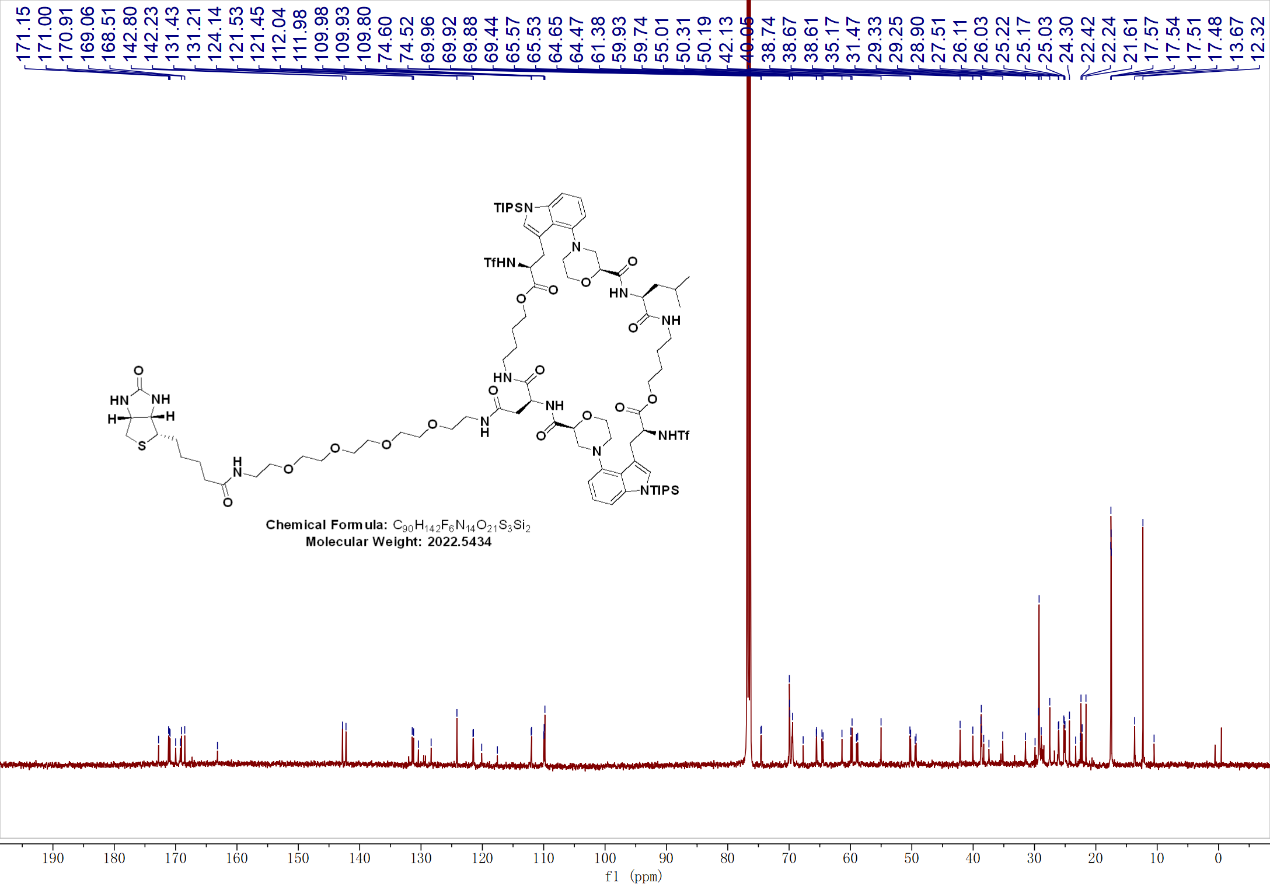  ^13^C-NMR spectrum of compound BIOTIN-DC646  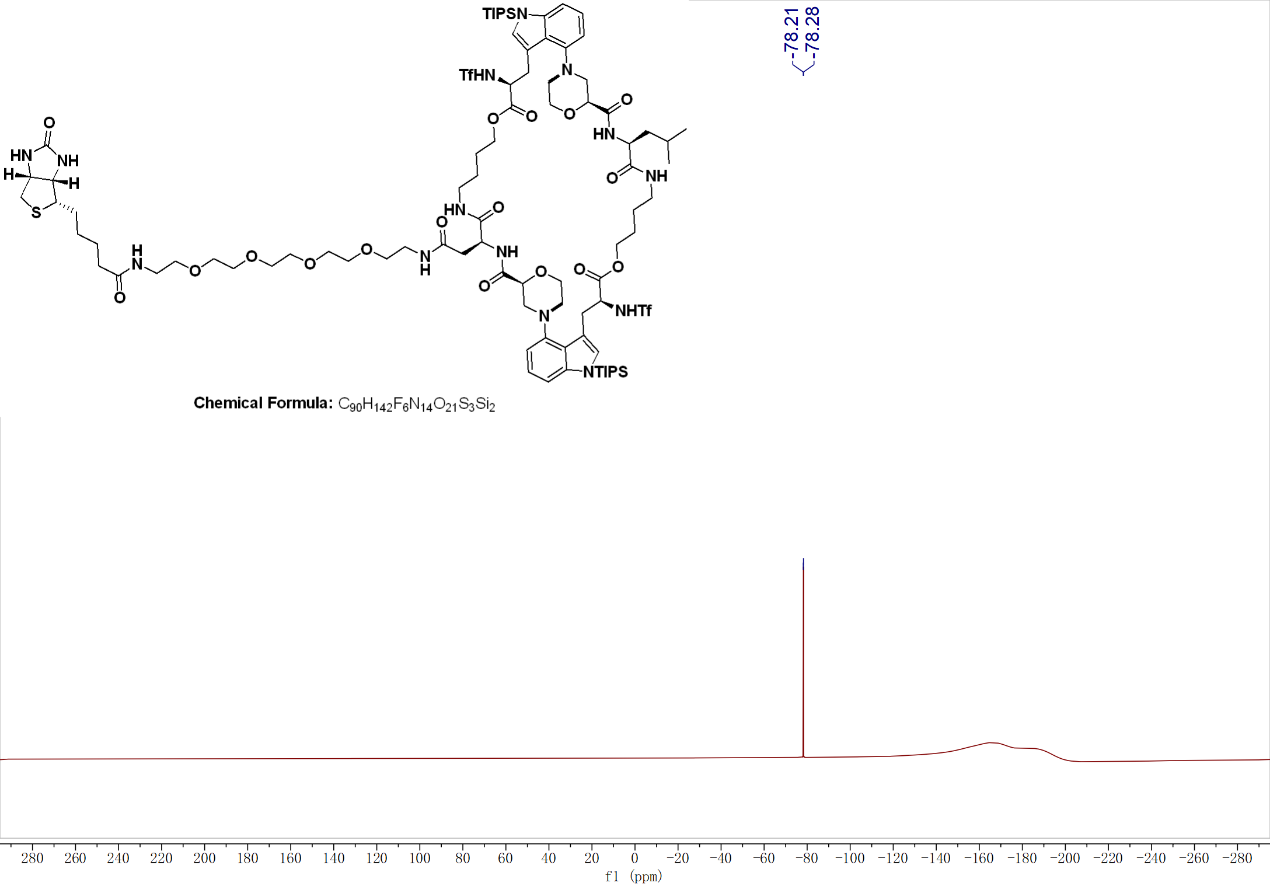  ^19^F-NMR spectrum of compound BIOTIN-DC646 |

5. HR-MS (ESI) chromatogram of compounds DC644-649 and BIOTIN-DC646

| 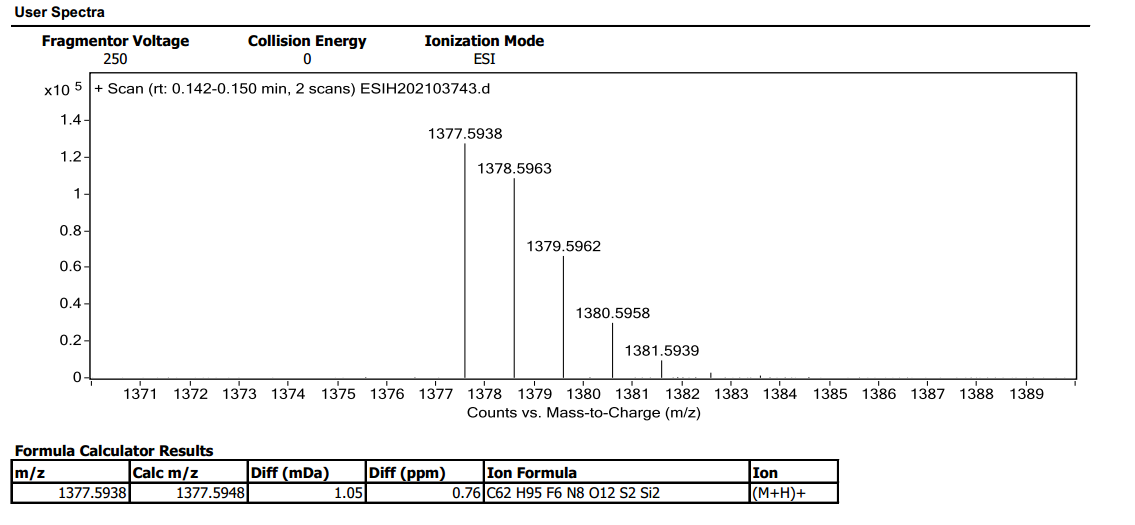  HR-MS (ESI) chromatogram of compound DC644 |
| --- |
| 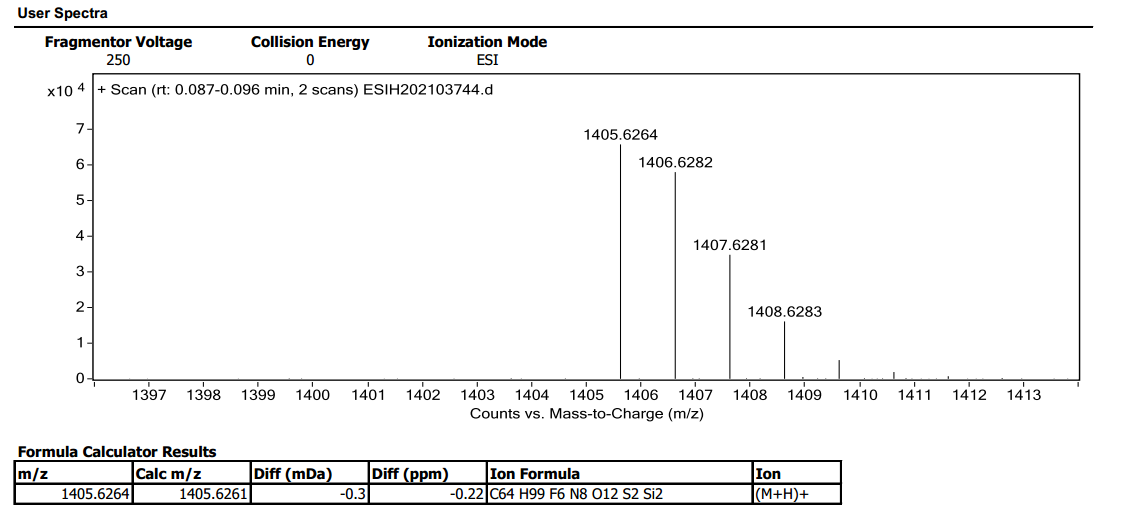  HR-MS (ESI) chromatogram of compound DC645 |
|  |
| 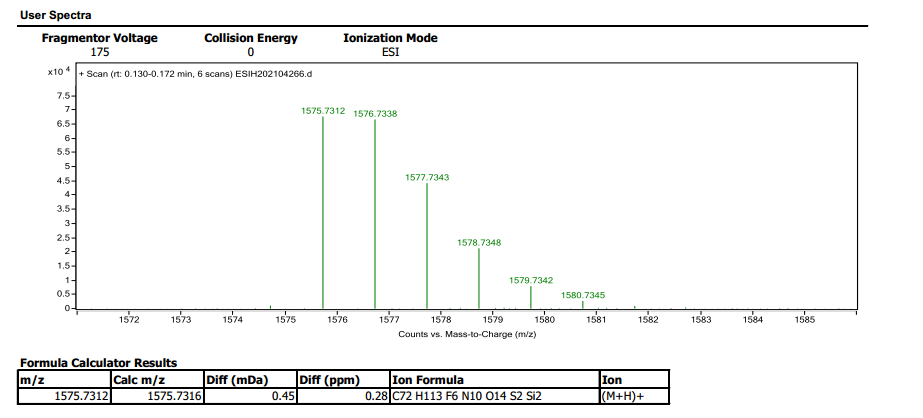  HR-MS (ESI) chromatogram of compound DC646 |
| 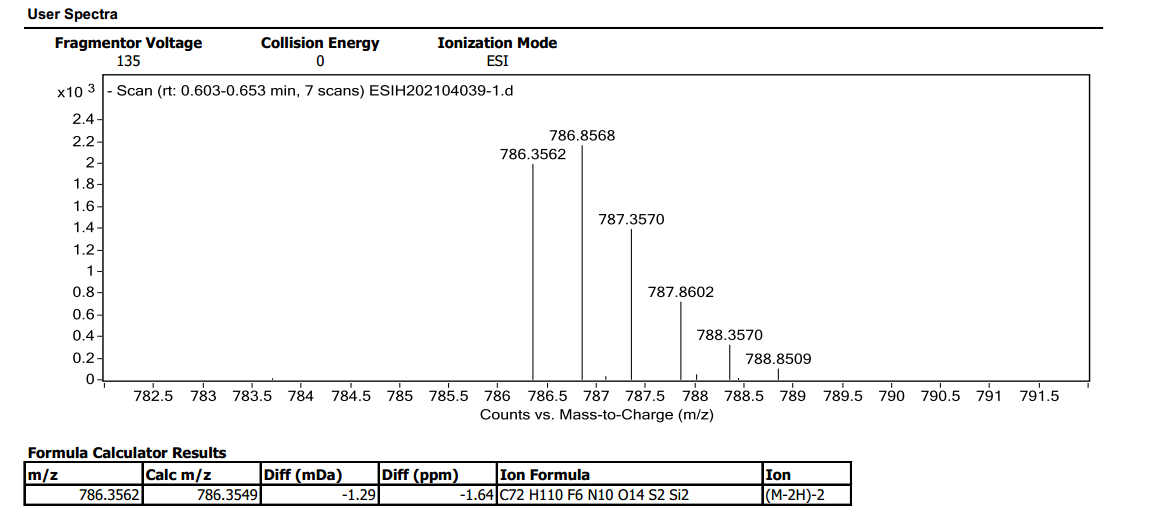  HR-MS (ESI) chromatogram of compound DC647 |
| 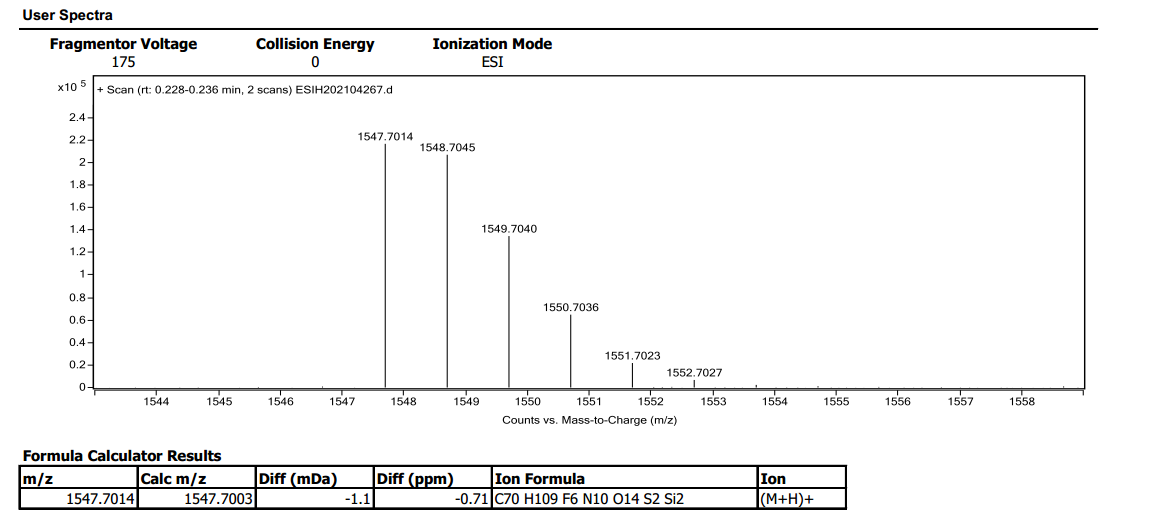  HR-MS (ESI) chromatogram of compound DC648 |
| 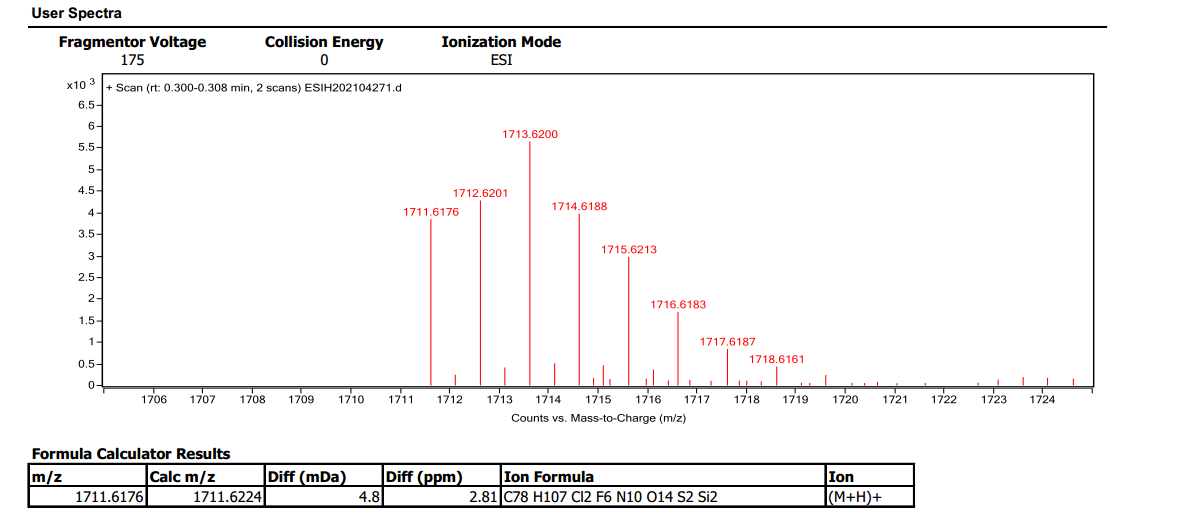  HR-MS (ESI) chromatogram of compound DC649  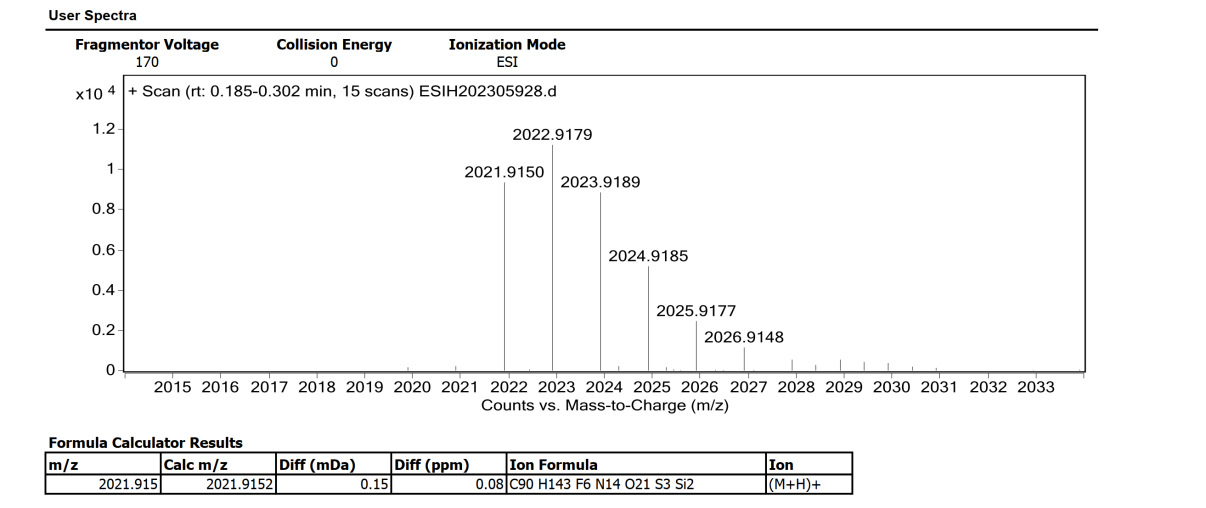 |

HR-MS (ESI) chromatogram of compound BIOTIN-DC646

6. X-ray crystallographic data of DC646

Compound DC646 (8 mg) is dissolved in 0.5 mL solvent (V_DCM_ : V_MeOH_ = 2:1), sealed with septum and the solvent is slowly volatilized at room temperature by inserting a fine needle, the crystal of compound DC646 is obtained after 24 h (Figure S5).

Supplementary Figure S5 X-Ray structure of compound DC646.

X-ray crystallographic data of DC646 are solutions at T = 170 K, C_72_H_112_F_6_N_10_O_14_S_2_Si_2_, Mr = 1576.01, monoclinic, space group: P2_1_, a = 14.813(3) Å, b = 15.887(3) Å, c = 22.189(4) Å, α = 90°, β = 105.058(13)°, γ = 90°, V = 5042.6(17) Å^3^, Z = 2. Displacement ellipsoids are drawn at the 50% probability level. CCDC 2172935 contains the supplementary crystallographic data for this paper (DC646). The data can be obtained free of charge from The Cambridge Crystallographic Data Centre via [www.ccdc.cam.ac.uk/getstructures](http://www.ccdc.cam.ac.uk/getstructures).

7. Supplementary Figures and tables


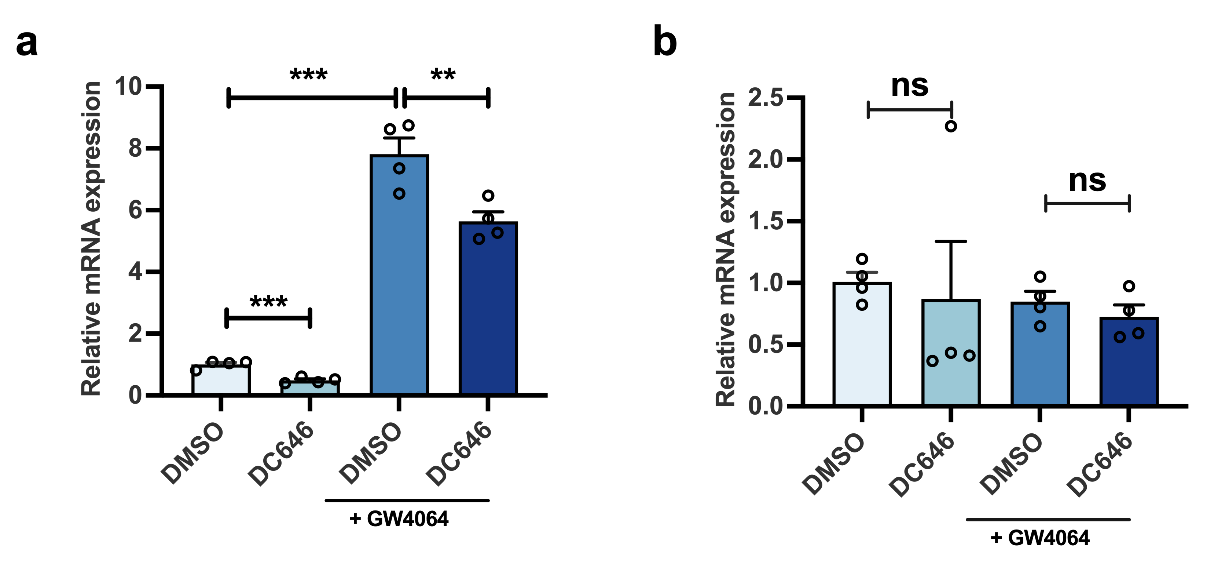


Supplementary Figure S6 FXR agonistic activity of DC646. Relative mRNA expression of *Shp* in WT (a) and *Fxr*^△IE^ (b) organoids treated with DMSO, DC646 (50 μmol/L), or GW4064 (10 μmol/L) with or without GW4064 (10 μmol/L) for 24 h (*n* = 4 in each group). Data are presented as the mean ± SEM. One-way ANOVA with Dunnett’s *post hoc* test. ^**^*P* ≤ 0.01, ^***^*P* ≤ 0.001.


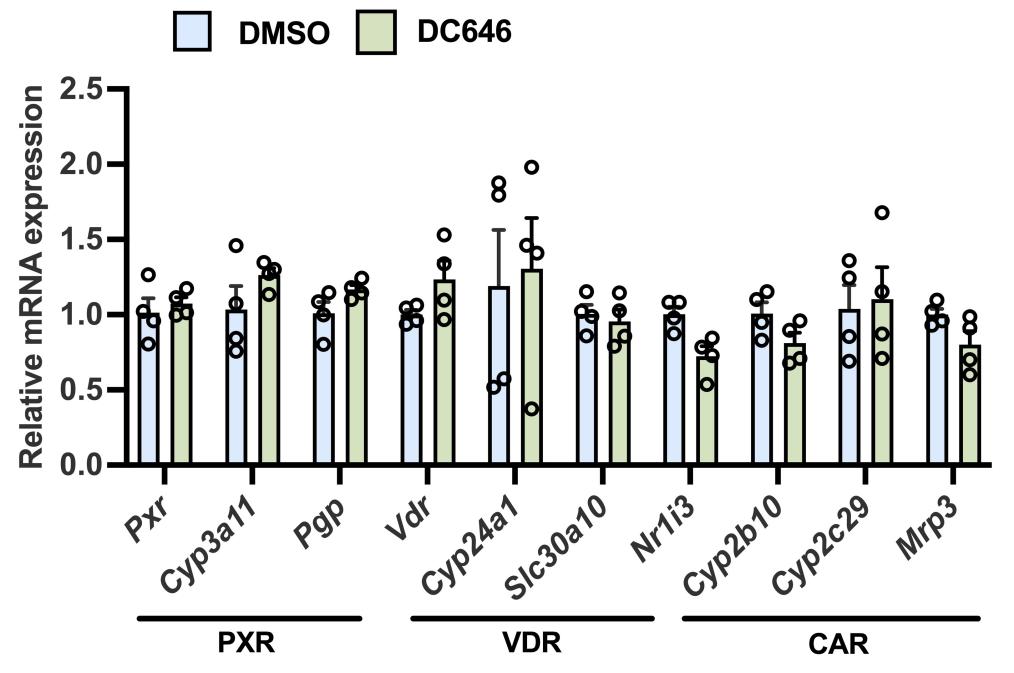


Supplementary Figure S7 Selectivity of DC646. Relative mRNA expression of *PXR*, *VDR* and *CAR*-downstream genes in organoids treated with DMSO or DC646 for 24 h (*n* = 4 in each group). Data are presented as the mean ± SEM and analyzed by unpaired *t*-test.


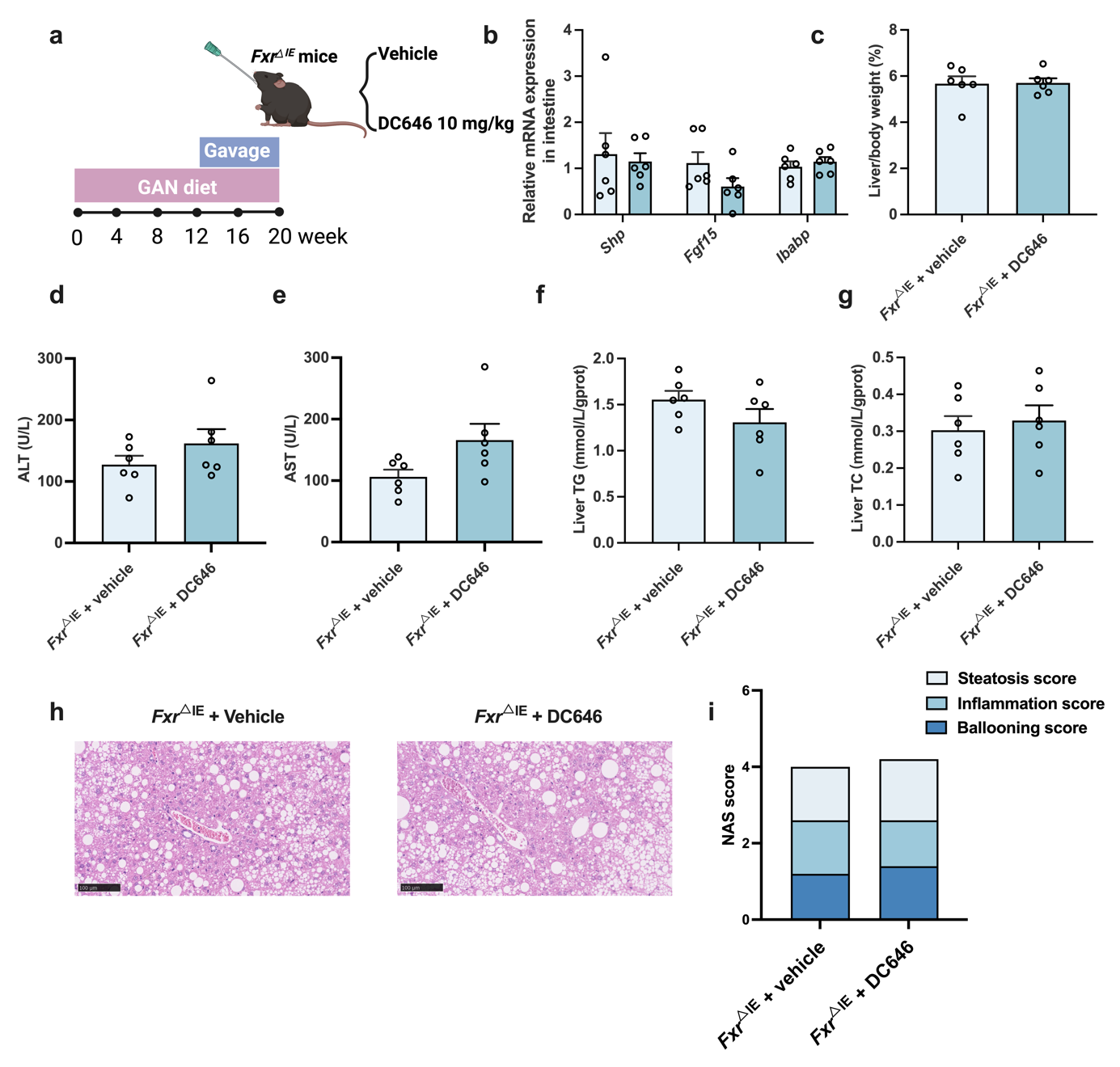


Supplementary Figure S8 The beneficial effects of DC646 on MAFLD are dependent on intestinal FXR. (a) Strategy of animal experiment. (b) Relative mRNA expression of FXR target genes in the intestine. (c) Liver to body weight ratio. (d) Serum ALT level. (e) Serum AST level. (f) Liver triglyceride contents. (g) Liver total cholesterol contents. (h) Representative H&E images. (i) NAFLD activity scoring (NAS) of each group. *n* = 6 in each group. Data are presented as the mean ± SEM and analyzed by unpaired *t*-test.


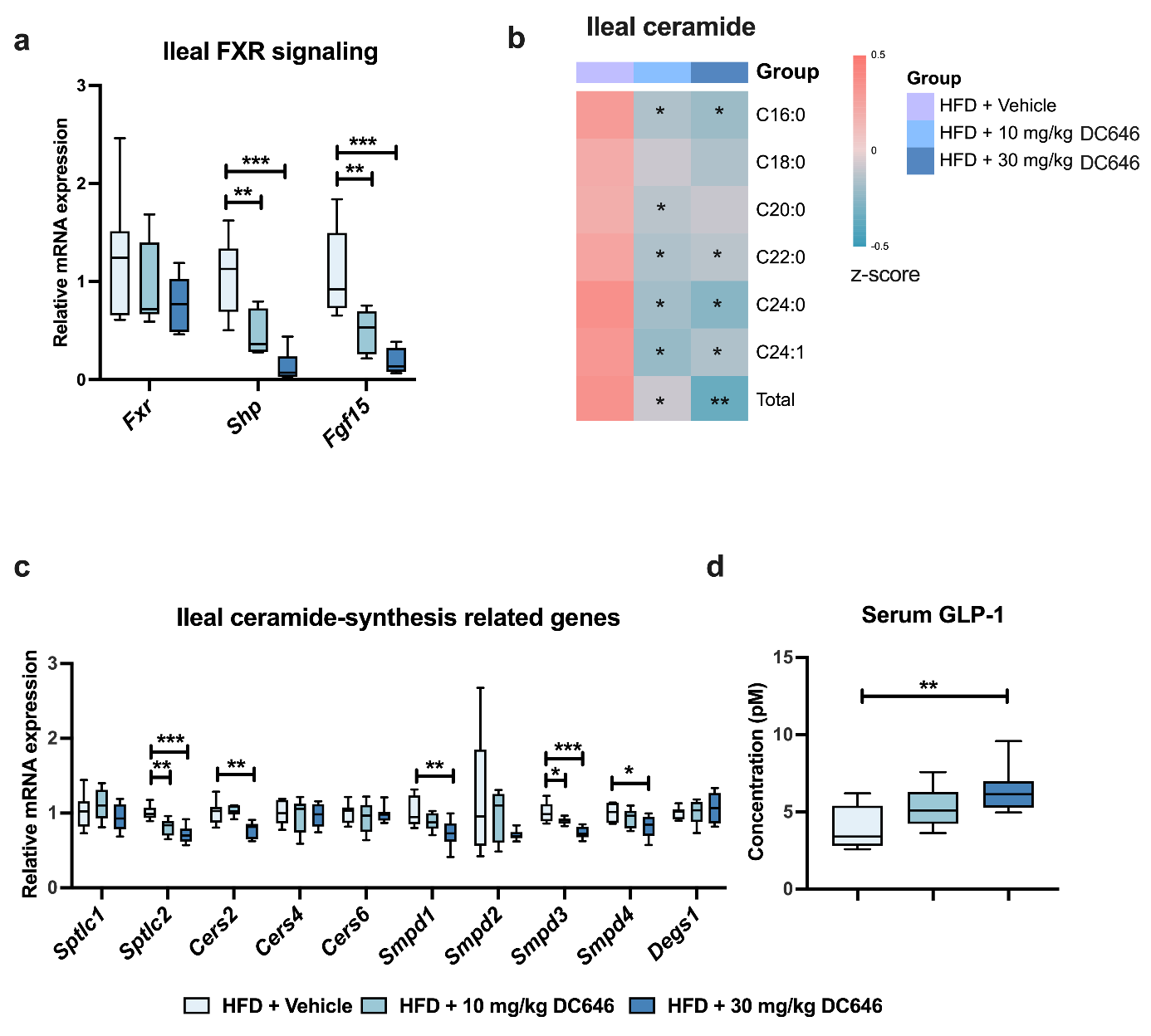


Supplementary Figure S9 DC646 antagonizes intestinal FXR, thereby reducing ceramide production and inducing GLP-1 production. (a) Relative mRNA expression of ileal FXR signaling. (b) Ileal ceramide levels (the data are normalized and centralized). (c) Relative mRNA expression of ileal ceramide-synthesis related genes. (d) Serum GLP-1 levels. HFD-fed mice were gavaged with vehicle or 10 mg/kg or 30 mg/kg DC646. *n* = 8 in each group. Data are presented as the mean ± SEM and analyzed by one-way ANOVA with Dunnett’s *post hoc* test. ^*^*P* < 0.05, ^**^*P* < 0.01, and ^***^*P* < 0.001, versus HFD + Vehicle group.


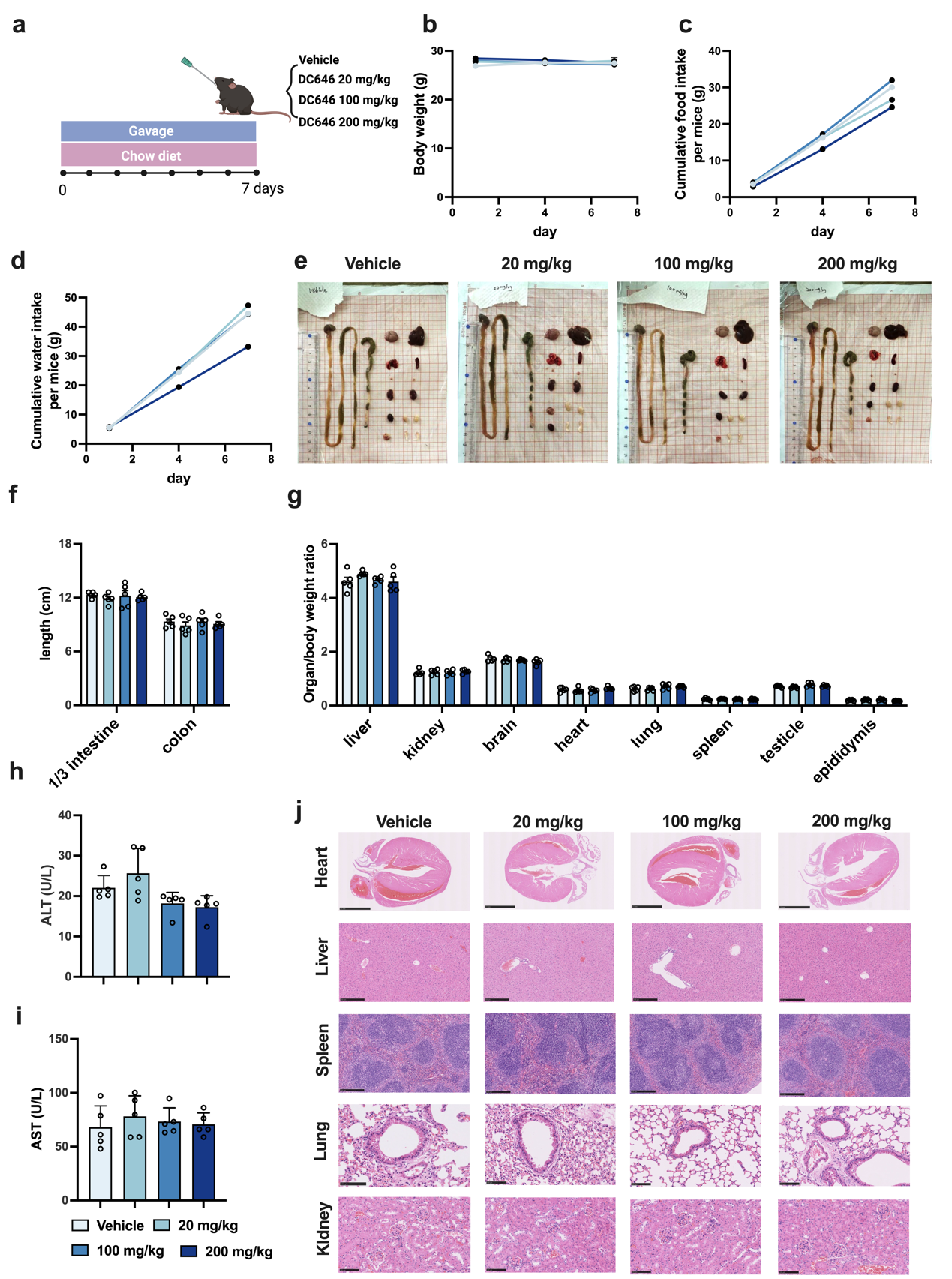


Supplementary Figure S10 Subacute toxicity test of DC646 at 20 mg/kg, 100 mg/kg, or 200 mg/kg. (a) Strategy of animal experiment. (b) Body weight. (c) Food intake. (d) Water intake. (e) Representative pictures of organ morphologies. (f) 1/3 Intestine length and colon length. (g) Organ weight. (h and i) Serum ALT (h) and AST(i) levels. (j) H&E staining of heart, liver, spleen, lung, and kidney. *n* = 5 in each group. Data are presented as the mean ± SEM and analyzed by one-way ANOVA with Dunnett’s *post hoc* test.

Supplementary Table S1 Sizes of the FXR-bound coactivators in the published experimental complex structures. The size is measured as the longest massive-atom distance within the molecule.

| PDB  code | Size (Å)* | Sequence  length | Protein  sequence | Protein  name |
| --- | --- | --- | --- | --- |
| 3RVF | 20.0 | 11 | DHQLLRYLLDK | Nuclear receptor coactivator 1 |
| 3DCT | 20.2 | 11 | DHQLLRYLLDK |  |
| 3RUT | 20.8 | 11 | DHQLLRYLLDK |  |
| 3BEJ | 24.4 | 12 | TERHKILHRLLQ |  |
| 5Z12 | 20.5 | 9 | HKILHRLLQ | Nuclear receptor coactivator 2 |
| 5ICK | 20.9 | 11 | NALLRYLLDKD |  |
| 5IAW | 21.5 | 10 | NALLRYLLDK |  |
| 7D42 | 22.4 | 11 | ENALLRYLLDK |  |
| 7VUE | 22.5 | 11 | ENALLRYLLDK |  |
| 5WZX | 23.1 | 12 | ENALLRYLLDKD |  |
| 6HL1 | 26.6 | 13 | KENALLRYLLDKD |  |

Supplementary Table S2 Predicted sizes of the peptide candidates. The size is measured as the longest massive-atom distance within the molecule.

| Peptide | Chemical structure | Size (Å) |
| --- | --- | --- |
| SJN10 |  | 13.8 ± 0.1 |
| SJN18 |  | 12.6 ± 0.0 |
| HT14 |  | 16.8 ± 0.1 |
| HT15 |  | 15.3 ± 0.1 |
| HT18-2 |  | 15.4 ± 0.1 |
| DC644 |  | 22.6 ± 3.2 |

Supplementary Table S3 Distribution of DC646 in the liver, intestine, and serum.

| Tissue | Time | DC646 |
| --- | --- | --- |
| Liver  (ng/g) | 1 h | BQL^$^ |
|  | 4 h | BQL |
|  | 8 h | BQL |
|  | 24 h | BQL |
| Intestine (ng/g) | 1 h | 6350.00 |
|  | 4 h | 435.00 |
|  | 8 h | 72.00 |
|  | 24 h | BQL^$^ |
| Serum  (ng/mL) | 1 h | BQL^&^ |
|  | 4 h | BQL |
|  | 8 h | BQL |
|  | 24 h | BQL |

^$^Below the quantization limit (curve range: 50.0−62500 ng/g)

^&^Below the quantization limit (curve range: 20.0−6000 ng/mL)

Supplementary Table S4 Selectivity of DC646 on PPARs by cell-based reporter assays.

| Compd. | Con. （μmol/L） | PPARα | | PPARδ | | PPARγ | |
| --- | --- | --- | --- | --- | --- | --- | --- |
|  |  | Inhibition% | SD% | Inhibition% | SD% | Inhibition% | SD% |
| DC646 | 10 | −1.3 | 17.6 | 18.9 | 8.1 | 14.0 | 4.3 |

Inhibition% means inhibition percentage of agonist-induced receptor activation.

Supplementary Table S5 Sequences of primers used for quantitative real-time PCR.

| Gene | Primer sequence |
| --- | --- |
| *Fxr* | F: 5'-TGGGCTCCGAATCCTCTTAGA-3' |
|  | R: 5'-TGGTCCTCAAATAAGATCCTTGG-3' |
| *Shp* | F: 5'-TCTGCAGGTCGTCCGACTATTC-3' |
|  | R: 5'-AGGCAGTGGCTGTGAGATGC-3' |
| *Fgf15* | F: 5'-GCCATCAAGGACGTCAGCA-3' |
|  | R: 5'-CTTCCTCCGAGTAGCGAATCAG-3' |
| *Cyp7a1* | F: 5'-AACAACCTGCCAGTACTAGATAGC-3' |
|  | R: 5'-GTGTAGAGTGAAGTCCTCCTTAGC-3' |
| *Cyp8b1* | F: 5'-CTAGGGCCTAAAGGTTCGAGT-3' |
|  | R: 5'-GTAGCCGAATAAGCTCAGGAAG-3' |
| *Pxr* | F: 5'-GGAAGAGCCCATCAACGTAG-3' |
|  | R: 5'-TGCATCCTTCACACGTCAT-3' |
| *Cyp3a11* | F: 5'-CGCCTCTCCTTGCTGTCACA-3' |
|  | R: 5'-CTTTGCCTTCTGCCTCAAGT-3' |
| *P-gp* | F: 5'-CAGCAGTCAGTGTGCTTACAA-3' |
|  | R: 5'-ATGGCTCTTTTATCGGCCTCA-3' |
| *Vdr* | F: 5'-CCGGAATGTGCCTCGGATCTG-3' |
|  | R: 5'-ATGCCAATGTCCACGCAGCG-3' |
| *Cyp24a1* | F: 5'-CTGCCCCATTGACAAAAGGC-3' |
|  | R: 5'-CTCACCGTCGGTCATCAGC-3' |
| *Slc30a10* | F: 5'-TGTCAGACCTGATCTCGCTGT-3' |
|  | R: 5'-GCCTCCACGAAGATGGTGAA-3' |
| *Nr1i3* | F: 5'-ATATGGGCCGAGGAACTGTGT-3' |
|  | R: 5'-GGCGTGGAAATGATAGCCTGT-3' |
| *Cyp2b10* | F: 5'-AAAGTCCCGTGGCAACTTCC-3' |
|  | R: 5'-TTGGCTCAACGACAGCAACT-3' |
| *Cyp2c29* | F: 5'-ATCTGGTCGTGTTCCTAGCG-3' |
|  | R: 5'-CAGTAGGCTTTGAGCCCAAATA-3' |
| *Mrp3* | F: 5'-CTGGGTCCCCTGCATCTAC-3' |
|  | R: 5'-GCCGTCTTGAGCCTGGATAAC-3' |
| *Elovl6* | F: 5'- GAAAAGCAGTTCAACGAGAACG -3' |
|  | R: 5'- AGATGCCGACCACCAAAGATA -3' |
| *Acsl1* | F: 5'- CGATGGCTGTTGGACTTTGC -3' |
|  | R: 5'- CACCCAGGCTCGACTGTATC -3' |
| *Scd1* | F: 5'- TTCTTGCGATACACTCTGGTGC -3' |
|  | R: 5'- CGGGATTGAATGTTCTTGTCGT -3' |
| *Cd36* | F: 5'- AGATGACGTGGCAAAGAACAG -3' |
|  | R: 5'- CCTTGGCTAGATAACGAACTCTG -3' |
| *Fabp1* | F: 5'- ATGAACTTCTCCGGCAAGTACC -3' |
|  | R: 5'- CTGACACCCCCTTGA TGTCC -3' |
| *Fabp4* | F: 5'- AAGGTGAAGAGCATCATAACCCT -3' |
|  | R: 5'- TCACGCCTTTCATAACACATTCC -3' |
| *Apoe* | F: 5'- CTGACAGGATGCCTAGCCG-3' |
|  | F: 5'- CGCAGGTAATCCCAGAAGC-3' |
| *Cyp4a10* | F: 5'- TTCCCTGATGGACGCTCTTTA-3' |
|  | F: 5'- GCAAACCTGGAAGGGTCAAAC-3' |
| *Cpt1b* | F: 5'- GCACACCAGGCAGTAGCTTT-3' |
|  | F: 5'- CAGGAGTTGATTCCAGACAGGTA-3' |
| *Sptlc1* | F: 5'- CGAGGGTTCTATGGCACATT -3' |
|  | R: 5'- GGTGGAGAAGCCATACGAGT -3' |
| *Sptlc2* | F: 5'- TCACCTCCATGAAGTGCATC -3' |
|  | R: 5'- CAGGCGTCTCCTGAAATACC -3' |
| *Degs1* | F: 5'- AATGGGTCTACACGGACCAG -3' |
|  | R: 5'- TGGTCAGGTTTCATCAAGGAC -3' |
| *Cers2* | F: 5'- AAGTGGGAAACGGAGTAGCG -3' |
|  | R: 5'- ACAGGCAGCCATAGTCGTTC -3' |
| *Cers4* | F: 5'- GGATTAGCTGATCTCCGCAC -3' |
|  | R: 5'- CCAGTATGTCTCCTGCCACA -3' |
| *Cers6* | F: 5'- AAGCCAATGGACCACAAACT -3' |
|  | R: 5'- TGCTTGGAGAGCCCTTCTAAT -3' |
| *Smpd1* | F: 5'- GTTACCAGCTGATGCCCTTC -3' |
|  | R: 5'- AGCAGGATCTGTGGAGTTG -3' |
| *Smpd2* | F: 5'- AGCAGGATCTGTGGAGTTG -3' |
|  | R: 5'- CTCCAGCCATGAAGCTCAAC -3' |
| *Smpd3* | F: 5'- CCTGACCAGTGCCATTCTTT -3' |
|  | R: 5'- AGAAACCCGGTCCTCGTACT -3' |
| *Smpd4* | F: 5'- ACCTGGCCCTCAATCCATTTG -3' |
|  | R: 5'- ATAGGCACAGTCCGAAGTACG -3' |
| *Tgfb* | F: 5'- CCACCTGCAAGACCATCGAC -3' |
|  | R: 5'- CTGGCGAGCCTTAGTTTGGAC -3' |
| *Col3a1* | F: 5'- TAGGACTGACCAAGGTGGCT -3' |
|  | R: 5'- GGAACCTGGTTTCTTCTCACC -3' |
| *Col4a1* | F: 5'- CACATTTTCCACAGCCAGAG -3' |
|  | R: 5'- GTCTGGCTTCTGCTGCTCTT -3' |
| *Col4a2* | F: 5'- GCCCTGTAGTCCTGGGAATC -3' |
|  | R: 5'- CCAGTGCTACCCGGAGAAA -3' |
| *Col5a2* | F: 5'- CATGGAGAAGGTTTCCAAATG -3' |
|  | R: 5'- AAAGCCCAGGAACAAGAGAA -3' |
| *Col12a12* | F: 5'- AATTGCCTCCACACCTTCAC -3'  R: 5'- TCACCAAGCTGCTCATCAAC -3' |
| *Tnfa* | F: 5'- CCACCACGCTCTTCTGTCTAC -3' |
|  | R: 5'- AGGGTCTGGGCCATAGAACT -3' |
| *Pail* | F: 5'- TTCAGCCCTTGCTTGCCTC -3' |
|  | R: 5'- ACACTTTTACTCCGAAGTCGGT -3' |
| *Ccl2* | F: 5'- TTAAAAACCTGGATCGGAACCAA -3' |
|  | R: 5'- GCATTAGCTTCAGATTTACGGGT -3' |
| *Ccl3* | F: 5'- TTCTCTGTACCATGACACTCTGC -3' |
|  | R: 5'- CGTGGAATCTTCCGGCTGTAG -3' |
| *Il1b* | F: 5'- GCAACTGTTCCTGAACTCAACT -3' |
|  | R: 5'- ATCTTTTGGGGTCCGTCAACT -3' |
| *Nlrp3* | F: 5'- ATTACCCGCCCGAGAAAGG-3' |
|  | R: 5'-CATGAGTGTGGCTAGATCCAAG-3' |
| *Pycard* | F: 5'- TGCTTAGAGACATGGGCTTAC -3' |
|  | R: 5'- CAATGAGTGCTTGCCTGTG -3' |
| *Gapdh* | F: 5'-AGGTCGGTGTGAACGGATTTG-3' |
|  | R: 5'-TGTAGACCATGTAGTTGAGGTCA-3' |
| *Actin* | F: 5'-GGCTGTATTCCCCTCCATCG-3' |
|  | R: 5'-CCAGTTGGTAACAATGCCATGT-3' |
| 18s | F: 5'-ATTGGAGCTGGAATTACCGC-3' |
|  | R: 5'-CGGCTACCACATCCAAGGAA-3' |
